# Lytic archaeal viruses infect abundant primary producers in Earth’s crust

**DOI:** 10.1038/s41467-021-24803-4

**Published:** 2021-07-30

**Authors:** Janina Rahlff, Victoria Turzynski, Sarah P. Esser, Indra Monsees, Till L. V. Bornemann, Perla Abigail Figueroa-Gonzalez, Frederik Schulz, Tanja Woyke, Andreas Klingl, Cristina Moraru, Alexander J. Probst

**Affiliations:** 1 Department of Chemistry, Environmental Microbiology and Biotechnology (EMB), Group for Aquatic Microbial Ecology, University of Duisburg-Essen, Essen, Germany; 2 DOE Joint Genome Institute, Berkeley, CA USA; 3 Plant Development & Electron Microscopy, Biocenter LMU Munich, Planegg-Martinsried, Germany; 4 Institute for Chemistry and Biology of the Marine Environment (ICBM), Carl-von-Ossietzky-University Oldenburg, Oldenburg, Germany; 5 Present Address: Department of Biology and Environmental Science, Centre for Ecology and Evolution in Microbial Model Systems (EEMiS), Linnaeus University, Kalmar, Sweden

**Keywords:** Microbial ecology, Biofilms, Virology

## Abstract

The continental subsurface houses a major portion of life’s abundance and diversity, yet little is known about viruses infecting microbes that reside there. Here, we use a combination of metagenomics and virus-targeted direct-geneFISH (virusFISH) to show that highly abundant carbon-fixing organisms of the uncultivated genus *Candidatus* Altiarchaeum are frequent targets of previously unrecognized viruses in the deep subsurface. Analysis of CRISPR spacer matches display resistances of *Ca*. Altiarchaea against eight predicted viral clades, which show genomic relatedness across continents but little similarity to previously identified viruses. Based on metagenomic information, we tag and image a putatively viral genome rich in protospacers using fluorescence microscopy. VirusFISH reveals a lytic lifestyle of the respective virus and challenges previous predictions that lysogeny prevails as the dominant viral lifestyle in the subsurface. CRISPR development over time and imaging of 18 samples from one subsurface ecosystem suggest a sophisticated interplay of viral diversification and adapting CRISPR-mediated resistances of *Ca*. Altiarchaeum. We conclude that infections of primary producers with lytic viruses followed by cell lysis potentially jump-start heterotrophic carbon cycling in these subsurface ecosystems.

## Abstract (web summary)

Little is known about viral-host interactions in the continental subsurface. Here, the authors use a combination of metagenomics, fluorescence in situ hybridization, and electron microscopy to show infections of abundant C-fixing subsurface archaea by lytic viruses.

## Introduction

Earth’s continental subsurface harbors 2–6 × 10^29^ prokaryotic cells^1,2^, which represent a major component of life’s diversity on our planet^3,4^. Among these organisms are some of the most enigmatic archaea, including Aigarchaeota, Asgard archaea, Altiarchaeota, and members of the DPANN radiation^5–8^. Although the ecology and diversity of subsurface microorganisms has been under investigation in several studies, the fundamental question relating to how microbial diversity and composition in the deep subsurface change with virus infection remains mostly unanswered. Viruses have long been recognized as major drivers of microbial diversification^9^, yet little is known about their lifestyle, activity, and impact on oligotrophic subsurface ecosystems. Recent evidence demonstrated high numbers of virus–cell ratios in marine subsurface sediments^10^, suggesting ongoing viral proliferation in the deep biosphere. In oceanic surface sediments below 1000 m depth, virus-mediated lysis of archaea was estimated to be a major contributor to carbon release thus affecting global biogeochemical cycles^11^. In addition, pronounced morphological diversity of bacteriophages with presumably lytic representatives has been found in granitic groundwater of up to 450 m depth^12^, and might be the result of recombination events, horizontal gene transfer, and lysogeny known to shape microbial communities of the subsurface^13^. The recent recovery of two novel bacteriophage genera with lytic genes from groundwater highlights the potential of subsurface environments for being huge reservoirs of previously unknown viruses^14^. Furthermore, a study on predominant *Halanaerobium* spp. from anthropogenic subsurface communities (hydraulically fractured wells) suggested long-term host–virus dynamics, extensive viral predation, and adaptive host immunity based on clustered regularly interspaced short palindromic repeats (CRISPR) spacer to protospacer matches^15^. CRISPR systems function as defense mechanisms for bacteria and archaea against mobile genetic elements (MGEs), including viruses^16^. The CRISPR locus is usually flanked by *cas* genes and interspaced by short variable DNA sequences termed spacers^16^ previously acquired from invading MGEs. The diversification of CRISPR-Cas immunity in the host over geographical distances and time due to preceding viral infections and protospacer mutations has been well-documented, e.g., for *Sulfolobus islandicus*^17^.

Oligotrophic anaerobic subsurface environments can be populated by a variety of different microorganisms, some of them belonging to the phylum Altiarchaeota^7,18^. In fact, these organisms can reach high abundances in their ecosystems with up to 70% of the total community in the aquifer or with up to 95% within the biofilm (BF) they form^19,20^. Members of the genus *Candidatus* Altiarchaeum—the best-studied representative being *Ca*. A. hamiconexum^7^—occur in anoxic subsurface environments around the globe^21,22^ and fix carbon via the reductive acetyl-CoA (Wood–Ljungdahl) pathway^7^. In certain ecosystems, *Ca*. Altiarchaea form nearly pure BFs, which are kept together by filamentous cell surface appendages called hami (singular: hamus)^23^. Studies to date demonstrated symbiotic relationships of *Ca*. Altiarchaea with bacterial partners *Thiothrix* sp.^24^ and *Sulfuricurvum* sp.^25^, but also a co-occurrence with the episymbiont *Ca*. Huberiarchaeum crystalense, belonging to the DPANN clade, has been recently reported^18,26^. A single transmission electron micrograph and the presence of CRISPR systems led to speculations on the existence of *Ca*. Altiarchaeum viruses in the subsurface^27^. However, mesophilic archaeal viruses from the deep terrestrial subsurface remain highly enigmatic, despite the fact that mesophilic archaeal genomes contain more MGEs than their thermophilic counterparts^28^. The knowledge gap on archaeal viruses is fostered by a lack of their genome entries in public databases^29^, missing marker genes for viruses^30^ and a bias toward viruses related to economical, medical, or biotechnological activities^31^. In addition, only ~150 archaeal viruses have been isolated and described to date^32^. Recent exhaustive metagenomic surveys aided the discovery of novel archaeal viruses^33^ from multiple ecosystems, including the ocean^34,35^, hot springs^36–38^, and soils^39,40^, and eventually allowed targeting and visualization of an uncultivated virus based on its genome^41^. More recently another correlative phageFISH imaging approach was used to visualize bacteriophages within sponge tissue^42^.

Due to their worldwide distribution and high abundance as the main primary producer in certain continental subsurface ecosystems, *Ca*. Altiarchaea represent the ideal model genus for studying viruses and their infection mechanisms of mesophilic microorganisms in the subsurface. This is especially relevant because the extent to which lytic infections occur in the continental subsurface is unknown, and lysogeny is assumed to be the predominant viral strategy^43^. In this work, we use metagenomics to predict viruses that infect *Ca*. Altiarchaea in subsurface ecosystems at four different sites across three continents (Europe, Asia, North America). Using virus-targeted direct-geneFISH (virusFISH), we visualize and characterize the most abundant putative virus from a sulfidic spring in Bavaria, Germany, providing novel insights into the lytic lifestyle of an uncultivated virus, whose genome shows little homologies with sequences in public databases and carries no viral hallmark genes. Our analyses further demonstrate the diversification of CRISPR systems of *Ca*. Altiarchaea along with a decline in virus abundances over 6 years. We conclude that the kill-the-winner theorem can play an important role in evolutionary and ecosystem processes of the deep biosphere.

## Results

### Globally distributed *Ca*. Altiarchaea have complex CRISPR systems with conserved direct repeat (DR) sequences

**Fig. 1:**
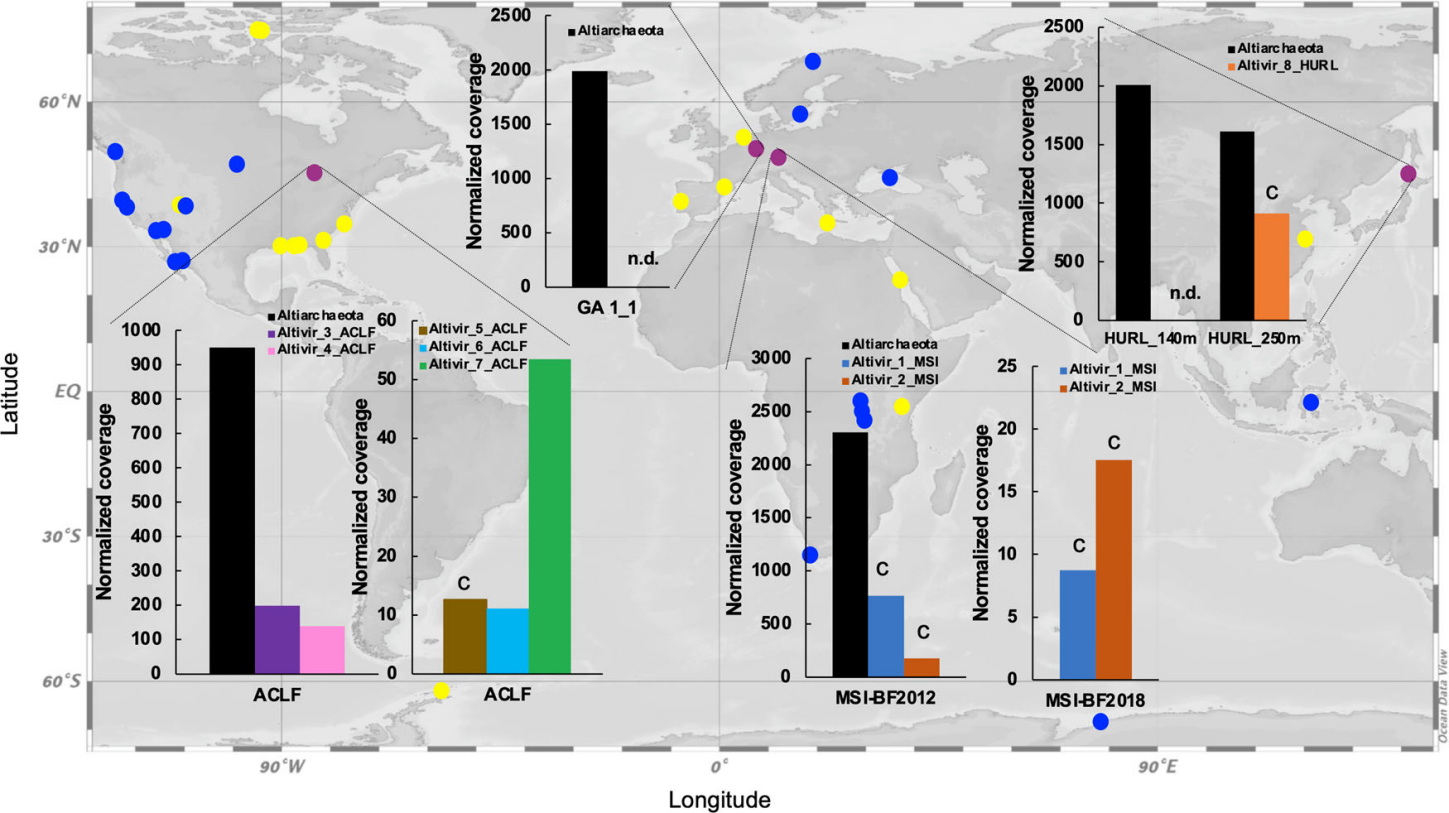
Global distribution of abundant Altiarchaeota (group Alti-1) and their predicted viruses in Altiarchaeota hot spots. Distribution analysis is based on 16S rRNA gene sequencing (yellow dots) and the detection of *hamus* genes in metagenomes from IMG (blue and purple dots). Purple dots correspond to the four investigated sites of this study. Normalized host and virus coverage are given for the four subsurface habitats: Alpena County Library Fountain (ACLF, Michigan, USA), Horonobe Underground Research Laboratory (HURL, Japan), Mühlbacher Schwefelquelle, Isling (MSI, Germany), and Geyser Andernach (GA, Germany). Percent relative abundance of dominant Altiarchaeota compared to other community members is shown in Supplementary Table 1. Only Altivir_1_MSI and Altivir_2_MSI obtained from biofilm (BF) samples are shown. The letter C indicates circular genomes. n.d. none detected. World map has been generated using Ocean Data View v.5.3.0^117^. Color explanation of bars representing normalized coverage: orange-red = Altivir_1_MSI (virus), blue = Altivir_2_MSI, purple = Altivir_3_ACLF, rose = Altivir_4_ACLF, brown = Altivir_5_ACLF, light blue = Altivir_6_ACLF, green = Altivir_7_ACLF, and black = Altiarchaeota (host).

Screening of 16S ribosomal RNA (rRNA) datasets and metagenomes within IMG^44^ confirmed a global distribution of organisms belonging to the phylum Altiarchaeota (Fig. 1). We performed metagenomic analyses of four terrestrial subsurface ecosystems ranging from 37 to 352 m below ground that showed high abundance of the genus *Ca*. Altiarchaeum (Supplementary Table 1), previously also termed Alti-1^21^. These ecosystems included (i) an anoxic aquifer accessible through an artesian well (Mühlbacher Schwefelquelle, Isling, MSI)^25^ sampled in 2012 and 2018, (ii) a high-CO_2_ geyser (Geyser Andernach, GA)^45^ both located in Germany, (iii) a sulfidic spring in the US (Alpena County Library Fountain, ACLF)^46^, and (iv) a deep underground laboratory in Japan (Horonobe Underground Research Laboratory, HURL) at 140 and 250 m depth^22^. All eight genomes of *Ca*. Altiarchaeum (Supplementary Data 1) carried genetic information for type I-B CRISPR-Cas immunity including proteins Cas5, Cas7, and Cas8a. Although other Cas proteins were also present in the assembly but remained unbinned, we were able to confirm the presence of Cas3 (Supplementary Data 1) and Cas6^7^ on scaffolds with the taxonomic annotation of Altiarchaea for the MSI site. Proteins of a type III CRISPR-Cas immunity were found at the ACLF, HURL, and MSI site, including repeat associated mysterious proteins (Cmr) of types III-B and III-C and Csm proteins of the type III-A system (Supplementary Data 1). Confidence in binning CRISPR arrays and assigning DR sequences to *Ca*. Altiarchaea arose from the 16-fold to 146-fold higher abundance of these organisms (and their CRISPR arrays) in the ecosystems than other microbes (Supplementary Fig. 1^7,22,46^). In addition, two versions of a CRISPR DR sequence assigned to *Ca*. Altiarchaea were highly conserved across these ecosystems (Supplementary Fig. 2, Supplementary Data 1). DR sequence 1 occurred in all four ecosystems, whereas DR sequence 2 was only found at the HURL and the ACLF site (Supplementary Data 1). While all DR sequences from the four sites were previously unknown in the CRISPRmap database, DR sequence 1 in orientation 1 and DR sequence 2 in both orientations structurally resembled motif 13 and 12 of the database, respectively. All four sequences form thermodynamically favorable secondary structures and carry an AAA(N) motif (Supplementary Fig. 2), indicating that both strands of the CRISPR array could theoretically be transcribed.

### Eight novel viral clades with genome relatedness across continents show infection histories with Altiarchaeota

**Fig. 2:**
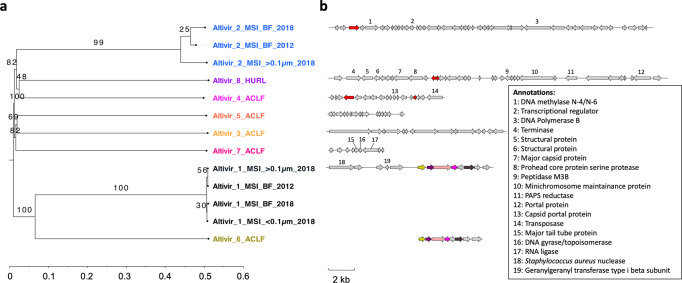
Phylogenomic genome-BLAST distance phylogeny (GBDP) tree of *Ca.* Altiarchaeum viruses and viral proteins clusters. **a** The tree was inferred using the distance formula D0 yielding average support of 69%. The numbers above branches are GBDP pseudo-bootstrap support values from 100 replications. The branch lengths of the resulting VICTOR^94^ trees are scaled in terms of the respective distance formula used. The tree shows that the eight predicted viral genomes were assigned to the same family, to eight different genera, and ten species (which is the result of having multiple genomes included for Altivir_1_MSI and Altivir_2_MSI). **b** Protein clustering across all viruses revealed that Altivir_1_MSI_>0.1 µm_2018 and Altivir_6_ACLF, originating from different continents, have five protein clusters (2–6) in common. Altivir_2_MSI_BF_2012, Altivir_4_ACLF, and Altivir_8_HURL shared only one protein cluster (1). Open reading frames of the viral genomes were predicted using Prodigal v.2.6.3^74^ and further translated with the R package seqinr v.3.6.-1. Colors indicate shared protein clusters between genomes and numbers show nonhypothetical consensus annotations according to Supplementary Data 3. Color code for viral genomes: Altivir_1_MSI = black, Altivir_2_MSI = blue, Altivir_3_ACLF = yellow, Altivir_4_ACLF = pink, Altivir_5_ACLF = orange, Altivir_6_ACLF = olive-green, Altivir_7_ACLF = pink-red, and Altivir_8_HURL = purple.

Using matches of Altiarchaeota spacers to protospacers, we were able to identify 13 predicted viral genomes (termed *Ca*. Altiarchaeum virus Altivir_#, here further referred to as Altivir_# for short) in three out of the four sampling sites (Supplementary Table 2). No viruses targeted by *Ca*. Altiarchaea spacers could be predicted for GA and the HURL sites at 140 m depth. Only two out of the 13 predicted viral genomes, i.e., the 20.8-kb long Altivir_2_MSI_BF_2012 and the 22.6-kb long Altivir_8_HURL, had hits in the Virus Orthologous Groups Database (VOGDB, Supplementary Table 2), carried viral hallmark genes, and were circular, prompting us to classify them as viruses. The others were designated as putative viruses according to our classification scheme (Supplementary Fig. 3). All 13 (putative) viruses were categorized as lytic viruses according to VirSorter^47^. Four predicted viruses were circular and thus complete in their genome sequence (Fig. 1, Supplementary Table 2). The 13 viral genomes formed eight monophyletic clades based on VICTOR analysis (Fig. 2), representing potentially eight individual genera (VICTOR threshold for genus was 15.8% nucleotide based intergenomic similarity). Viral genomes in the Altivir_1_MSI and Altivir_2_MSI clades were recovered in all sampled time points from the MSI site (Supplementary Table 2), both in the cellular and virus enriched fractions. The Altivir_2_MSI genomes recovered from the virus enriched fraction (MSI_<0.1 µm_2018) were fragmented and thus excluded from further analysis. Intergenomic pairwise similarities for the four Altivir_1_MSI varied between 99.3 and 99.7% (Supplementary Fig. 4), and this clade thus represens a single viral species (the threshold for species demarcation was 95% similarity). The three Altivir_2_MSI genomes had similarities between 87.0 and 96.1% and represented two viral species (Supplementary Fig. 4). Using vConTACT, Altivir_1_MSI formed a cluster with Altivir_6_ACLF, a putative virus from ACLF, with whom it shared five protein clusters (Fig. 2, Supplementary Fig. 5). This relatedness between viruses from highly distant subsurface ecosystems was further supported by the VICTOR analysis, which placed them in the same monophyletic clade (Fig. 2). All remaining viruses apart from Altivir_1_MSI, Altivir_2_MSI and Altivir_6_ACLF were designated by vConTACT as unclustered singletons. Only one protein cluster was shared between Altivir_2_MSI, Altivir_4_ACLF, and Altivir_8_HURL, and no protein clusters between the remaining three viruses, indicating that all these viruses are distant from each other (Fig. 2). In total, these eight viral genera were affiliated to seven vConTACT viral clusters (Altivir_1_MSI/Altivir_6_ACLF grouped together), which were unrelated with previously published viral genomes in the RefSeq94 database according to vConTACT criteria (Supplementary Fig. 5). However, based on the phylogenetic analysis of the DNA polymerase B (for detailed annotations of viral proteins please see below; Supplementary Fig. 6), Altivir_2_MSI is likely related to *Tectiviridae*, a dsDNA virus family in the *Varidnaviria* realm. The presence of a capsid portal protein in Altivir_4_ACLF (Supplementary Fig. 7), a major tail tube protein in Altivir_7_ACLF (Supplementary Fig. 8), and a terminase as well as a portal protein in Altivir_8_HURL (Supplementary Fig. 9) indicate that these three viruses belong to the *Duplodnaviria* realm. Phylogenetic analysis of the aforementioned proteins suggests that Altivir_7_ACLF and Altivir_8_HURL potentially belong to the *Caudovirales* order. Altivir_4_ACLF is distantly related to eukaryotic viruses from *Herpesvirales*, also suggesting a relationship with *Caudovirales*.

Comparing the genomes of Altivir_1 and Altivir_2 individually across the samples from 2012 and 2018, we identified different developments of the two viral genomes. Altivir_2_MSI clade presented gene content variations between the genomes from different samples (Supplementary Fig. 10), which agrees with the fact that it represents two viral species. By contrast, Altivir_1_MSI accumulated multiple single-nucleotide polymorphisms and only represented strain level variations of the same species (Supplementary Fig. 10, Supplementary Data 2).

Host–virus ratios (considering *Ca*. Altiarchaeum to be the host) based on metagenome read mapping varied greatly (between 1.8 and 301.9; Supplementary Table 2) with the smallest ratios of 1.8 for Altivir_8_HURL, followed by 2.3 for Altivir_1_MSI_>0.1 µm_2018, 2.8 for Altivir_2_MSI_>0.1 µm_2018, and 3.0 for Altivir_1_MSI_BF_2012. Abundance of in the planktonic fraction (>0.1 µm) suggests high concentrations of *Ca*. Altiarchaeum BF on the 0.1 µm filter membrane during the filtration process. Relative abundance of viruses based on normalized coverage ranged between 9 (Altivir_1_MSI_BF_2018) and 913 (Altivir_8_HURL, Fig. 1, Supplementary Table 2). The genomes of Altivir_2_MSI_BF_2018 and Altivir_8_HURL carried short CRISPR arrays with one spacer each, but spacers from these mini-CRISPR arrays did not match other viruses in the respective ecosystems.

Several viral proteins could be functionally annotated (see Fig. 2B, Supplementary Table 2, Supplementary Data 3), belonging mostly to the circular Altivir_8_HURL genome. Altivir_8_HURL, bearing 36 genes, encoded for an auxiliary metabolic gene (AMG), namely the phosphoadenosine phosphosulfate reductase (PAPS, *CysH*) (phmmer, *e*-value: 6.1e−48), which probably facilitates assimilatory sulfate reduction in the host, and is a common AMG of many viruses^48^. Altivir_1_MSI, of particular interest for this study because it was highly abundant in the MSI_BF-2012 metagenome, recruited many spacer hits and only 2 out of its 14 proteins could be annotated with enough certainty (Fig. 2, Supplementary Table 2, Supplementary Data 3). These included a nuclease and a geranylgeranyl transferase. In sum, only 11.9% of the 159 proteins across all Altivir genomes have a putative function assigned rendering the remaining genes of yet unknown function as genetic dark matter (Fig. 2, summary of annotations in Supplementary Table 2).

### VirusFISH reveals a lytic lifestyle for Altivir_1_MSI

**Fig. 3:**
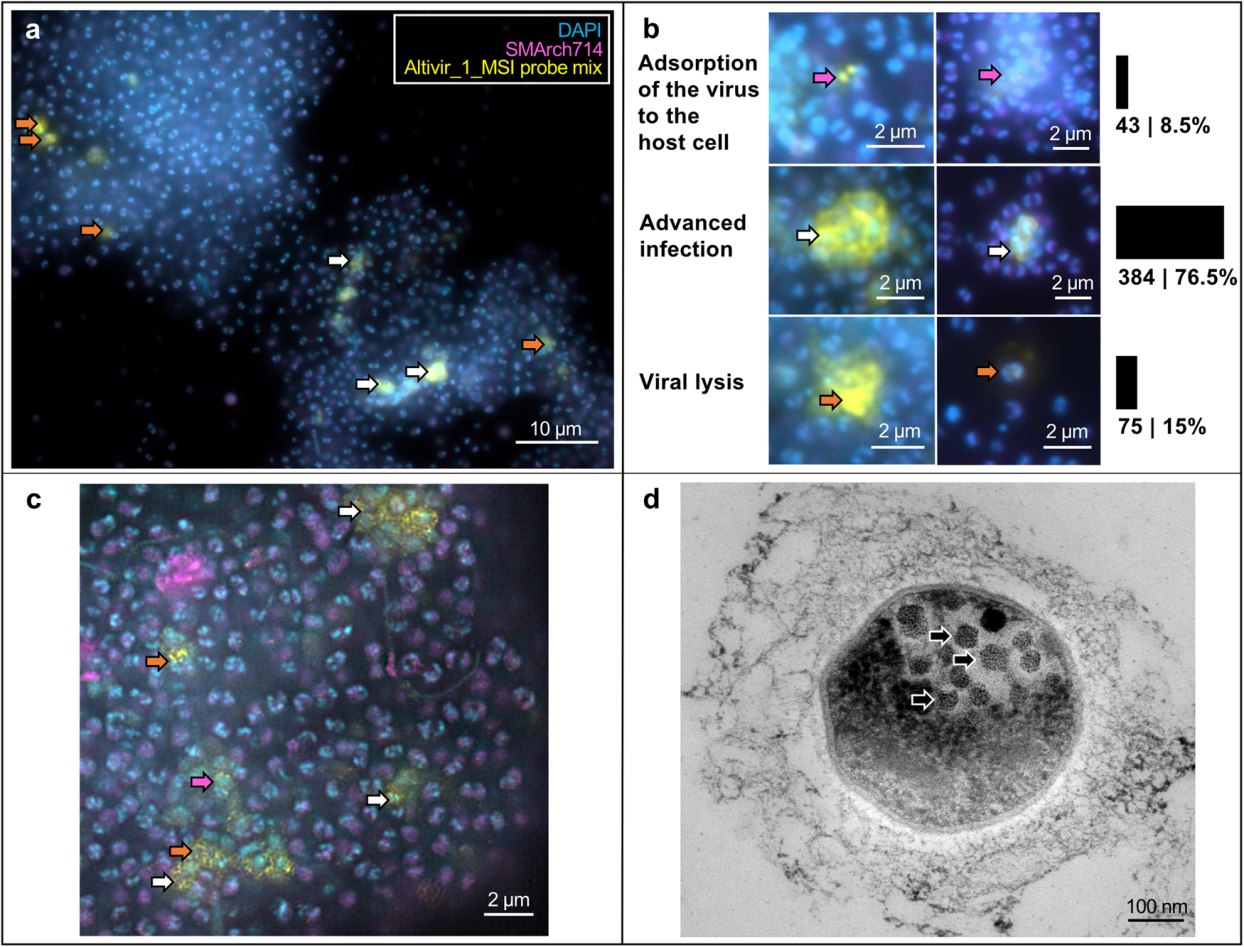
Visualization and quantification of *Ca.* Altiarchaeum virus Altivir_1_MSI-infected and noninfected Altiarchaeota biofilm (BF) cells from MSI site. For all virusFISH experiments shown here, an Altivir_1_MSI probe was used for detecting viral infections. BF material was visualized with filter sets for DAPI (blue, cells), ATTO 488 (purple, 16S rRNA signal), and Alexa 594 (yellow, viral genomes), and merged for analysis and display purposes. **a** VirusFISH displays the interactions between Altiarchaeota cells and their virus shown as yellow dots. For unmerged imaging data, see Supplementary Fig. 11. Scale bar: 10 µm. **b** Different infection stages with Altivir_1_MSI. The enumeration performed with a regular epifluorescence microscope was based on 18,411 archaeal cells and categorized into three infection stages. For the categorization we used in total 18 Altiarchaeota biofilms, whereby 17 biofilms were treated with the Altivir_1_MSI probe (*n* = 17) and one biofilm was treated with a *Metallosphaera* sp. virus probe (*n* = 1). Purple arrows indicate exemplary viruses that attach to host’s cell surface, white arrows show advanced infections, and orange arrows bursting cells with free viruses. Scale bars: 2 µm. **c** Coupling virusFISH with structured illumination microscopy showed extracellular signals of tiny fluorescently labeled viral particles attaching to Altiarchaeota’s cell surface but also in a free state as presumably released virions (*n* = 1). Scale bar: 2 µm. **d** Transmission electron microscopy revealed intracellular virus-like particles (*n* = 3). Scale bar: 100 nm.

We selected the in silico predicted Altivir_1_MSI viral clade for visualization by virusFISH, due to its high abundance at the MSI site, and despite the lack of viral hallmark genes. VirusFISH with a probe containing 11 double stranded polynucleotides was successfully implemented to visualize the distribution of the circular genome of Altivir_1_MSI within altiarchaeal BF (Fig. 3A, Supplementary Fig. 11). In contrast to the negative control with a nonmatching probe (Supplementary Fig. 12), our target probes enabled us to detect altiarchaeal cells containing Altivir_1_MSI. Multiple cells were surrounded by halo signals, corresponding to a viral burst^49^ and providing evidence for Altivir_1_MSI being an active virus and lysing *Ca*. Altiarchaeum cells.

A total of 18,411 altiarchaeal cells and 502 viral infections (colocalization of Altivir_1_MSI and *Ca*. Altiarchaea signals) across 18 samples/BF flocks were analyzed via fluorescence microscopy and categorized into three main infection stages: (i) viral adsorption to the host cells (8.5%), (ii) advanced infection with intracellular virus signals and ring-like signals around the cells (76.5%), and (iii) cell lysis with bursting cells and release of virions (15%) (Fig. 3B). Super-resolution microscopy further showed extracellular signals of small fluorescently labeled particles, which we interpret as individual viral particles (Fig. 3C). This observation was further supported by ultrathin sectioning and transmission electron microscopy, which revealed many intracellular virus-like particles associated with *Ca*. Altiarchaea cells. These particles had an average diameter of 50 nm (SD ± 7 nm), as measured across 11 host cells (Fig. 3D). The high percentage of cell lysis associated with the virus signals along with the high abundance of the virus in the planktonic and viral fraction suggests a lytic lifestyle for Altivir_1_MSI.

### Spatio-temporal heterogeneity of predicted infections and CRISPR-Cas mediated immunity of *Ca*. Altiarchaea

**Fig. 4:**
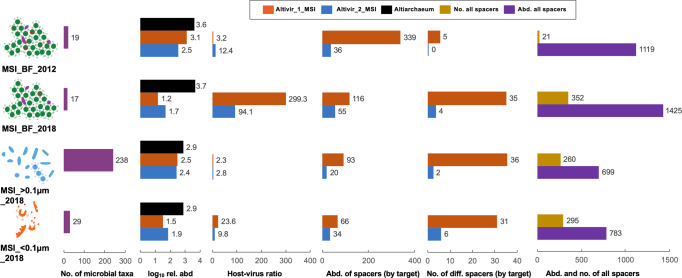
Development of host–virus and spacer dynamics from 2012 to 2018 based on metagenomics from the Mühlbacher Schwefelquelle, Isling (MSI). Predicted viruses Altivir_1_MSI and Altivir_2_MSI became less abundant from 2012 to 2018. Data are presented for biofilms (BF) from 2012 and 2018 as well as the planktonic fraction (>0.1 µm) and the viral fraction (<0.1 µm). Considering the BFs, total spacer abundance and numbers increased from 2012 to 2018 while those matching Altivir_1_MSI decreased in abundance but increased in numbers. Number (no.) of microbial taxa refers to the number of different prokaryotes in a sample detected via *rpS3* rank abundance curves (normalized by sequencing depth). Host–virus ratio is calculated from host and virus coverage based on read mapping. Abundance and number of different spacers were normalized to minimum relative abundance (rel. abd.) of the host based on read mapping. Color definition of bars: violet = number of microbial taxa, orange-red = features of Altivir_1_MSI, blue = features of Altivir_2_MSI, black = log_10_ relative abundance of Altiarchaeum, ocher = number of all spacers, and purple = abundance of all spacers.

To investigate the development of virus immunities over time, we compared the publicly available metagenome from MSI (taken in 2012) to a newly sequenced BF sample from 2018. We also analyzed the planktonic microbiome (>0.1 µm) and a viral fraction (<0.1 µm), i.e., after 0.1 µm filtration and FeCl_3_ precipitation. We compared the change in relative abundance (normalized coverages) of *Ca*. Altiarchaeum, Altivir_1_MSI, Altivir_2_MSI, and *Ca*. Altiarchaeum CRISPR spacers across these samples (Fig. 4) with *Ca*. Altiarchaeum being the most dominant microbe in each sample (Supplementary Fig. S1). While the planktonic microbiome showed a tremendous diversity based on *rpS3* sequences (238 different organisms, Fig. 4), the diversity was quite restricted with 19 and 17 organisms in MSI_BF_2012 and MSI_BF_2018, respectively. Please note that there is a difference between the numbers of organisms detected in the rank abundance curves (Supplementary Fig. 1) and those reported in Fig. 4 as the latter were normalized to read abundance to ensure comparability.

Both predicted viral genomes (Altivir_1_MSI_BF_2012, Altivir_2_MSI_BF_2012) declined in abundance when comparing the BF sample from 2018 to the sample from 2012, and at the same time the host-virus ratio increased (Fig. 4). While Altivir_1_MSI was more abundant in 2012 (host–virus ratio = 3.2) compared to Altivir_2_MSI relative to its host (host–virus ratio = 12.4), the pattern reversed in 2018 for the BF sample (host–virus ratio = 299.3 compared to 94.1 for Altivir_1_MSI and Altivir_2_MSI, respectively). Both, the total spacer abundance and the spacer diversity increased from 2012 to 2018 in BF samples, i.e., from 21 to 352 (number of different spacers) including an ~20% increase in the number of spacer clusters that were singletons in the dataset (Supplementary Fig. 13) and from 1119 to 1425 (abundance of spacers). The abundance of spacers matching the genome of Altivir_1_MSI_BF_2012 decreased from 339 to 116 spacers, whereas it increased from 36 to 55 for Altivir_2_MSI_BF_2012 in BF samples from 2012 to 2018. For both targets the number of different matching spacers increased over time (Supplementary Fig. 14), in line with the development of the total spacers in this ecosystem (Fig. 4). Because planktonic *Ca*. Altiarchaeum cells (diameter: 0.4-0.6 µm) cannot pass the 0.1 µm pore-size filters, it is more likely that lysed *Ca*. Altiarchaeum cells ended up in the <0.1 µm fraction in 2018 allowing binning of their population genome including the CRISPR system with low complexity of spacers from this fraction. The MSI_>0.1 µm_2018 fraction and the MSI_<0.1 µm_2018 contained about half the number of total spacers of the MSI_BF_2018 sample, although the number of different spacers displayed less variability. Spacers from these samples hitting the viral targets were often reduced in abundance compared to BF-derived CRISPR spacers (Fig. 4). MSI_BF_2018 had the most unique total spacer clusters (2599, Supplementary Fig. 15A) and also the most unique ones matching Altivir_1_MSI (Supplementary Fig. 15B) and Altivir_2_MSI as displayed as Venn diagrams in Supplementary Fig. 15C. The overlap of spacer clusters between years and samples was rather moderate (≤52 spacer clusters in common).

**Fig. 5:**
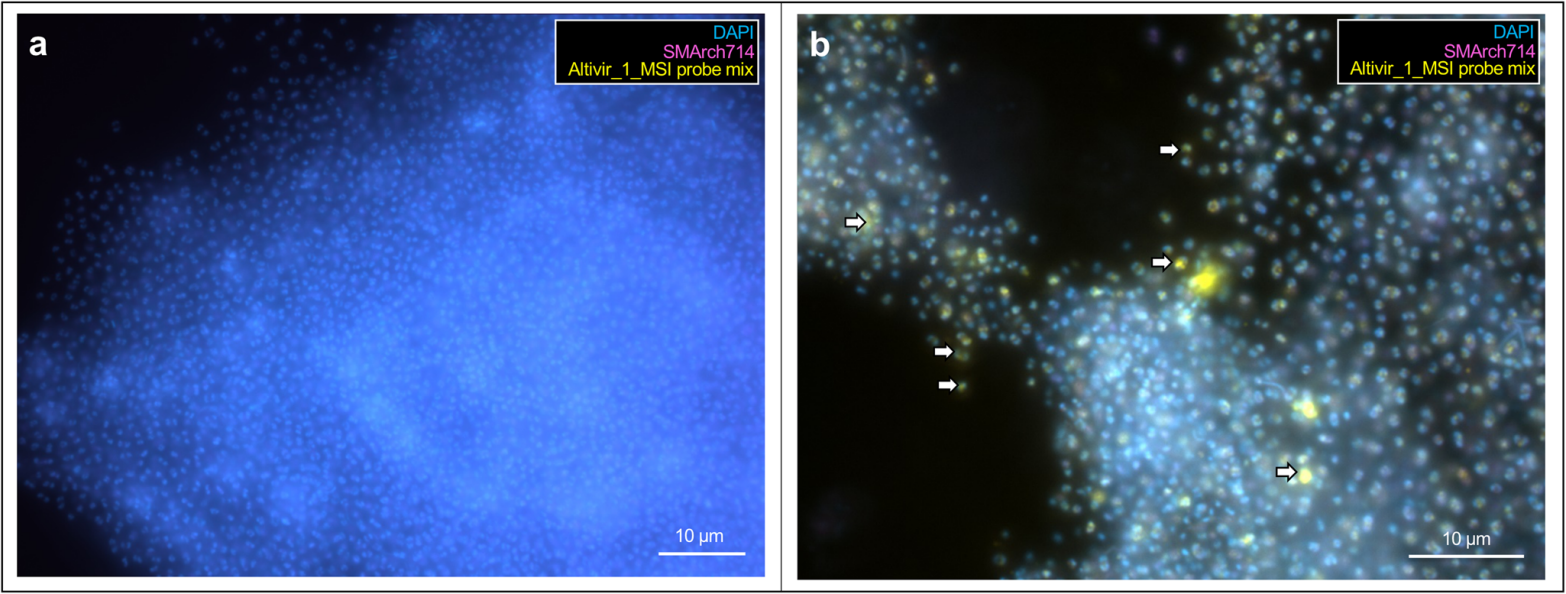
VirusFISH of two individual Altiarchaeota biofilm (BF) flocks depicting **a**) a dense BF flock without infections and **b**) one highly infected BF flock. For all virusFISH experiments, an Altivir_1_MSI probe was used for detecting viral infections. White arrows indicate exemplary virus–Altiarchaeota interactions (advanced infections). BF material was analyzed with filter sets for DAPI (blue, cells), ATTO 488 (purple, 16S rRNA signal), and Alexa 594 (yellow, viral genomes), and then the different fluorescent channels were merged for analysis and display purposes. For unmerged imaging data, see Supplementary Figs. 16 and 17. Scale bars: 10 µm.

Congruent with the decline in relative abundance of Altivir_1_MSI based on metagenomic analysis, we also observed heterogeneous infections of BF flocks via imaging. Some BF showed no infection with Altivir_1_MSI at all (Fig. 5A, Supplementary Fig. 16), which aligns well with the decrease of the virus in the metagenomic data of the BF from 2012 to 2018. By contrast, we observed very few BF flocks that showed an extremely high infection and accumulation of rod-shaped microorganisms (Fig. 5B, Supplementary Figs. 17 and 18). The observed heterogeneity of infections in BF supports the aforementioned heterogeneity related to CRISPR resistances against Altivir_1_MSI with high spacer diversity dominated by singletons.

## Discussion

Although life in the deep subsurface contributes significantly to overall biomass and microbial biodiversity on our planet, its low accessibility leaves host–virus interactions—especially those of uncultivated hosts—highly enigmatic. Detection of novel and uncultivated viruses missing conserved sets of hallmark genes has been an ongoing challenge in viromics^50^. Using a combination of bioinformatics and virusFISH, we were able to visualize infections of Altiarchaeota with a hitherto unknown, presumably lytic virus from a subsurface ecosystem. We expanded the diversity of this virus detected in Germany (MSI) by identifying a distantly related viral genome infecting *Ca*. Altiarchaea in North America (ACLF site). This suggests genomic relatedness of archaeal viruses over long distances, a phenomenon known for bacteriophages^51,52^. However, genomic relatedness and global distribution of bacteriophage genomes (and maybe also altiarchaeotal viruses) is in stark contrast to viruses infecting *Sulfolobus islandicus*. Viruses of this thermoacidophilic archaeon displayed a confined geographic distribution based on core gene sequence analysis and CRISPR-based host–virus interactions^53,54^, which demonstrated infections with local viruses for this host, and the geographical structuring of archaeal viral communities was recently extended by investigations of Italian hydrothermal environments^55^.

Altiarchaeota viruses do not represent an exception regarding the many genes that could not be annotated, and thus were classified as unknown ORFs or domains/genes of unknown function. Some proteins such as modification methylases or the phosphoadenosine phosphosulfate reductase were previously also detected on a *Methanosarcina* and other archaeal virus genomes^56,57^. Altiarchaeota can apparently employ two different CRISPR systems (types I and III) with high similarity of their DR sequences across larger geographic distances; although the reasoning behind two independent CRISPR systems remains unclear, an additional type III system might mediate resistance against plasmids carrying matching protospacers but lacking a protospacer-adjacent motif^58^ or against viruses that overcome type I systems as previously described for *Marinomonas mediterranea*^59^ and *Sulfolobus islandicus*^60^. Indeed, archaeal viruses found ways to interfere with CRISPR type III systems by using anti-CRISPR proteins^61,62^. However, we could not detect homologs of these proteins in viruses targeting *Ca*. Altiarchaeum.

Based on the metagenomic data collected in 2012 and 2018, it appears that the host prevailed in the arms race between Altiarchaeaota and the visualized virus (Altivir_1_MSI), whereas the arms race seems to be ongoing as at least parts of the host population still get infected. The number of different spacers matching this virus increased toward 2018 with a prominence of spacer singletons in the metagenome (Supplementary Fig. 13), while the actual abundance of spacers matching Altivir_1_MSI decreased as did the abundance of the viral genome itself. CRISPR spacer diversification might be a successful response to an increasing number of variants in the genome of Altivir_1_MSI (Supplementary Fig. 10) suggesting its mutations. As a trade-off, spacer diversification might allow the host to decrease the total abundance of spacers. We conclude that CRISPR spacers were diversifying to mediate a greater bandwidth of resistances against the virus, which worked in favor for the overall population of Ca. Altiarchaea in this ecosystem. Moreover, the diversity of singleton spacers indicates a very heterogeneous Altiarchaeota population, which might be related to heterogeneous infections of the BF as visualized via virusFISH.

Our dual approach of coupling metagenomics to fluorescence microscopy enabled us to follow precisely the terrestrial subsurface predator–prey relationship of *Ca*. Altiarchaeum and one of its viruses. Our data suggest that the novel identified virus is lytic and challenge the current paradigm that lysogeny prevails in the subsurface^30,43^. Instead, our data indicate that the kill-the-winner theorem—lytic viruses targeting abundant ecosystem key players^63^—also strongly applies to subsurface ecosystems. We currently do not know if this statement can be transferred to other subsurface ecosystems that have low cell counts^2^ as viruses might struggle with finding a new host. However, replication measures of bacteria in the ecosystem with the visualized virus suggest that microbial proliferation is similar to other oligotrophic systems^45^. Lytic infections in subsurface microbial hosts might thus launch heterotrophic carbon cycling similar to the viral shunt in the marine environment^64^. In fact, recent lipidomic analyses coupled to mass-balance calculations provide evidence that subsurface environments dominated by Altiarchaeota are completely fueled by these organisms’ carbon fixation, transferring organic carbon to heterotrophs in the community^65^. This process might be the basis for microbial loops^66^ as we see the accumulation of rod-shaped microbes around Altiarchaeota when they are lysing due to viral attacks (Supplementary Fig. 18).

Here, we provide underpinning evidence derived from metagenomic datasets of three continents for the frequent viral infection of a globally abundant, autotrophic key player of the subsurface carbon cycle. Using virusFISH for visualization, we show that one virus with a lytic lifestyle is even capable of infecting host cells in a dense BF, which is generally known to provide some protection from viral infection^67^. Subsurface ecosystems such as aquifers remain understudied regarding host–virus dynamics because of limited access, low microbial biomass, and limited cultivation success. Our results presented here provide an experimental proof of concept for an ongoing host–virus arms race in the continental subsurface characterized by constant viral infections and cell lysis of subsurface microbes, followed by their own and their viruses’ diversification.

## Methods

### Mining public metagenomes for Altiarchaeota

To get an overview of the global distribution of Altiarchaeota, we searched metagenomes in the IMG/M database^44^ (database accessed in July 2018) for Altiarchaeota contigs using DIAMOND BLASTp (v0.9.22)^68^ with the putative *hamus* subunit (NCBI accession no CEG12198.1) as a query and an *e*-value and length cut-off of 1e−10 and 300 amino acids, respectively. The Altiarchaeota distribution based on 16S rRNA gene sequences was obtained from the SILVA SSU Parc database^69^ based on all 16S rRNA genes classified as Altiarchaeota and for which geographic information was available (July 2018).

To investigate Altiarchaeota–virus relationships, we explored ecosystems where Altiarchaeota comprised the majority of the community. Therefore, metagenomic data from a microbial mat growing in the sulfidic groundwater-fed ACLF (Alpena, MI, USA) were obtained from NCBI’s Sequence Read Archive (SRA) repository^46^ as were metagenome samples from HURL at 140 and 250 m depth (HURL, Hokkaido, Japan)^22^. The analysis was complemented with three metagenomic datasets from CO_2_-enriched groundwater erupted from a cold-water Geyser (Andernach, Middle Rhine Valley, Germany)^45^, and the metagenome of a sulfidic spring MSI in Regensburg, Germany^7^. All BioProject, BioSample accessions, and information on sampling of MSI for various experiments are provided in Supplementary Tables 1, 3, and 4, respectively.

### Resampling of MSI for virusFISH and metagenomic sequencing

BF samples were collected as previously described^20^ from the 36.5 m deep, cold (~10 °C), sulfidic spring MSI in Regensburg, Germany (N 48° 59.142, E 012° 07.636) in January 2019, for which further geological description has been reported elsewhere^70^. Environmental parameters of the sulfidic spring were extensively investigated previously^25^ and remained almost constant over years. In brief, hydrogen sulfide concentration in the spring hole was reported to be 0.85 mg L^−1^ ^70^, dissolved carbon dioxide at 32 mg L^−1^ ^25^, and oxygen at 0.13 mg L^−1^ and later revised to be beyond detection limit of extremely sensitive probes^20,25^. Chemical parameters of the spring water are presented in Supplementary Table 5.

For virusFISH, BF samples were fixed by addition of formaldehyde (3% v/v) and incubation at room temperature for 1 h. For investigating infection stages, BF flocks were gently separated from several bigger flocks by using a pipette tip finally yielding 18 smaller flocks (range 170–5870 µm^2^). BF flocks for all microscopy experiments were subsequently washed three times in 1x phosphate buffered saline (PBS, pH 7.4) followed by dehydration via an ethanol gradient (50%, 70% v/v, and absolute ethanol, 10 min each). Samples were stored in absolute ethanol at −20 °C until further processing.

Three types of samples were collected for metagenomics: (i) BF flocks; (ii) the planktonic community (>0.1 µm pore-size fraction); and (iii) the viruses and lysed cells (<0.1 µm pore-size fraction). Sampling of Altiarchaeota BF flocks for DNA extraction and metagenomic sequencing from the sulfidic spring (MSI) was performed in October 2018. For sampling the unfiltered planktonic microbial community, 70 L of groundwater were filtered onto a 0.1 µm pore-size PTFE membrane filter (Merck Millipore, Darmstadt, Germany). The flow-through was collected in a sterilized container, and a final concentration of 1 mg L^−1^ of iron (III) chloride (Carl Roth, Karlsruhe, Germany) was applied for chemical flocculation for 30 min^71^. Flocculates were filtered onto 5 × 0.2 µm membrane filters (<0.1 µm fraction). DNA was extracted directly from collected BF samples using the RNeasy^®^ PowerBiofilm Kit (Qiagen, Hilden, Germany) using a DNA-conform workflow. DNA from the 0.1 µm and pooled 0.2 µm membrane filters with iron flocculates was extracted using PowerMax Soil DNA Extraction Kit (Qiagen, Hilden, Germany), was precipitated overnight, and cleaned with 70% ethanol. Shotgun metagenome sequencing was conducted within the Census of Deep Life Sequencing call 2018 and performed using the Illumina HiSeq platform at the Marine Biological Laboratory, Woods Hole, MA, USA.

### Detection of viral genomes in metagenomes

Raw shotgun sequencing reads were trimmed and quality-filtered using bbduk (https://github.com/BioInfoTools/BBMap/blob/master/sh/bbduk.sh) and Sickle v.1.33^72^. Read assembly was conducted by using metaSPADes v.3.10^73^ unless stated otherwise. Scaffolds <1 kb length were excluded from further analysis. Genes were predicted using prodigal v.2.6.3^74^ (meta mode) and functional annotations were determined by using DIAMOND v.0.9.9^68^ against UniRef100 (February 2018)^75^. Public Altiarchaeota genomes were retrieved from databases^45^ and further cleaned or rebinned using %GC content, coverage distribution, and taxonomy information^76^, which was necessary for all genomes except for Altiarchaeota from MSI_BF_2012 (Supplementary Table 1). Viral scaffolds > 3 kb were identified by applying a combination of tools as presented in Supplementary Fig. 3A. Predicted viruses were classified into viruses and putative viruses according to the classification system presented in Supplementary Fig. 3B. Viral scaffolds were subsequently checked for mini-CRISPR arrays using default settings of CRISPRCasFinder^77^ as some archaeal viruses can bear mini-CRISPR arrays with 1–2 spacers having likely a role in interviral conflicts^78,79^.

### CRISPR-Cas analysis of Altiarchaeota genomes

*Cas* genes and DR sequences were identified in binned Altiarchaeota genomes via CRISPRCasFinder^77^ and genes were additionally confirmed via searches against UniRef100^75^. CRISPR DR sequences were tested for formation of secondary structures using RNAfold^80^ and checked against the CRISPRmap database (v2.1.3-2014)^81^ for formation of known motifs. The consensus DR sequence was used in both possible orientations to extract host-specific spacers from raw reads by using MetaCRAST^82^ with Cd-hit v.4.6^83^ clustering at 99% identity. Spacers were filtered for minimum and maximum lengths of 20 and 60 nucleotides, respectively. Only spacers that were present on a read that contained at least one complete DR sequence with an exact match to the template were considered. Finally, spacers were further clustered with Cd-hit^83^ at 99% identity and matched to viral protospacers on the compiled output of viral identification tools using the BLASTn—short algorithm with a 80% similarity threshold. For comparing spacer dynamics (total abundance, diversity, and matches to Altivir_1_MSI and Altivir_2_MSI genomes) of 2012 and 2018 samples from MSI, all spacers of the four respective MSI samples were clustered with Cd-hit^83^ at 99% identity and representative sequences of each cluster were matched to representative Altivir_1_MSI and Altivir_2_MSI genomes from the BF of 2012 (Supplementary Table 2). All data arising from spacer counts were normalized by genome abundance of the respective Altiarchaeota genome.

### Functional annotation of proteins and clustering of viral genomes

Coding sequences of predicted viral scaffolds were identified using prodigal (meta mode)^74^.

Furthermore, the proteins predicted by prodigal were grouped into clusters based on BLASTp similarity^84^ and then functionally annotated by searching in several sequence databases and then manually curating and consolidating the results. The databases searched were the following: (i) the NR database (virus only section, https://blast.ncbi.nlm.nih.gov/) from NCBI was searched using BLASTp and DELTA-BLAST (if no BLASTp results were found, ^85^); (ii) the prokaryotic Viruses Orthologous Groups^86^ was searched using HHblits v.3.3.0 (results kept only if the probability was ≥70%)^87^; (iii) the VOGDB^88^ (vog93, April 2019) was searched using HMMER with an *e*-value cut-off of 10^−5^; (iv) the InterPro database 82.0^89^ was searched using InterProScan integrated in Geneious Prime 2020^90^; (v) UniRef100 (Feb. 2018)^75^ was searched using DIAMOND^68^ with an *e*-value of 10^−5^, and (vi) PDB_mmCIFC70_4_Feb, Pfam-A v.32.0, NCBI_Conserved_Domains_v3.16, and TIGRFAMs v15.0 database were searched using HHpred^91,92^ with an *e*-value cut-off of 10^−6^. For the two most abundant predicted *Ca*. Altiarchaeum viruses Altivir_1_MSI and Altivir_8_HURL, we additionally applied DELTA-BLAST^85^ searches against NCBI’s nonredundant protein sequences (nr) and phmmer^93^ against reference proteomes.

Predicted viral scaffolds carrying multiple hits against existing bacterial genomes in UniRef100^75^ and no viral hallmark genes or carrying extensive CRISPR arrays (detected as false positives) were removed from further analyses.

Clustering of entire viral genomes on nucleic acid level was performed with VICTOR^94^ (https://ggdc.dsmz.de/victor.php) using the genome-BLAST distance phylogeny method^95^ with distance formula d0 and OPTSIL clustering^96^. A separate virus clustering was performed using vConTACT v.0.9.11^97,98^ applying the database “ProkaryoticViralRefSeq94”^99^ and visualization of the viral network was performed in Cytoscape version 3.7.2^100^. Intergenomic similarities between viral genomes and the corresponding heatmap were calculated using VIRIDIC accessible through http://viridic.icbm.de with default settings^101^. In order to further investigate the genetic relationship between the *Ca*. Altiarchaeum viruses Altivir_1–Altivir_8, proteins of all viral genomes were clustered by first performing an all against all BLASTp with an *e*-value cut-off of 10^−5^ and a bitscore threshold of 50. Then, the results were loaded into the mcl program with the parameters -l --abc. The genomic maps were plotted using the genoPlotR v.0.8.9^102^ package of the R programming environment v.3.5.2^103^.

### Single-gene phylogenetic trees

Phylogenetic trees were constructed for viral hallmark proteins identified in different *Ca*. Altiarchaeum viruses: (i) DNA polymerase B from Altivir_2_MSI; (ii) capsid portal protein from Altivir_4_ACLF; (iii) major tail tube protein from Altivir_7_ACLF, and (iv) terminase protein from Altivir_8_HURL. For each phylogenetic tree, the following workflow was performed. The respective viral hallmark protein was used as BLASTp query to search for related proteins in the viral section of the NR BLAST database from NCBI (https://blast.ncbi.nlm.nih.gov/). If no results were found with BLASTp (capsid protein from Altivir_4_ACLF, major tail tube protein from Altivir_7_ACLF), then DELTA-BLAST was used to search for distantly related proteins. Only hits with an *e*-value smaller than 0.0005 were kept. The selected proteins, including the query, were aligned using the Constraint-based Multiple Alignment Tool (COBALT^104^), using the online tool (https://www.ncbi.nlm.nih.gov/tools/cobalt/re_cobalt.cgi). The COBALT alignment was imported in Geneious Prime 2020^90^ for refinement using MUSCLE v.3.8.425^105^ and end trimmed. The tree for the Altivir_2_MSI was constructed using FastTree v.2.1.12^106^ with the “Whelan and Goldman 20012” and “Optimize the Gamma20 likelihood” parameters. The trees for Altivir_4_ACLF, Altivir_7_ACLF and Altivir_8_HURL were calculated using the webserver phylogeny.fr. Here, the alignment was first curated with Gblocks^107^ (option “Do not allow many contiguous nonconserved positions”). The tree was reconstructed using PhyML^108^ with the substitution model “WAG for protein” and the approximate likelihood-ratio test “SH-like” for calculating the branch support statistics. All trees were exported as “.newick” format, visualized with FigTree v.1.4.3^109^, rooted at the midpoint, and further annotated in Inkscape v.1.0.2 (https://inkscape.org/).

### Relative abundance of viruses and hosts

Abundance of hosts and viruses was determined via read mapping against respective genomes using Bowtie2^110^ in sensitive mode followed by mismatch filtering for host genomes (2%, depending on read length). Abundances were normalized to the total number of base pairs (bp) sequenced of each sample scaling to the sample with lowest counts, which is herein referred to as relative abundance or normalized coverage. Rank abundance curves were built based on the abundance of scaffolds carrying ribosomal protein S3 (*rpS3*) gene sequences predicted in metagenomic assemblies after running prodigal (meta mode)^74^ and annotation^68^ against UniRef100^75^. For normalized rank abundance curves based on sequencing depth for the ecosystem MSI, we only considered *rpS3* gene sequences with a coverage > 2.4 after normalization as this value represents the lowest coverage of any assembled *rpS3* gene sequence in the MSI dataset.

### Design, synthesis, and chemical labeling of gene probes

To target Altivir_1_MSI, an 8.9 kb putative viral genome recovered from the MSI_BF_2012 metagenomic dataset, 11 dsDNA polynucleotide probes of 300 nucleotides length were designed using genePROBER (gene-prober.icbm.de/). In addition, a single 300 bp fragment of a *Metallosphaera* turreted icosahedral virus strain MTIV1 (NCBI accession no. MF443783.1) was designed as a negative control probe, because the *Metallosphaera* sp. virus was not detected in the metagenome of MSI_BF_2012. Sequences for all probes are given in Supplementary Tables 6 and 7.

All probes were chemically synthesized by IDT (Integrated DNA Technologies, San Jose, CA, USA) as gBlocks^®^ Gene Fragments (500 ng per polynucleotide), reconstituted in 5 mM Tris-HCl pH 8.0, 1 mM EDTA pH 8.0, and then labeled with the ULYSIS™ Alexa Fluor™ 594 nucleic acid labeling kit (Thermo Fisher Scientific, MA, USA)^111^. Two micrograms of either an equimolar mixture of the 11 Altivir_1_MSI polynucleotides or of the single negative control polynucleotide were used in a single labeling reaction and then purified using NucAway Spin Columns (Thermo Fisher Scientific, USA). Before being used as probes for virus-targeted direct-geneFISH (virusFISH), the labeled probes were measured spectrophotometrically using a NanoDrop™ (Thermo Fisher Scientific, USA). The calculated labeling efficiency was 9.16 and 16.6 dyes per base for Altivir_1_MSI probe and negative control probe, respectively.

### VirusFISH of MSI BFs

VirusFISH was performed according to the direct-geneFISH protocol^112^, with the modifications detailed further. In brief, Altiarchaeota BF, dehydrated in an ethanol series, was carefully placed in the middle of a press-to-seal silicone isolator (Sigma-Aldrich Chemie GmbH, Taufkirchen, Germany) mounted on Superfrost^®^ Plus slides (Electron Microscopy Sciences, Hatfield, USA), and subsequently air-dried. Because Altiarchaeota lack the typical archaeal S-layer as outer sheath^113^ and hence are more prone to membrane disintegration, no extra permeabilization step was required. Different formamide concentrations (20, 30, and 50%) were tested to exclude false positive hybridization signals. In the final assay, 20% of formamide was used in the hybridization buffer that contained 5x SSC buffer (saline sodium citrate, pH 7.0), 20% (w/v) dextran sulfate, 20 mM EDTA, 0.25 mg mL^−1^ sheared salmon sperm DNA, 0.25 mg mL^−1^ yeast RNA, 1x blocking reagent, 0.1% (v/v) sodium dodecyl sulfate, and nuclease-free water. The final NaCl concentration of 0.225 M in the washing buffer corresponded to the 20% formamide in the hybridization buffer. As rRNA probes, the dual-Atto488-labeled probes NON338 (5′-ACTCCTACGGGAGGCAGC-3′)^114^ and a SM1-Euryarchaeon-specific probe SMARCH714 (5′-GCCTTCGCCCAGATGGTC-3′)^115^ were used. A volume of 45 µL hybridization mixture was used, with a final gene probe concentration of 330 pg µL^−1^ (30 pg µL^−1^ for each polynucleotide) and rRNA probe final concentration of 1 pmol µL^−1^. In the hybridization chamber, 30 ml of formamide-water solution was added to keep a humid atmosphere. The denaturation and hybridization times were 30 min and 3 h, respectively. The post hybridization washing buffer contained 20 mM Tris-HCl (pH 8.0), 5 mM EDTA (pH 8.0), nuclease-free water, 0.01% SDS, and 0.225 M NaCl. A second washing step with 1x PBS (pH 7.4) for 20 min was performed. Then, the slides were transferred for 1 min into molecular grade water and quickly rinsed in absolute ethanol. For staining, we used 15 µL of a mixture of 4′,6-diamidin-2-phenylindole (4 µg mL^−1^) in SlowFade Gold Antifade Mounting medium (both from Thermo Fisher Scientific, Waltham, MA, USA). All solutions and buffers for virusFISH experiments were prepared with molecular grade water (Carl Roth, Karlsruhe, Germany). For the experiment with individual flocks, 17 out of 18 flocks were treated with the Altivir_1_MSI probe and one flock with the negative control probe.

The BF material was examined and imaged with an Axio Imager M2m epifluorescence microscope equipped with an AxioCam MRm and a Zen 2 Pro software (version 2.0.0.0) (Carl Zeiss Microscopy GmbH, Jena, Germany). Channel mode visualization was performed by using the 110×/1.3 oil objective EC-Plan NEOFLUAR (Carl Zeiss Microscopy GmbH) and three different filter sets from Carl Zeiss: 49 DAPI for visualizing Altiarchaeota cells, 64 HE mPlum for the detection of viral infections, and 09 for achieving 16S rRNA signals. Enumeration of cells and viral signals was performed manually. Viral signals were categorized into three major groups, i.e., viral adsorption on host cells, advanced infections, and viral bursts.

### Structured illumination microscopy

For structured illumination microscopy, the whole virusFISH protocol was carried out on a BF flock mounted on a cover slip (thickness no. 1.5H, Paul Marienfeld GmbH & Co. KG, Lauda-Königshofen, Germany). Staining was conducted without adding DAPI to the mounting medium. Samples were analyzed using an inverted epifluorescence microscope (Zeiss ELYRA PS.1) equipped with an α-Plan-Apochromat 100×/1.46 oil DIC M27 Elyra objective, F Set 77 He filter, and a Zen 2.3 SP1 FP3 black edition software (version 14.0.22.201), all obtained from the manufacturer Carl Zeiss Microscopy GmbH, Germany.

### Transmission electron microscopy

BF flocks from MSI were prefixed in glutaraldehyde (Carl Roth, Karlsruhe, Germany) to a final concentration of 2.5% (v/v) and physically fixed via high-pressure freezing followed by freeze substitution, which was carried out with 0.2% (w/v) osmium tetroxide, 0.25% (w/v) uranyl acetate, and 9.3% (v/v) water in acetone^116^. After embedding in Epon resin and polymerization for 72 h, the samples were ultrathin sectioned and poststained with 1% lead citrate for 2 min. Transmission electron microscopy was carried out on a Zeiss EM 912 (Zeiss, Oberkochen, Germany) with an integrated OMEGA-filter at 80 kV in the zero-loss mode. Imaging was done using a 2k × 2k pixel slow-scan CCD camera (TRS Tröndle Restlichtverstärkersysteme, Moorenweis, Germany) and an ImageSP software (version 1.2.9.77) (×64) (SysProg, Minsk, Belarus).

### Reporting summary

Further information on research design is available in the Nature Research Reporting Summary linked to this article.

## Supplementary information

Supplementary information

Description of Additional Supplementary Information

Supplementary Data 1

Supplementary Data 2

Supplementary Data 3

Reporting Summary

## Data Availability

Sequencing data generated and analyzed during this study are available at NCBI’s SRA and GenBank with accession codes as listed in Supplementary Table 1. Metagenomic reads are available from NCBI Bioprojects #PRJNA628506, Biosamples #SAMN14733005-07, Run #SRR11614986-88 (MSI_2018), Bioproject #PRJNA321556, Biosample #SAMN04999996-97, Run #SRR3546456-57 (HURL), Bioproject #PRJNA627655 (GA), Biosample #SAMN14680028-30, Run #SRR11600161-63 (GA), Bioproject #PRJNA340050, Biosample #SAMN05661201, Run #SRR4293692 (ACLF), as well as from ENA #PRJNA678866, Biosample #SAMEA2779769, and Run accession #ERR628383 (MSI_2012). Metagenomic assemblies can be found under the above-mentioned Bioprojects/samples for MSI_2018 and GA. Exceptions represented MSI_2012, which was deposited under Bioproject #PRJNA678866, Biosample #SAMN16815598, HURL assemblies under Bioproject #PRJNA730881, Biosample #SAMN04999996-7, and the ACLF assembly under Bioproject #PRJNA730879 and Biosample #SAMN05661201. Run accessions for these assemblies are JAEMOC000000000–JAEMOL000000000. *Ca*. Altiarchaeota genomes were deposited at GenBank under Bioproject #PRJNA628506, MAG Biosample #SAMN18220766, Run #JAGTWS000000000 (MSI_2018), Bioproject #PRJNA627655, MAG Biosamples #SAMN18220852-54, Run #JAGTWP000000000-JAGTWR000000000 (GA), Bioproject #PRJNA726854, MAG Biosample #SAMN18221259, Run #JAGWDR000000000 (ACLF), Bioproject #PRJNA726852, MAG Biosample #SAMN18220774-75, Run #JAGWDQ000000000 and #JAGWDP000000000 (HURL), and at ENA under #CCXY01000000 (MSI_2012). Viral genomes of *Ca*. Altiarchaeum virus Altivir_1 and 2 can be found under GenBank accessions #MW522970 and #MW522971, respectively. *Ca*. Altiarchaeum virus Altivir_3-8 genomes are available in the Third-Party Annotation Section of the DDBJ/ENA/GenBank databases under the accession numbers #BK059157-162. Viral genomes of *Ca*. Altiarchaeum virus Altivir_3-8 can also be accessed via the scaffold accession numbers as given in Supplementary Table 4. Viral genomes can also be found at https://github.com/ProbstLab/viromics/tree/master/viruses/Altivir. Microscopy data can be obtained from the corresponding author upon reasonable request.

## Code availability

Our tool Endmatcher for identification of circular sequences from metagenomes is available under https://github.com/ProbstLab/viromics/tree/master/Endmatcher.
