# Supplementary material for: Lytic archaeal viruses infect abundant primary producers in Earth’s crust
Source: Nat Commun. 2021 Jul 30;12:4642. doi: 10.1038/s41467-021-24803-4 (PMC8324899; doi:10.1038/s41467-021-24803-4)
Supplement: Supplementary file 1 — Supplementary information [file 41467_2021_24803_MOESM1_ESM.pdf]

Supplementary Information for:
Lytic archaeal viruses infect abundant primary producers in Earth's crust

Janina Rahlff[1*†], Victoria Turzynski[1*], Sarah P. Esser[1], Indra Monsees[1], Till L.V. Bornemann[1], Perla Abigail Figueroa-Gonzalez[1], Frederik Schulz[2], Tanja Woyke[2], Andreas Klingl[3], Cristina Moraru[4], and Alexander J. Probst[1#]

[1]University of Duisburg-Essen, Department of Chemistry, Environmental Microbiology and Biotechnology (EMB), Group for Aquatic Microbial Ecology, Universitätsstraße 5, 45141 Essen, Germany

[2]DOE Joint Genome Institute, 2800 Mitchell Drive, Walnut Creek, CA, 94598, USA

[3]Plant Development & Electron Microscopy, Biocenter LMU Munich, Großhaderner Str. 2-4, 82152 Planegg-Martinsried, Germany

[4]Institute for Chemistry and Biology of the Marine Environment (ICBM), Carl-von-Ossietzky-University Oldenburg, PO Box 2503, Carl-von-Ossietzky-Straße 9-11, 26111, Oldenburg, Germany

*authors contributed equally

[#]Corresponding author: alexander.probst@uni-due.de

†Present address: Centre for Ecology and Evolution in Microbial Model Systems (EEMiS), Department of Biology and Environmental Science, Linnaeus University, SE-39182, Kalmar, Sweden

Supplementary Table 1: List of accession numbers for metagenomic reads, assemblies and genomes deposited in public databases. ACLF=Alpena County Library Fountain, BF= biofilm, ENA=European Nucleotide Archive, GA=Geyser Andernach, HURL=Horonobe Underground Research Laboratory, MSI=Mühlbacher Schwefelquelle Isling, SRA=Sequence Read Archive, TPA= Third Party Annotation Section of the DDBJ/ENA/GenBank databases.

Metagenomes (raw reads)

Sample	Database	Bioproject accession	Biosample accession	Run accession
				ERR628383
MSI_2012_Biofilm	ENA	PRJEB6121	SAMEA2779769	ERR628383
MSI_2018_Biofilm	NCBI/SRA	PRJNA628506	SAMN14733005	SRR11614987
MSI_2018_0.1µm	NCBI/SRA	PRJNA628506	SAMN14733006	SRR11614986
MSI_2018_post0.1µm	NCBI/SRA	PRJNA628506	SAMN14733007	SRR11614988
HURL_250m	NCBI/SRA	PRJNA321556	SAMN04999997	SRR3546457
HURL_140m	NCBI/SRA	PRJNA321556	SAMN04999996	SRR3546456
GA_1_1	NCBI/SRA	PRJNA627655	SAMN14680028	SRR11600163
GA_1_2	NCBI/SRA	PRJNA627655	SAMN14680029	SRR11600162
GA_2_1	NCBI/SRA	PRJNA627655	SAMN14680030	SRR11600161
ACLF	NCBI/SRA	PRJNA340050	SAMN05661201	SRR4293692

Assemblies

Sample	Database	Bioproject accession	Biosample accession	Run accession	% Rel abund. Altiarchaeota*
MSI_2012_Biofilm	NCBI/GenBank	PRJNA678866	SAMN16815598	JAEMOL000000000	90.7
MSI_2018_Biofilm	NCBI/GenBank	PRJNA628506	SAMN14733005	JAEMOK000000000	97.6
MSI_2018_0.1µm	NCBI/GenBank	PRJNA628506	SAMN14733006	JAEMOI000000000	38.5

MSI_2018_post0.1µm	NCBI/GenBank	PRJNA628506	SAMN14733007		JAEMOJ000000000	74.9
HURL_250m	NCBI/GenBank	PRJNA730881	SAMN04999997		JAEMOG000000000	68.9[#]
HURL_140m	NCBI/GenBank	PRJNA730881	SAMN04999996		JAEMOH000000000	77.1[#]
GA_1_1	NCBI/GenBank	PRJNA627655	SAMN14680028		JAEMOC000000000	86.1
GA_1_2	NCBI/GenBank	PRJNA627655	SAMN14680029		JAEMOD000000000	87.1
GA_2_1	NCBI/GenBank	PRJNA627655	SAMN14680030		JAEMOE000000000	88.1
ACLF	NCBI/GenBank	PRJNA730879	SAMN05661201		JAEMOF000000000	52.0

Altiarchaeota Genomes

Sample	Database	Bioproject accession	Biosample (physical)	Biosample Genomes	WGS accession
MSI_2012_Biofilm	ENA	PRJEB6121	SAMEA2779768		CCXY01000000
MSI_2018_post0.1µm	NCBI/GenBank	PRJNA628506	SAMN14733007	SAMN18220766	JAGTWS000000000
HURL_250m	NCBI/GenBank	PRJNA726852	SAMN04999997	SAMN18220774	JAGWDP000000000
HURL_140m	NCBI/GenBank	PRJNA726852	SAMN04999996	SAMN18220775	JAGWDQ000000000
GA_1.1	NCBI/GenBank	PRJNA627655	SAMN14680028	SAMN18220852	JAGTWP000000000
GA_1.2	NCBI/GenBank	PRJNA627655	SAMN14680029	SAMN18220853	JAGTWQ000000000
GA_2.1	NCBI/GenBank	PRJNA627655	SAMN14680030	SAMN18220854	JAGTWR000000000
ACLF	NCBI/GenBank	PRJNA726854	SAMN05661201	SAMN18221259	JAGWDR000000000

Viral Genomes

Sample	Database		Accession
Altivir_1 to Altivir_8	Github	https://github.com/ProbstLab/viromics/tree/master/viruses/Altivir	
Altivir_1_MSI_BF_2012	NCBI/GenBank		MW522970.1
Altivir_2_MSI_BF_2012	NCBI/GenBank		MW522971.1
Altivir_3_ACLF	TPA		BK059157
Altivir_4_ACLF	TPA		BK059158

Altivir_5_ACLF	TPA	BK059161
Altivir_6_ACLF	TPA	BK059159
Altivir_7_ACLF	TPA	BK059160
Altivir_8_HURL	TPA	BK059162

*Percent relative abundance compared to other community members based on rpS3 sequence analysis.
[#] since the metaSPAdes assembly of the Altiarchaeota genome was extremely fragmented and the *rpS3* gene did not assemble we used a scaffold carrying the ribosomal proteins L30 and L15 for coverage estimation of the dominant Altiarchaeum.

Supplementary Table 2: Characteristics of potential *Ca.* Altiarchaeum viruses detected in subsurface ecosystems. Viruses were clustered based on genus level and intergenomic similarity (Figure 2, Supplementary Figure 4&5). Details on functional annotation of each protein of each virus using a variety of different tools can be found in Supplementary Data 3. ACLF=Alpena County Library Fountain, BF=biofilm, HURL=Horonobe Underground Research Laboratory, MSI=Mühlbacher Schwefelquelle Isling, ORF= open reading frame, VOGDB=Virus Orthologous Groups database.

Predicted viral clade	Sample	Coverage	Normalized coverage	Host:virus ratio	Genome length (kbps)	%GC	No. ORFs	Av. ORF length (bps)	Coding density (%)	No. of non-normalized spacer matches (associated repeat type)	Classification	Tools for identification	Functional annotations of proteins
Altivir_1_MSI[C]	MSI_>0.1µm_2018	545	179	2.3	8.9	35.0	13	526	76.6	61 (1)	Putative virus	not identified	Nuclease, Geranylgeranyl transferase type i beta subunit
	MSI_<0.1µm_2018	32	18	23.4	8.8	35.2	13	514	75.8	32 (1)		not identified	
	MSI_BF_2018	20	9	301.9	8.9	35.3	13	517	75.4	278 (1)		not identified	
	MSI_BF_2012	4561	767	3.0	8.9	35.2	14	499	78.4	108 (1)		VirSorter cat 3<br>circular	
Altivir_2_MSI[C]	MSI_>0.1µm_2018	456	150	2.8	17.4	24.0	35	421	84.7	0[P]	Putative virus	VirSorter cat 3<br>VOGDB	DNA methylase N-4/N-6, Transcriptional regulator, DNA Polymerase B
	MSI_BF_2018	39	18	150.2	22.7	24.7	43	455	86.2	29 (1)	Putative virus	VirSorter cat 3<br>VogDB	
	MSI_BF_2012	1048	176	13.1	20.8	24.6	39	450	84.8	7 (1)	Virus	VirSorter cat 3<br>VOGDB<br>circular	
Altivir_3_ACLF	ACLF	240	199	4.8	12.6	31.9	15	787	93.8	9 (1)<br>8 (2)	Putative virus	VirSorter cat 3	-
Altivir_4_ACLF[T]	ACLF	169	140	6.8	8.2	32.0	18	360	78.9	3 (1)<br>10 (2)	Putative virus	VirSorter cat 3<br>VOGDB	Capsid portal protein, Transposase

Altivir_5_ACLF[C]	ACLF	15	13	75.0	5.1	27.8	16	245	76.2	5 (1)<br>2 (2)	Putative virus	VirSorter cat 3<br>circular	-
Altivir_6_ACLF	ACLF	13	11	85.6	4.6	35.0	8	509	88.8	18 (1)<br>9 (2)	Putative virus	VirSorter cat 3	-
Altivir_7_ACLF	ACLF	65	54	17.8	3.8	29.1	9	323	75.8	1 (1)<br>0 (2)	Putative virus	VirSorter cat 3<br>VOGDB	Major tail tube protein, DNA gyrase/topoisomerase, RNA ligase
Altivir_8_HURL[H,C]	HURL_250m	1843	913	1.8	22.6	35.6	36	502	79.9	13 (1)<br>59 (2)	Virus	VirSorter cat 2<br>VOGDB<br>circular	Terminase, Structural proteins, Major capsid protein, Prohead core protein serine protease, Peptidase M3B, Minichromosome maintainance protein, PAPS reductase, Portal protein

[C]circular genome
[H]carries hallmark genes
[T]potential transposon

[P]In this sample the virus was only recovered as a partial sequence and recruited no spacer matches from the same sample. However, the more complete genome from 2012 did have matching protospacers as shown in Figure 4.
Note: No Altiarchaeota viruses were detected in samples from HURL (140 m) and GA

Supplementary Table 3: Sampling information for biofilms (BF) collected from the Mühlbacher Schwefelquelle, Isling, Germany (MSI); FISH=fluorescence *in situ* hybridization.

Sample	Time of sampling	Analyses	Figures
BF samples for virusFISH	17 - 19.01.2019	Fluorescence microscopy	Fig. 3 (A-C), Fig. 5, Supplementary Fig. 11, Supplementary Fig. 12, Supplementary Fig. 16, Supplementary Fig. 17, Supplementary Fig. 18
BF samples for transmission electron microscopy	17 - 19.10.2018	Transmission electron microscopy	Fig. 3 (D)
BF, >0.1µm and <0.1µm fraction	15 - 17.10.2018	Metagenomics	Fig. 1, 2, 4
BF	11.2012 - 04.2012	Metagenomics	Fig. 1, 2, 4

Supplementary Table 4: Assignment of viral scaffolds to assemblies. ACLF=Alpena County Library Fountain, BF=biofilm, HURL=Horonobe Underground Research Laboratory, MSI=Mühlbacher Schwefelquelle Isling.

Ca. Altiarchaeum virus	Assembly Sample	Scaffold accession #	Scaffold_name (unmodified) corresponding to virus	Scaffold has been modified?*
Altivir_1_MSI_BF_2012	MSI_2012_Biofilm	JAEMOL010001844.1	IMS_noSM1map_1844_length_8923_cov_1978.89	yes, circular scaffold was opened at position 8902 to avoid split genes[V]
Altivir_1_MSI_BF_2018	MSI_2018_Biofilm	JAEMOK010000586.1	IMS_biofilm2018_586_length_8910_cov_12.0567	yes, reverse complement of unmodified and opened at 2094 to align with Altivir_1_MSI_BF_2012
Altivir_1_MSI_>0.1µm_2018	MSI_2018_0.1µm	JAEMOI010003611.1	IMS_filtrate2018_3611_length_8924_cov_331.471	yes, was opened at 4335 to align with Altivir_1_MSI_BF_2012
Altivir_1_MSI_<0.1µm_2018	MSI_2018_post0.1µm	JAEMOJ010000621.1	IMS_vir2018_vir2018_621_length_8816_cov_19.1948	yes, reverse complement of unmodified and opened at 2103 to align with Altivir_1_MSI_BF_2012
Altivir_2_MSI_BF_2012	MSI_2012_Biofilm	JAEMOL010000523.1	IMS_noSM1map_523_length_20787_cov_478.723	yes, scaffold was opened at position 820 to avoid split genes
Altivir_2_MSI_BF_2018	MSI_2018_Biofilm	JAEMOK010000143.1	IMS_biofilm2018_143_length_22687_cov_24.831	yes, was opened at 2251 to align with Altivir_2_MSI_BF_2012
Altivir_2_MSI_>0.1µm_2018	MSI_2018_0.1µm	JAEMOI010000767.1	IMS_filtrate2018_767_length_17396_cov_288.247	yes, reverse complement
Altivir_3_ACLF	ACLF	JAEMOF010000700.1	Sulflake_Alpenaf__700_length_12584_cov_115.502	no
Altivir_4_ACLF	ACLF	JAEMOF010001180.1	Sulflake_Alpenaf__1180_length_8205_cov_84.5962	no
Altivir_5_ACLF	ACLF	JAEMOF010001897.1	Sulflake_Alpenaf__1897_length_5148_cov_10.2435	yes, circular scaffold was opened at position 5149 to avoid split genes
Altivir_6_ACLF	ACLF	JAEMOF010002130.1	Sulflake_Alpenaf__2130_length_4588_cov_5.55703	no
Altivir_7_ACLF	ACLF	JAEMOF010002504.1	Sulflake_Alpenaf__2504_length_3832_cov_28.6254	no
Altivir_8_HURL	HURL_250m	JAEMOG010001425.1	BJP_V-250m-2014_1425_length_22641_cov_1164.08	yes, circular scaffold was opened at position 4821 to avoid split genes[V]

*Remark: Scaffolds had to be re-opened or changed in their orientation for alignments or due to circularity. All final scaffolds are also available at https://github.com/ProbstLab/viromics/tree/master/viruses/Altivir

[v]Scaffold has been finally taken from VirSorter output, which produces slightly shorter scaffolds for circular viral genomes (eliminates overlaps)

Supplementary Table 5: Chemical composition of spring water from the Mühlbacher Schwefelquelle (MSI), taken from [1].

Chemical compound	mg L^{-1}
SO_4^{2-}	16
$S_2O_3^{2-}$	14
SO_3^-	<1
NO_3^-	<1
NO_2^-	<0.02
NH_4^+	0.33
Na^+	21
Cl^-	22
PO_4^{3-}	<0.05
Ca^{2+}	79
Mg^{2+}	27
K^+	6.1
Mn	0.081
Ni	<0.01
As	<0.002
Al	<0.05
Sb	<0.003
B	0.13
Cu	<0.005
Se	<0.003
Zn	<0.05
Fe	0.021
CO_2, dissolved	32
O_2	0.13
H_2S	0.5

Supplementary Table 6: Sequence of the circular viral genome Altivir_1_MSI_BF_2012. The regions corresponding to polynucleotide binding sites are represented in different colors.

via:<br>>VIRSorter_NODE_1844_length_8923_cov_1978_89-circular-cat_3_modified<br><br>11 polynucleotide probes (each 300 bp) from IDT (Leuven, Belgium):<br><br>Altivir_1_MSI_probe_1<br>Altivir_1_MSI_probe_2<br>Altivir_1_MSI_probe_3<br>Altivir_1_MSI_probe_4<br>Altivir_1_MSI_probe_5<br>Altivir_1_MSI_probe_6<br>Altivir_1_MSI_probe_7<br>Altivir_1_MSI_probe_8<br>Altivir_1_MSI_probe_9<br>Altivir_1_MSI_probe_10<br>Altivir_1_MSI_probe_11	CCTGCCCAGATACTACATCTCAAACATTCCTCCAGGCATATCTTTGTGCCATACAATATCAA<br>AAAACAGGCGTAAAAGACGAACACGACTTTGAAATTTATTCCAGTTTTGACATTGAAGATCA<br>AAACGAATATGTTTATATTTCATCTGAAAGAGAAAAGAGTGACTTGTTCGAACAACAAAACG<br>CAAACTGGTATTTGATTGAAAGAGATTTACTTGGAAAAATCACGTACATCGACACAGAAACC<br>GGAGATGATTTTTCATTCAAAGAAATTGAGTCTGGGAAGCGAAAAATAGTCGAAGCGGGTGC<br>TGGAAGCATCTTCAAACTTCTCGGAAAACTAAAAACAAAATTTGGTAATTTTTTGCTCGCCA<br>ACGCAAGCCAAAAATCACTGTGGATATTAAAAAAAGAAAAATCATCAAGCTGGACAAAAAAA<br>CGGGAGCGTTCGTAATTGCCACAGGTATTTCTTCCACAACACTCATTACATCCCTCTTAAGC<br>CAAACCTCATCAACACTTCTCGCAAAAGTTCGCCTTCTCATTGAAATGCAGGGAATAGCACT<br>TTTCACGGATTTCATTGCTGAAGAAGCAGTTCAAAGTGCAGGAATGGGTGTCTTCATAGCGA<br>AAGGTCGCGACACAAAAACACTCGAACATGCTATCTCTACATACAAAAATATATATTCTCTG<br>GCTGAAAATGTACATCAATACGCACAGTGGAATATAGCGAAAAATGCCTTTGATGCCTTTTT<br>CCTCTCCGCAAAAGAAAACATAGTGATATATGAGGCACTCCTTGAAAATGCGAAAGAAACAG<br>CTGCACTTCGCGGAGAAACAGAGGACGCGGGAAAAGTCACTATATATTCTACCGAAAAATAT<br>GCAATATACCTCAACGGAATTAATACAAAAAAATACTCTCCGTCAGTCATACGAATTCCACC<br>AGGAACGCATACGATAGACCTGAAAATGAAAGACAAAATCACATATACAGAAACATTTTCAA<br>TCGAAAAATATGGCATCAAAAACATCGGGTCTGTTTCAAAAAAAATTGAACTACAAAACACA<br>ACAAAATGTCTTGATGTTGCCAACGGAACAACGATAATTCTCAACGATTTTCAACAAATCGT<br>ATTGGCAGGCGTAAAACAAAAAATTTTAACAGCAGCAGCTGAAAAAGCATCAAAAAACTGGC<br>TTCGTAAAACATGCGAAACAAGGAATATTCGCATCGAAACCGATACAGAAACTCATAACGAA<br>CATGGACAATTGTTAGGCTGGATTTTTCTTGGGGATCTAAACGTAAACGTTGAATCCGTAAA<br>ACGCGGATTTACAATAAGTAACAAAACAACAAAAAAATATGAAGATGAAATAAAAGAAGCGG<br>AAAAATTTGCCGAAAAATAATAAATTACAACAAAAAAATGTTCGGAAGAGCAAATTTTTTGCC<br>AAAACACCAGAACAAAAAAGACCTACTATGGAAAGACATAGATGACTTTGTCAATTTTTATT<br>TCTTGAATCAAAAAATTTCAAAATCATACGAATTTGAACTTTACAACCCACGATGGTTTAAC<br>TCCACAAAAGCTTGGCAAATTTCATGCGCAAAGAAAATAGACCTGGTTGTAAATCGTAACGA<br>TGAAATTTATATTTTTGAAACCGACCCCCAAATTCTCGATGAAACAATCGGGCAACTTCTCA<br>CCTATCGTTTTTGGTATGAAAAGCAGGAACAAAAAAAAGTGAAAAAAATGATAGCACTATGT<br>GGGGAAATCCAAGAGCAAACTGCCGAAGTAGCACTTCTGTATGGAATAGAAGTCTGGAAAAC<br>AAATTTTCCTTCTGGATATACTCAGTATGTTTCTATGTACTAAATATGTTTCTATCTATTGA<br>ATTCATCGAAATTAAATTTTTTCTTGCCTAAATTACCCTCAAAAGTATGGGGAGCGACAAAT<br>AGAAGATTGTCCTCTTGGCCAATCTTCTAGGGATATTAAATTTTCCCCCGCCTAAATTGTTC<br>TTAAAAGTACGGGGAGCGACCGGGGGGGCTCAAACAAAAAAAAAAATGTGAATTTTGATTTTT<br>TTTTTGTTTGCTCGGAGCCTGGCGGGCTCTTGCCCGCCCGGCTCCGAGGTGGAGTCTGGTTT<br>GGGGGCAAGCGGCTTCGCCGAAAATTCTTTGGAATTTTCCCCCAAACCATCCTCCACCTCTG<br>AGCCTATCGGCTCCGAGACGCCCCCCAGCCTATCAAAACATCAGAAAAATTCTTGGGCTACG<br>GGTATGAGCCCTACGCCCAAGAATTTTTCAGAGGTTTTGATAGGCTATATATCTTCTCATTG<br>TTATAAAAATTCTCCACAAAGTCTTCCTAAATTTTCCTAAATTTTCCTAAATTTTCCTAAAT<br>TTTTCCCTCCCTTGCTCCCTCATAAGACCGCTACCATCAAACAAACTTAAGGAATACTATAT<br>ATGCCACCTCATAAGTATCTTTTTTCTGCGAAGAAAAAAGATACTTAAAAGGGCTCTGGCAT<br>ATATATCCAACCATAAGTTCGTTTGAAGGTGCGAGCTTATGAGGGGCAAGGGGGGACGAATA<br>CAAATTTTCCTAAATTTTTCCGGAAGGTGAACATCGGAGGGGAGTTAAGTCCGAATATAAAT<br>CTCCTACTTGCGAGACAAACGCCAAATTTTCGGAATGGCGAACTCGGAAAGGGGAGTTAAGC<br>TAAATTTCCCACTGCGAGACAAACGCCAAATTTTCGGAATGGCGAACTCGGAAAGGGGAGTT<br>AAGCTAAATTTCCCACTGCGAGACAAACGCCAAATTTTAAAATTTCAAAATAATAAACAAAC<br>GACCATGAAAAATATAAATCCCACAAATGTGGTGATAGGACGCAAAAAAAAATTTGAAGAAG<br>AAATGAAAGAAAAAATAGAAGAATATGTGAGGAACCATCAAGAAGAAATAGTAGAGAAAGAA<br>AAAGAGAACATGAATCCGCCTGGAACAGCAGTCGTCTGGCAAGAGTCGGGTGGGATATTTCC<br>CTCTCGCCGGAAAAATCCGAGGGCGATATGACTTGTGATTGCTTCTTCGTTGAGATTAACAA<br>GAAAACACAAACACACACAATATTTTTTATTTTTTATTTTTTAAAAGTTTTGCTCATTCCCG<br>TATTTTTAAACCTTTCACCTTTATTCAAAGGTAAATCTTTTAAATATGGTTATGTGTGCTAA<br>CTTCTGCGGTTTAAACTTTTTAACTAACACACACAAAAAAAAAGTTTATAAAAGAAAGGTAG<br>CCAAAGAAAATTATCAAAAGCAACAAAGTGGTATGTCATCGCTTAACAATTATGAAAGCGAG<br>GTTAAGTATTAATTAATTTTAAACTTTTTATCTTTATTCTTTGTTAGAAAAATTTAATTATT<br>ATTATTATTAAAAAAAATATAAAAAAAAATAAATAAAAAACAATGGAAAATATTTTTGAAAC<br>CGTGATGAAGGAACCAATAGAAATTAAAAAAAAACGCGAACAAATAGCAAGAATAAAATATC<br>TCAAAGACCGACATGGCGTCATTTCCCTATGGCGCGAAGAACCAGTCCAGATAAATCTTCGG<br>GGATATCTCCTGACTATCAGGCGTATCGAAGGTGAAACAGGAACGACAATTTCTGATGTCGC<br>GAAAGAAACGGGAAAAAACATTAAAACCGTCACGAATATTTTTAGAAATCTTGAAAAAATTG<br>AAGTGATAAAAATTTCCGAACATGCACATTTCTCTCGAAGAAAAAAAAATAAGAAAAACATA

	AATTTTTTCAAATCAAAATTCAAATTGACAACATACATTGAGGAATATTACTCGAATCATAG<br>TTTTTTCCGAAATCAATATACGTATAGCGATCTCAAACCTGCTGTGATTGAAGCAAAAGAAA<br>ATGAAAGAGTATCAAAACGCATGGATCTTGAGGAATCTCGTTATTTATGCTCAAAATCTATT<br>GTGCAGTTTGACCGTGTCTCGCCGCCCTCGCCGGAAAAATCCGGAGGTGATGTTTTACCCTC<br>ACGGAAAAATCCGGAGGTGATGTTTTACCCTCACCGGAAAAATCCGGAGGTGATGTCTCACC<br>CTCACCGGAAAAATCCGGAGGTGATGTCTCACCCTCACCGGAAAAATCTCACTGGCGGGATT<br>TGGATGAGGTATTTCCATCTCGTAAGAAAAATCCGAAGGCGTTATGACCTTCACCAGAAAAA<br>TCCGAAGACATTTGAATATGACGAATATAAAATATAGTTTTTTAGGTAACCTTTTTTCCCA<br>AAAAAGGTTTTATTTAATACAAGAAGTGCACAATAAATACAGTTTTTTCAAACTCTCCTTGT<br>TAAAAGTTTAATCATACACGATTAAACCTTTGACAAGGAGCATTTTTAAAAACCACCTTCAA<br>ACGGGGGGGTTACTTCTTGTTAAGGCATTACAGGAATTTTTTCCTCGCCAAAAAAATTTCCG<br>GACGCAAAATGAAATTTTTCCGGACGCAAGGTGAATCTATATGTATTTTTCTTAAATTTTTT<br>TTCAAGTAAAAAATATAAAATGACGAGGAATTGACACTCAACCCTGGTGTGGTTTGAGGGTG<br>AAGGGGGGTAAAATATAGGTTTTATGCCATTTTATTATTTATTTAATATCAAAATTTAATTA<br>TTTATTTAATATCAAAATTTAATTATTTATTTAATTATTTATTTAATATCCCTATTTTTTAG<br>GGATATTTTTTTTTTAAATCGTACTATGTAGTATAAAAAGATACATAAAAGATGAAAATAAA<br>ATTCAGAAACAACGGAAACAAAATATCAAATTATGCAGATATAGGAACCATATTGACAATAG<br>CAGCCAGCACGTTTTTTTCATCTTGTTCCCCACAAAATATTTTTGGCTGCTACATAGCCTTC<br>ACAATTGTTATTTTTCTAATAATAATAAAATATATTTCAGTAAAAAAAACATAAAAACACAA<br>AATGACAAAATCAGACTGGATGACCGCCACAGAAATAGAAAACGCCCTCAAAACAGGAATAA<br>AAAATGCAAAAAATGCATTCGAAAATGGGATAGATAATTGTAGGCGAGACCCCATAAAAACC<br>GCAATTGCAGCAATAACATCGGGAAAATGGGAAAAAAATTTCAAAGATTCCTCAGAAAAATG<br>GGAAAAAAATCTTTCAAAAATTTCTCTTGAAGACTGGAAGAAAGCCACAATCGCTGGGGCAT<br>CCAGATATGCTGACAACGCAGCCGACATAGGATCCGAAGAATGGGGAAAATATTACACTAGT<br>GCAAAATCAACTATCGAAGCAGCATCGTCAGAATTAATCGCAAGCAAACAAGAAAAAGCAGA<br>TTTTATCAAATTTTACGAGGCAATGTCCAAATTAAAAAACATAGATTAAAAATAAAGGCCCT<br>TTTTTTTGAAATTTCATAGCAACATCACGATATTAAATATAAAAGCCAAAATAAATTTAACA<br>ATAAAAAAAATAAAATGGCAATAAAACCAGAGGATGCAATAAAAAAGTATAAAAAATAATATA<br>GACCAAACAAAATACGAAAAAGGAGTTAATGCGGTCAAAACAAGCGTAACCGCAGCCGCGAT<br>AAAGCAGAAAGAAAGTTTCATCAAAAACACTCTCGCAGGTAAAGACCGCCTCATAAAAAATT<br>TGGGCAAAGTTTCAACTGATGTATGGAAAACCAAAACAAAAAGAAGTTTTTCAAAACTCGAA<br>GAAAAAGTTATTCTTGCGATTGACTCGGGAAAATGGAACGCAGCCAAAGTTCTCGCTGCGGG<br>ACAATCCGCGCACGCAACAGCGAAAAATATGAAAAAAGGATCATATTCTGATTCCTATGATC<br>GATATCTCGCATCACAAAACGCAATTAAAACCGCATGGAAATAATAAAAAAAATAAATATCA<br>AAAACACAAAATGACAATAATATCATCAAACAACACCAAATCTATTAATGGGACCGAACTCG<br>TTCTCGAAGCCGATATAGATAAAAGCATTCTCGTGAAAGGAGTGCACGTGCAAGGCTCAGTA<br>AACGGAAGGGCAACAGCCTACATTGGAGGCGTCACTGTGGGATACTTTCGAACAAACTCAAC<br>AATTCTCGGAAACCACCTTGAATACCTACGAAGAAATAATAAAAAAGAAATAAATGTACTAA<br>ATTTCCTTTTTCAAAACAATGTTTTTAAGGGCTACCCTCTCGCACCCGGTAATAAATTCACG<br>ATAAAAAATTCTTCAGCAGTAAATATGTCGGTAATCTATGATATTTACACAGAAGGCGACCA<br>AACAGAACTGATGGAAAACGGAAAAAACGGAAAAAGTTTAACATATCTTCACTACGGTCAAA<br>CCCCAAATTTCATCAACACTACTGGTGACACCCTCATATCTAAATCAAACAATCCAATTGAA<br>TTCTCGGGTTTTCCATTCACCTCAACCGTACCGCCAAAAAGAAAAATAGAGGTCCTCGGAAT<br>AGCGTTTTCATCTCGAGGTGCAGACGACGGAATCGCCGCAGCAAATTACATCAAAACCACAT<br>ATCTTAAGGTTATGAAAAACAGAGAAGTTCTTTTCGACGAAGAAAAAAAAGGTTTCATAGCG<br>ACAGGATCTCAAGTTTTAACCGCAAATACATTCGACGCCGAAAATGGACTTGACATAGGGGG<br>AGAAGGACATCTACGTATCAAAAGAATATTCTTTTATTTGACGAAAAAATTGTATGTGTCG<br>CAGGCGAAGAACTAAATTTTATCTGGTCAACAACTGTCGCTGCTATACCTGGAAGGTTTGAA<br>CCAGAAGAGACAGAAATTATGGTCATCTTAAGAGAAACAAAAGAATAAAAAATTTATAAAAA<br>ATAAATAAAATGACAATTTTATACAAAACTTTTAGTATTGTTTTAACCGCAGACAACCAAAA<br>ACATAACGCGACATCAGTCCTTACATGTCCAAAAGGAAAAAAAATACATTTTTCCTTTCTCG<br>GAATCGCACCATACATCACTGAAAACTGCCACATTCAAGTGTTCAAAGACCAAGAACAACTC<br>ACCGGAGAAGGAATATACCACGCTATTATCGACGATTACACTCACCGGCTTGATTTTGATGT<br>TGATCTCAACGAAGGAGAAGCAATCACAATCAAGGGATGGAGTATGACAGCATACACGGTGG<br>AAGGCGTTTATGCGTATGAAGAATTAATTTAAAAAAATTCAAATGTACAAATATATATGTTT<br>TGAAAACACACTACTAAAAACAGCTAATTTTAAAACATTATGTGAAATTGAAAAATCATTCG<br>TGGTTGGAGCGTCGATTAAATTTTGGGAAAACCCCTCTTTTTTTCCCTACGACGCGGTAATA<br>TCTATCTCAGACATAGGTGCTACAAAAAAATATATACTTGATATATCTTATCCGCATATGTT<br>CATTGCGAATTCAGAAATGTCTTACTTTGGCAAGTGGTCAAATGACACATTCATAGAATTGA<br>ACCACCAAATAGATATTGATCACAAACTATTGTTTGAAATTTTGGTCACCAACGATCTTCAA<br>GTTCAAGGATACGTTTTAATCGAGACATAAAAAATGAAGTTTTTAAACCTATATAAAAAAAT<br>AAGGAAAAAAACAACCATAAGACAAAATCAAACCCCAAAAACCACCGTACCTGAAACATTCG<br>AAAAAATATTTCTCGGGATATTTGAAAATTTTGAAAAAATAGGGTGGTCTACAACACATTTG<br>TATCTCTCAAACATTATTAAACGCACTTTTTCGTATATCCTATACCCAACACTCGACGGAA

	AAACGCAGGATTTTTCAAAGAAAAAACAGCTGGTGATATCACAACCCTCCAAACCCAGCCTG<br>GAGGAACGAAATTCTTTCTTGACAACAACCTCGTCCGTAGGACGAAAAGAATAGGTATTTCC<br>GCGTATAATATGTCAGACCTAGTAACATGCGGAGTGCATATCAGAGTCCAGGGACCATTCAT<br>CCAATCACCCATAAATGAAGTAAATCTATTCTACTTCACTGTTGCCCCGGTGACGCTCAAAA<br>CATTTGAATTTGATATTGCGTCGTTTGATGTATATCTTATTTTCGAAGCTGTTGGACCCACA<br>GGAGAATCAATAATTTTCAAAACTTATTATTTACCAATATAAAAATGGCTGATTATTTCGAA<br>TTCACCGTAAATGTCAAAGATACAGACAAAAATAATATTGAGGGTGCAAAAGTATCCATTCA<br>AGACATCACAGAAGAAACAACATCGGAAGGATATACAGACGAAAACGGAAATATTCTGTTTT<br>ACGTTCTTGCAAACAACCATATTTCCATAGAAAATGTAACATATGATCTGTATGAAATTAAA<br>TCAGGTCTCGTTTCAATACCGAAAATTACAGAAAATCTAACAGCTAAAATTGTCCTAAAAAA<br>AATAATAATAAAATATAATCTAAGTTTTACAAGTACAACTATGGAAAAAGCCTAATAAAATG<br>ACCCAAGAAAGAGCATACTGCGGGAACTGCATGAAAAAAGTAGAAATCAAAGATGGAAAAAT<br>TACAATCATTAATAAAAAAAAAGTTCTACAAGGCAAATGTCCATTTTGTGACTCCCTCTTAA<br>TAAAAAAAATATAAAAATGGCACTCGAACTTACAAAGGGTGATACCTTAAAAATAAAAATAG<br>GTGTAAAAAACGAATCTGATGGATTTACAGGAACGTACAAAATAAGTGTTGTGATCATGGAT<br>TCTAAGAATACGAAATACTACTTTAGCTACCATACTGACGGAAGCACTTTTTCAACCACTCA<br>ACAATCAATAACCGAAACACTGACGAAAGGCACAGAGTATTCTCTTTCATATTCCGCTGTCA<br>TACCTGATTACGAGGATGGCACAGTCACCGCAAATATAGGAATATACGGAGAAGAGGGGATA<br>ACAGAAATCATCGGAACAGGATGGTTTGACGCATATACTTATAGCAAAAAAAAAATGAATTT<br>GAGCTCGCTATCGGTGACCCTCGAGAAAGGCTAAC

Supplementary Table 7: Sequence of the non-matching *Metallosphaera* sp. virus probe. The region colored in grey refers to the polynucleotide used.

Metallosphaera virus (unclassified archaeal virus, 9.780 bp linear DNA), NCBI Accession no. MF443783.1<br><br>1 Polynucleotide probe (300 bp) from IDT (Leuven, Belgium):<br><br>*Metallosphaera* vir_nonprobe	CCCCCCCCCCCCATGCTGAAAGGGAGGGCGAGACCTAGCCTACCCCCCCCAGAGCAGGGG<br>GTTTAAAAGCCACGTCAGTTTGACGGATCACCACGCAAGTTGAACGCGGGGAGGGTTTAA<br>ATACCCCCCCTAGCATGAAAGCTTATTAACCCCAGGGGTTTTTGTCCTAATGCGAATGAG<br>CAAATTACAGCCCATAATACGCCCTGCCCCGCCCTGCAAGTACACTTTACGACACAAACC<br>GAAAGTAAACCTCAGAGTGAAGGGTTCTCTCCAACAGGGCAAAAAGCATTTTAACCCCCC<br>CATCACGACCTTCCGCAAAATAATTGGTCAGTCGTGGCTAAATTAAGTGAAGACTTTGTT<br>TAGGTAGTTTCGGTGGAGGGTGTGGCTAATAAGGGACAGTAAAGGTCACCATGTCGTCAA<br>ACCAGTCTAACCCAAAAAAGGAGGTCTCCCTGGAGGAGTTTTTTGAGGCCGTGAAGGACG<br>AGGTGATCCCCCTGAGAATGATAGCGGGGGATTACGTAGTCCTAGAGGGCGTCCACATGG<br>AGAACCTCAGGGACGGAAAGAACGGCAAGATAGTTGATGTGGTGGGCAGGATCGTCGTCA<br>CGAACATGGGCCTACCTGAGGGCCAGAAAGTCAGGTTCACGTTAGGCCTCTCGAACGCCA<br>TGGAAGTCGCCCATCATATAAAGGACGGCATAAAGTACACCATGATCAAGAAGGGGGAGA<br>TGAGGATAGTTGTTAATGGGTTTAGCGAGATCCCCAAGAGACTCTAGAGACGCTGAGGTA<br>TACCTAGTCAGGCCCCTGAAGGGCGTTTTTTCCCTCCAAGACCACGCTTTTCTCGTCACG<br>GTACGTGGCGTCTTCCCACGGAAGGGCGAGTTCACTCGAAAGAGCCACTGGGGCCTCATG<br>GTGGACGTGGACGTGGACGGCCTGTCCCTAAGGTTTAGGTCTAAACTCTACTTCACGTGG<br>CAAGTGGAGATGGGCGGTAGGCCGTGGGCTTTCGTGCCCGTGTACGGCTGTGCCGATATA<br>ATCCAGGAGTACGGATACCAGAGAGGTGTGGCCATATGTCAAAAATTGAAGTTGAGCAGG<br>TAGTCAAGTACATATTGGACCATCCTAGTACCTTCCTATGGTACAGGTGGTACCTGAGAC<br>AGAGGCACCACTTACCGTTCTTCTCGCTCACGTACAGTGAGGACTTACGACAGAAGGGGG<br>TGCCAGTGGACATGACCAGGGATAAGGAGGTAGTCGTGTTGGTGCCTATATACACCCCAT<br>ACGGCGTAGGCGGTGCTGAGGTCTTGTTGTCTACCCCAGATAAGGTTATGTGGAGTGACG<br>ACGACTTCGCCTTCTTCGGCAACTGGAAGTTTTACGGGAAGTTCGCGTGGATGGTCTCGC<br>TGAACCGAAAACACCCCCTGAGGCGGTACGCCCTGAACGAGATGAGGAAGGAGGCGAGGA<br>TGTATGCAGGCAAGTCCTGACATGGGGGCATTAACCCGCGCAGGGTATAGAGTGACGGCC<br>CGTTGGACGTGGAACGACGTCACTAGGGCCATGTATTGTTTTGAGACATCTAGGACAGCT<br>AACGACTTCGTGGGGTGCGTCAAAAAGCGTAGGACTGACGCCCTCGCTGTCTACCGATAC<br>CTACGAGACGTCCAGTCCCATCAGGGACTGATATATGAGGTACTCCCGCCCGCATTCCAG<br>GGCAACCCAGAGGAAGAGGAGGGGGAGGGGGAAGAGACTTGTATCACCTTCGTGGTCTAC<br>GACACGGGGTACTTCTTCCTCTGGGTAGACCCTAACCAGATCCTTCAAGACGCCTTAGAC<br>GCTAACGCGACCATGAGGATCAAGTACCCTGGGGCCTACCGTGTCATCACCACGCCGTAT<br>GACCTAGATTTCTCTCAAATAGCGTGGAGGCCCATAGTGCCGTCGGGCGAGGACTACGTG<br>ATATGTAGTAACATGGACCGTGTGGACTTCAGTGACGCGATAGACAGGTACGTCAGACAG<br>CTGAAGGCGATAATGGGGGCGATATCATGATCACGTTAGACCACGATGTACCATATGACG<br>GGTATAGACATTCGGCGAAAGGGAAACTCGTCGTTGAGTTTTGCAAGTCGTTCGAACTGA<br>GGTGTTGGTATAGGCGTTCGTCTACTGGGAACGTCCACGTGGCTATCGACGTGGACGTGG<br>ACTTCCTCAGAGAGTTGGAAATCAGGGCCGTCTTACTCGACGACCCCATGAGGATATTGA<br>ACGACTTGCGTCGGCACGCATTCCACATGCCTACTGGACGGCTCTGGGACGTGAAGGTGG<br>ATAGGGAAGGCAAAAGGCAGGCGGCCAGTGGGAGGTCTTGTTCTCCCCTGAGTAGGGTT

	TTTTACTCCACATATCCTACTTGTATCGTATGAGTCAAAGTCAGCCTACTCAGGAACAAA<br>CCCCCCCACAAGAGGAGGGGAAGGACGAGAGCCAGTGGACGGAATGTGAAGTCTGTGGGA<br>AGAAAGTGCGGATGAACATGTACAAACGTCATATGTCTGCGGTGCATGGGTTCATTACGA<br>AAGAGGAGTACCAGAGGCTCAAAGAGGAGGTGGAGAAGACTATACAATCTACGATACACA<br>GTGCACTCCAGGAGCACTGGAAGGGGCACTACAAAAGCTTAAGCGAACTTCTTGAGGAAG<br>GGGAAAAACATGCCGAGACGTGTCCTACATGCCAAAAGGAGTTGGAGGAGTGGCTCAAGA<br>AAAAACGGAACGAGAAATACAAACCCGCTAATAGGAAGTGGCTCTAAATGACCAAGAAGA<br>AGAAGGAGGAACAGGAGGCCCCTAAGGAGGAGGGGCAGGCCACTCCCGAAACCCCAAGTC<br>CTGAAACCCCTCAGGAGCCTCAGAAGGAAGACCCCGTTCAGGTCATCATGAGGCTGACGA<br>CCGTAATACAGGAACAGCAGAAACGTATAGACCAGTTGGAAGCAAACCTCAACAGCGTGA<br>TGGAATTCCTGAAGGCACAATCCCAGGCACCCCAAGGTCAGGGAGGAGGAGGGGGTCTAG<br>TGGAGGCACTAGTACCCCTCTTAGGGAAGTTGATGGAGCCTTCGAAAGACCCCCTACGGG<br>ATATCGCCCTAGAACTAATGATCGACAGCATGAAATCGAACGTTGAGCTCTCGAAGGCCA<br>TAACACAGAAGATCATCCAAGGGGTGCAAGACCGAGTCGCGAGGAACGTCGTGGAGACTG<br>TCGCCACGGGGGTGATTACACACGAATGAGCGTATTAGAATTATCAACTTAACAAGGAAG<br>TTAATGATTGAACTGGGGGTGGGTACGTACTACCCCATGGTACAATACCGTCTTACCAGT<br>CTCCCAGAGGAACAGATAAAGAGGGCGTATAAAATCATAAGGGATGAGGTGAAAACGTGG<br>CCATAGTCCATGAGGAGGTAACGTTTCATACTGAAGTGGATGATGAGAGCGTAGAGGCAA<br>TCAGGAGGATCATAAGGGACCAGTGTGGAGAAAACCCAAAGCCTGAATGTTGGGGCCCGT<br>TGGTGTGGTCCATGTTCGACAGCGTGGCCCGTGCCATCCCGTGCCCAAAGTGCCGAGGGG<br>AGGCACTGGAATTCCTGACCTTTTTGCATGATTTAATTAACATACAGAAGGGTAGTGCTA<br>TATACAATACCCCGCAATTCCTTAGGAAGAAAAAGGAAGAGATACTGAGCCTACTCAGGA<br>CCCGAGGGATATAATATAAATAGGAGAACTGATGTAACTATGGTAGAGAGCCATGGTGTC<br>CAATAGAGAACTCGCCAAAAGGCTCAGCCAGTCGGCCAGGGAGGCGTGGAATTCAGAGGA<br>GGGCATGAGGATCAGAGAGGAGCTAAGTGCAGACTCTGCCTCCTATGGGGCGTGTCTGAG<br>GAGAGTCATGGAAAGTGGAGGAGGCAGTCGTGGTTACAAGGATTGCGCGAGGAGGTCAGG<br>AATAGGTACGGCGTTTGCCAGTGTCTGGGGGTCTACAAAGGCCTATGGTAGGCCGATGGT<br>GGAAGTAGTAGCGAGGTGACCTAGGTGGACTTCTCCAGACAAACGTGGGAGGGGCCAGTG<br>GCTGTTGGCGTCGGCGGGTACGTGTGGAACAAGGTAAAGGGGAACACGCAAGTTCAGACG<br>TTCGTGAGCAAGGTAGGTGGCAAGACGAATGCAGGGCTCATCTTAGTGGCCCTAGGCGTA<br>GTCGCTAAGGCATACAAACCCGAGTCAGAAGCCATGAATATACTGGGGTACCTGTTGGCG<br>GGACTCGGTGCGGGGGCACTCGGTGACGACTTGCCCGCCACTGGGTCACAGGCGACGTTT<br>ACGAATAAAAGCCCAGAGTACCCTAGTCCAGTTAGTGGGGTGAACGTGCTGTGAAACTCT<br>ACATGAAAACGATTGAGATCCAATATTCCATCCCTGCGAGTGCACCATCCGAGTCACAGG<br>TGCCTGGAGTGTTCATAGACCTCTCCAGTGGCACTAGCCAAGGCCAACTCCAAGTCCCCG<br>TGAACCAAGAGTGGGTCATCATAGACGTCTACAACAGGGGGTCTCAGGACGTCGGCGTAG<br>ATGCCGTGGCGACGTTCACTAAGAACAGCGTCTCCAACGTCCTCACTACGGACCCAGTCT<br>CATCCCTGAACATCTCGAACCCATCCAGACCAACCTACCCGCCAATACAGATGGCCCCAG<br>GGACGATATTGACCTCCAAAGTCACTACGCTCACGTCCAACGGCACTAGTGCGGCCGTGG<br>ATAACCTATTCATAAAGGTCAAGATCATAGACTACTCGCAGTAAACACGCGTAAGACATC<br>TCCTTTTTTAGTAGTATTTCCTTCTTTATAGTATGGCATGCCAGACTATACAAGACCTAT<br>CGATAGGCCCGTACACCATAGAATCGGGTCAGGTCTGTGTGGAGGGCACTACCTCAGGGA<br>CGGCTAGCCTGACTATGAAAGTGGGGCCGTTCACGTACACCAAGACTATCCCATGGGGGG<br>CGGTAAACCCCCAACAGACGTTGGGTACGTCCTCAGGGAGGGTCTACCTTATCGGGACGA<br>ACCCCCTGTCGTCACTCCTAGTGGACGACGTCCTAACGGTGGCTGAGTATGCACAGAAAG<br>TGGCGTCGTACTTAGGGTACAGCGGTTCGTTTTCCATAGTGAACGTGGGTTTGACGAATG<br>GCTCAGACTGCAACGACTGTGTAGTTTACGTGGACTACGTCGCCTCTTCTCCTCCTCTTG<br>CGGTAGCCCTAGTGATCGCTCTCGTGATCGTTTTCGTCATAGCTATCGTATTGGGTATCT<br>ATTTCATTTCGGCCGCGATAGAGAGTTTTCAGCCCGCACCTCCCACGCCCCCTCCTCCAG<br>GGAGTCCCCCGTCCCTGTACCAACAGTACTACCAGGAGTACGCCCAGTATTTGAAGGAGA<br>AACAAACGGCCTCAATCACGGGAGGGGTATTCGGCACGTCCACGGCCCTAGTGGCGTTAG<br>GGATAGCCATTTTAGCCTTCTTGGCGTTTACTGACCATGGTGGGAAGTGATGGTGTACCA<br>GATATACGACGGGCAATACACGGTTTACCCCGTGGGAAACCCCCAGGTCACGCTCATCAG<br>GGTAGACGTAGCGGGGAGAGTGAAAATCATGATCCTCCCCACGGTGTCAGAGGCCCTTCA<br>ACTGACCCTGAACGGAAACTCAGGTTACCTGAACCAGGGAAACGTCCTCTCGGCGGGAAA<br>TTGGTACGAGTTCGAATACGTGAGTTGTGGGAAAGACATCATAAGCATAAATGGACACA<br>GCAAGGAGTTTACCTTAGGGTGATAGTGTATTGATTAGAGGTCAGATACACAAACTCTCC<br>AAGTGGGAGTTAAGGGACTACGCTAGGACACTAGTCCCCGTCTTTGAATCCCACGAGGTG<br>GACGGCACTAGGGACATGAAAATCGTGGACGCCGTGATATCGTACGCCATGAAAGGGACG<br>AAACCCTGGCTGAGGAGGGGGTCAGCCCTCCAGGGCTTTATAGGGCCTAGGATGTCTGTC<br>CTCGAGGGTTACCTCATCTTCCCCACGTCAGGTATTGAGCCCGTGACCATCCAGTCCACT<br>ACCCTAGAGAACTCCTACCTAGTCCTAGATATCGTGGGGGCAGGTGGAGGAGGTGGTGGT<br>GGAGGTGGTGGGAACACCACGGCCACTACCCCGTTCGCCCAGGGTGGTGGAGGTGGGGGA<br>GACGGTGGCCGTCTCATTATCAGGACCCAGGGGCCACTAGTCTCTGGGACCCAGTTCACA

	GCCCAACTGGGAGGGCCAGGTGGGACAGGTGGAGGGACAGGTGCCACAGGGACACCAGGT<br>TCAGCACCAAACGGGGCGACTCAGGTAGTCCTAGGGAACGGCACACTCACGCTCAGTGTG<br>TCAGGTGCGTCAGCCCCTACCATGCCCCCGACAGCAGGAGGACAGGAGGCAGGAGGGTCA<br>GGAGGTGCAGGAGGGTCAGCAGTGTTCACGTACTTGCCAACGACGCCCATATTCTCGGCC<br>ATACAGTTGAACCCAAGGACGTCCTTCCTCTCGAATTACTCGGGCGTACTGGACCAGGGT<br>ATTGGTGGAGACGGCGTTGGGTCTGAGTACTTCACCCTGTCCAACGCCACGCCTATATTC<br>GTCTCGACGCCACAAGGAACGAGGGTCACTGGGGGAAGCCCAGGGGGCACAGGCACCTCA<br>GCCACGGCGAACGTCCCCTCTGGGGCAATCCCGCTCAGGGGGTCAGGTGCAGGAGGAGGA<br>GGGGGACAGAACTCCTCTTCCGCCCCAACAGCGGGAGGGAACGGCGGGTCTGGAGGGCCT<br>GGACCTATCATTATCTTGGTGATAGGATGACAGACTTGGTCACCGTGGCAGTGTTAGGGG<br>TACAGACGGCCGTTTTCATCTACGCTAGTGCCAAACTGAGGAAGATGAGCGAGATGGCCG<br>ACAAGTTGGCGTCAGCCCTGTCCAACATAGCAAAGGAGATAGAGACGGCGTCCATGGACA<br>TGAAAAGCATACAAAGGGAGATGACAGAACTGGAAGACTTAATTCGGACAGCACTGAGGG<br>AATAGGTATGAGCTGTCAGACATGTGGTCAGCAGGGCGTGTGTTACGTCCTGGAACACGT<br>GGGTACTGAGCCCATCTATATAAACGGGCAACAGTACGGCCAGGGCGACTTTTGTTTCTC<br>CTCCCCTGAGCTCACGATAGAATACCAGGGCGTACAGTACCAGATCGACATGTCCTACGC<br>CCCCACAGTGCAGAACATATACGGGTGTTGTCCCTCAGGTACCACAGCCAAACTCACGAC<br>GGTGACTACGATACAGGGACAGAACACTCCCATATTCGTCCCGTATGCCGTCCTAGTGTC<br>TCAAGGTACCACGTCCACTAGTAGTACCACGCCTAGTGTCCTAAGTAAGGTACCGTGGTG<br>GCTCATCATACTACTCGTCATACTGGGGGCTATACTCCTAGGAGGAGGAAACCCATGAAT<br>TACACTTGGTACGTGCAGAACCAAGACCCCTCGACGATCCAGTTCTGTGTCATGCAGTCT<br>GGAGGGTATAACAACTGCACCTCAATACCAGGGAACAGCACAGAGCCGTTCACGATACCA<br>GACATGGACGTGCAGATATATGGCACCCCCACGGGGTACTACGTGTACTACTCTAGCGTT<br>GGGGTGAAGACTAGTACCAAGTCCCTCCCGTCATCAGGATCAACGATCACGGTGCCCCCA<br>TACACGGGATGTGACCCCCTACTCACGCAAGGACAGGGGTACCCCCCACTCGTGCCGTGT<br>TCGTCCCCGTTGATCTTCCTGAAGAGCGAGAGCACCCCTGCCATGTTTATCGAGAACTGG<br>GGGGCAGAGACGTCAGGGTTTTCATACGGGTACGGGATAGCGGGGAGCCAATTGGCCATA<br>GCGACAGGGCCAAACTGGGTATACTACCGACAGTACCCCCAGGCACAGGTATACCTTATC<br>GGAAACTGGAAGGTGGTGAACGGAAACTACGTAGACATGGACTCGGGGAAAACGTTCTGT<br>GGGTGCCAGGGGAAGAGCGTGTACGTCATACCCACGTCTGACCTACCTGGGCCGTCCTCA<br>GGGACTACGTCCACAGGGACTAGTACCACGGGGACTTCCACGAGTACAACAGGGACTCCC<br>ACAGGGACGTCCACTCAGCAGACGACACAGCCCACGACAGGTATCCTGAACGTGGCTAAC<br>CAGTGTTCGACGGGGGTAGTCGTGTACGCGGGAGGGAAAACGGCATACGCACCCCCATAC<br>CATTCCATCAGCCTAGAAGTACCCATCCCCACGGGGTATACGCTTATTAGCCAGGGTCAG<br>ACAGTTGGTAAGGGGACGGTGTACCAGAGCGGGGAGTACGTGTCCCCAAGTTCTTGTCCC<br>TCTGTTAGCCCTACACCCAGCCCTACCCCGACACCGTCCCCAACTGGGGGACAGACCACT<br>TCCACGACTACGACTACCACGACTACCCAGACCACTTCAACACAGACTACCCAGAACACA<br>CAGCCCCCAACGACGCCTAGCATGTCGTGGGGTCTTATCCTACTCTTGGTAATCCTGGGG<br>GCACTAGCCCTCTCAAGTTAAGCTTTTTTAGTCCAGGTCTCCCATTATACCTGAGCGGTG<br>CGAGGTGGATGTGACGGGAGGCTCTGGGAATGTCCGAGAGACGGAGGCGTACCCGTCCTC<br>CACTGCACCGCACTAGGTGAGTCACATGAGCCAAGTCCCAAAGAAGAGACTCGGGGCAAG<br>GGTCATCAGAGCGGTTTTCAACGGGGTCTCCTTAGGCATCCTAGGAGGGGCAGGAGTGTA<br>CGTCCTAGCCCAGGGGGTGAACACGTTATCAGGCACCACTACTTTGAACCCTACGGCGTT<br>TCTCCTGTTGGTCTTGGGGGCGTCCATCACGGCCAGTGTGGGGATAGAACTCTCCAAGGA<br>CCTAAGCGGTGAGTAGTCTCAGTCAAGTCTCCTTTATTTTACCCCCTGAACCAATAGACC<br>CAAAATGGATGTCCAAACCCAACTCAAAGAATGGATTTCCCTGTTGGACGACAGGCTGTC<br>GTCCATAGAAATGAGCCTCAAGTCCCTCCAAAAGAGACTTGAGCAGGCAGAAAAGCGACA<br>AGAAGAACTTGAGGAGGCGTTCGACCAGCTGAAAGAAGAAATAGACGACTGTGTGGCTGA<br>CACGACCTACCTCCACGACGTACTGGAGGAGGTCCAAGACGACATAGACGACCTAAGGAA<br>GTGAGCCCCGTGGAGTTCGTATACAAGATCAACGACGACACGGTCACAGTGTGGGTCACT<br>GACGAGAAGACGGGGAAGAAAGTGGCGTCCCTACAAATGCCGAAGAAGGACGTGGACTAC<br>GTCGCGAGACGTGCAATACAAGAACTCGACATAGCCCCAGATGAGGCATACGACTTAGCG<br>GTCTACTTCACCTACGTTAAGGTCACCTGGGGCCTCCTCTGAACCTCTTTTTTCTCTCGT<br>ACTTACCCCTGACCCTACGGTCAGGCTCTACTTCTCTCACGAACCTGACTCCCCCATCCT<br>CATCAAACATATGATTTATTTCACAGCAGGGCTTTATATAAATTGCGGAAGGTCGTGATG<br>GGGGGGTTAAAATGCTTTTTGCCCTGTTGGAGAGAACCCTTCACTCTGAGGTTTACTTTC<br>GGTTTGTGTCGTTAAGTGTACTTGCAGGGCGGGGCAGGGCGTATTATGGGCTGTAATTTG<br>CTCATTCGCATTAGGACAAAAACCCCTGGGGTTAATAAGCTTTCATGCTAGGGGGGGTAT<br>TTAAACCCTCCCCGCGTTCAACTTGCGTGGTGATCCGTCAAACTGACGTGGCTTTTAAAC<br>CCCCTGCTCTGGGGGGGGTAGGCTAGGTCTCGCCCTCCCTTTCGGCATGGGGGGGGGGGG

Supplementary Figure 1: Rank abundance curves for metagenomes based on ribosomal protein S3 indicating dominance of Altiarchaeota in the MSI ecosystem. for A) MSI_BF_2018, B) MSI_<0.1µm_2018, C) MSI_BF_2012, and D) MSI_>0.1µm_2018 (stretched over two panels). Assemblies for 2018 samples were performed with MetaSPADES 3.10 [2], whereas for the 2012 assembly MetaSPADES 3.11 has been used.

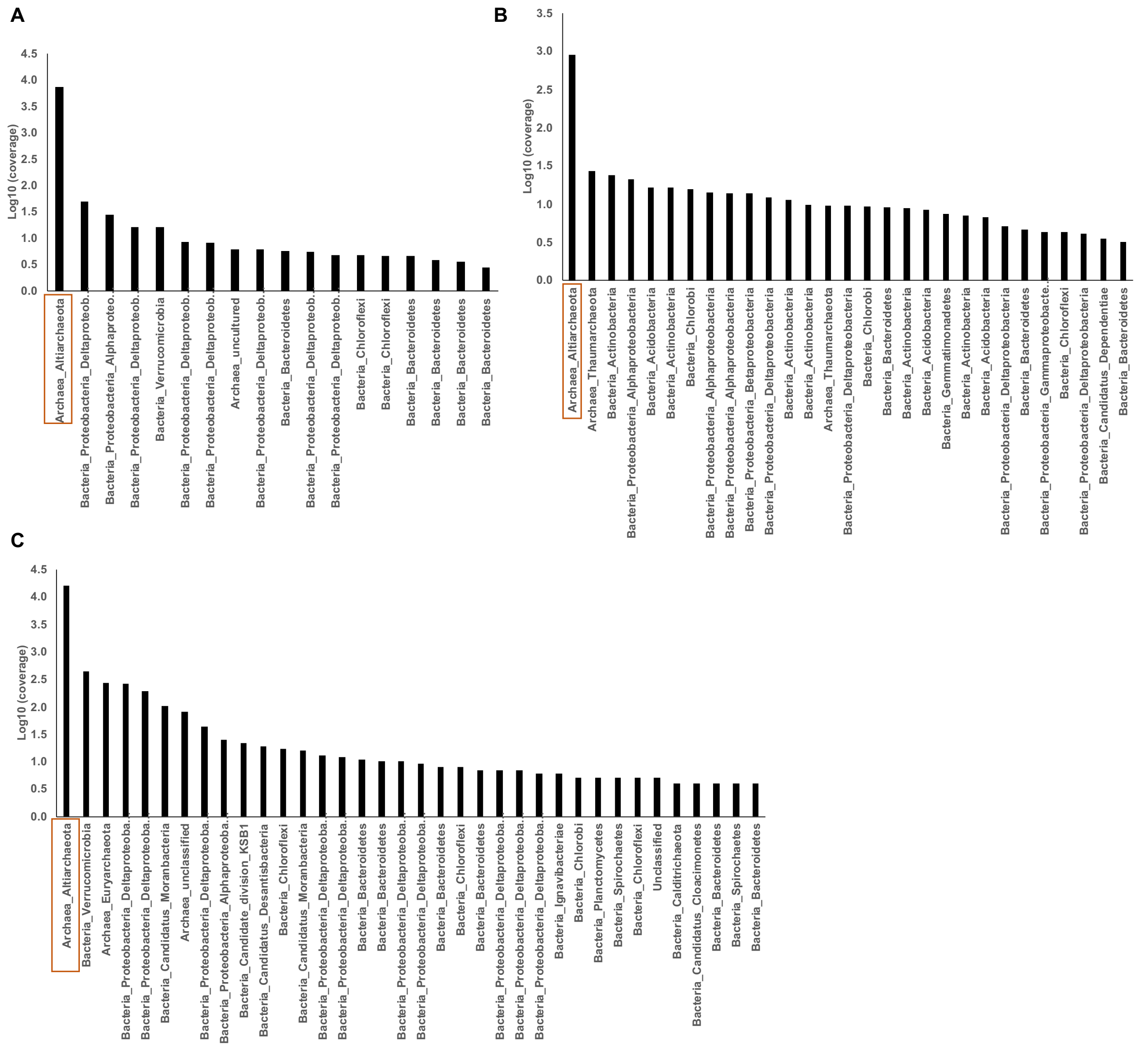

D

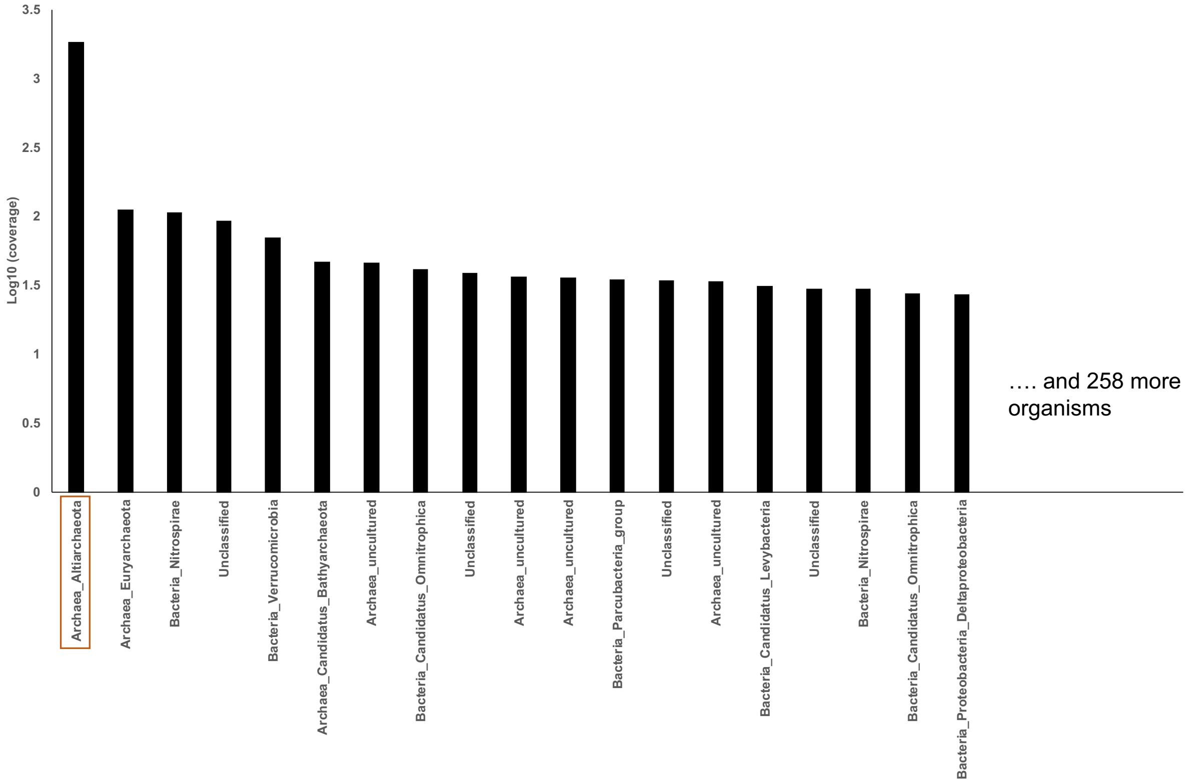

Log10 (coverage)
3.5
3
2.5
2
1.5
1
0.5
0
Archaea_Altiarchaeota
Archaea_Euryarchaeota
Bacteria_Nitrospirae
Unclassified
Bacteria_Verrucomicrobia
Archaea_Candidatus_Bathyarchaeota
Archaea_uncultured
Bacteria_Candidatus_Omnitrophica
Unclassified
Archaea_uncultured
Archaea_uncultured
Bacteria_Parcubacteria_group
Unclassified
Archaea_uncultured
Bacteria_Candidatus_Levybacteria
Unclassified
Bacteria_Nitrospirae
Bacteria_Candidatus_Omnitrophica
Bacteria_Proteobacteria_Deltaproteobacteria
…. and 258 more organisms

Supplementary Figure 2: Two distinct direct repeat sequences of CRISPR arrays derived from Altiarchaeota and formation of secondary structures. A) Alignment of CRISPR repeat sequences from Altiarchaeota genomes obtained from different habitats (Supplementary Data 1). All direct repeat sequences end with a AAA(N) motif. Alignment was performed using MUSCLE within Geneious v.11.1.5. [3]. B) CRISPR RNA (cRNA) secondary structure predictions of the different repeats and their orientations predicted by RNAfold (http://rna.tbi.univie.ac.at/cgi-bin/RNAWebSuite/RNAfold.cgi) [4].

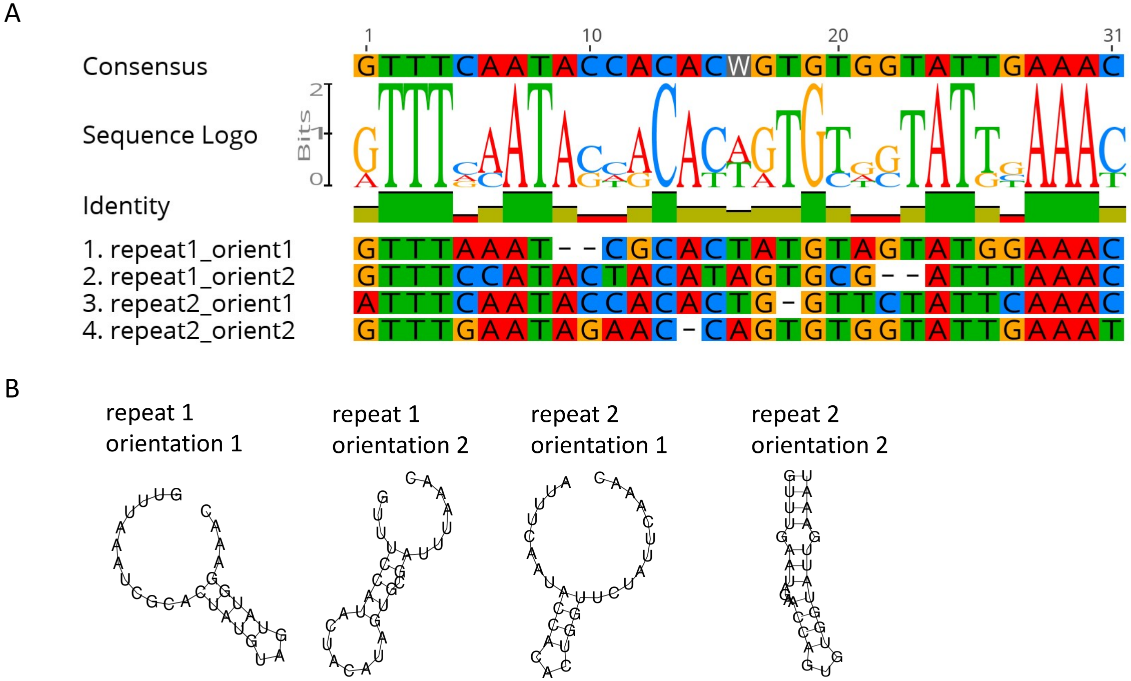

Supplementary Figure 3: Classification scheme for viral scaffolds. A) Bioinformatic tools (VirSorter v.1 [5], VirFinder v.1.1 [6], VOGDB [7] (version Vog93, e-value cut-off 10^{-5}), CircMG [8] and EndMatcher (https://github.com/ProbstLab/viromics/tree/master/Endmatcher) used for detecting viral scaffolds; B) Combination of results and how it leads to classification of predicted viruses into putative viruses and viruses. Modified from Eßer [9]. VirFinder [6] hits were false discovery rate-corrected using the package q value [10] in the R programming environment [11] applying a p-value cut-off of <0.05. Colors indicate different tools as follows: grey=CircMG, orange=Endmatcher, blue=VirFinder, yellow=VogDB, various colors for VirSorter categories.

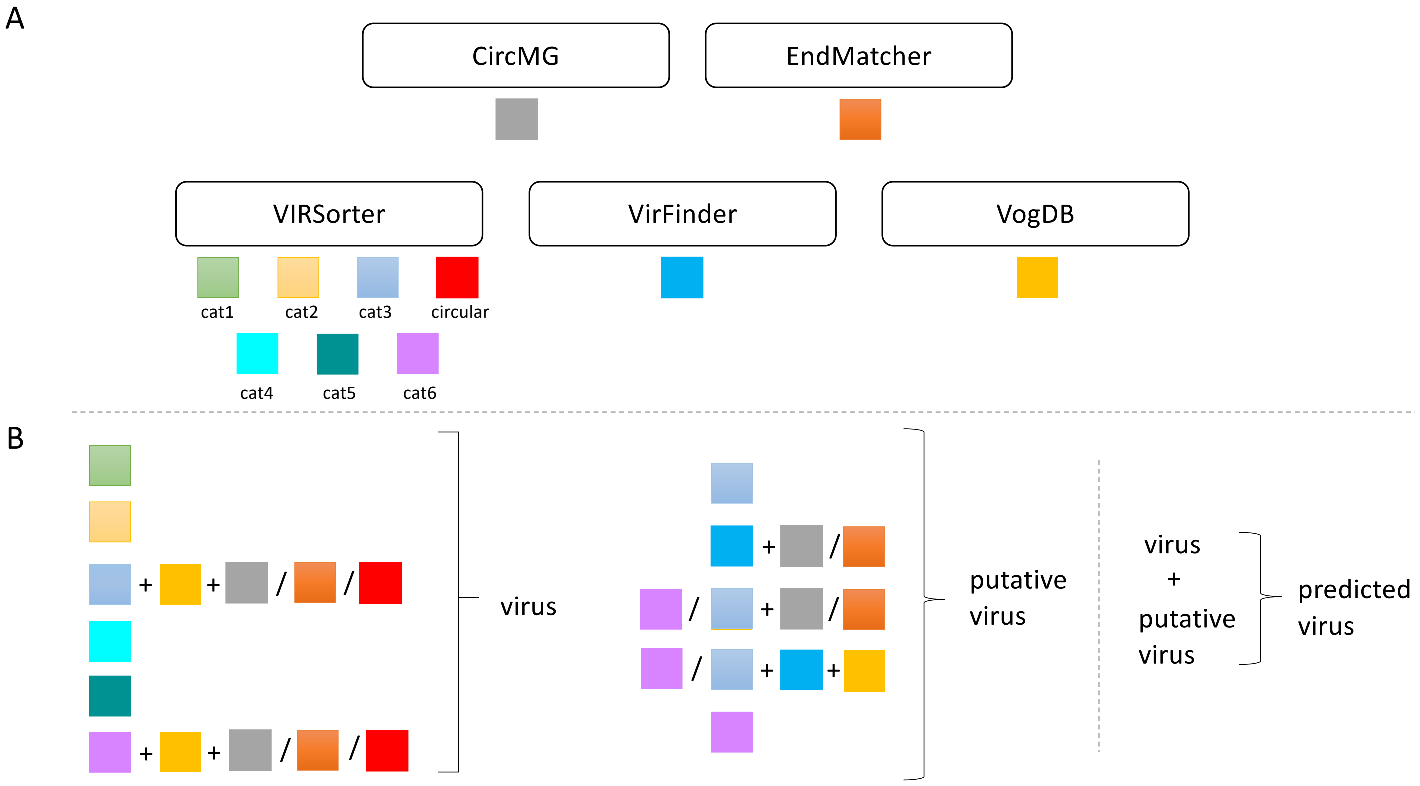

Supplementary Figure 4: Intergenomic similarities between *Ca.* Altiarchaeum virus Altivir genomes calculated by VIRIDIC (http://viridic.icbm.de /) [12]. ACLF=Alpena County Library Fountain, BF=biofilm, HURL=Horonobe Underground Research Laboratory, MSI=Mühlbacher Schwefelquelle Isling.

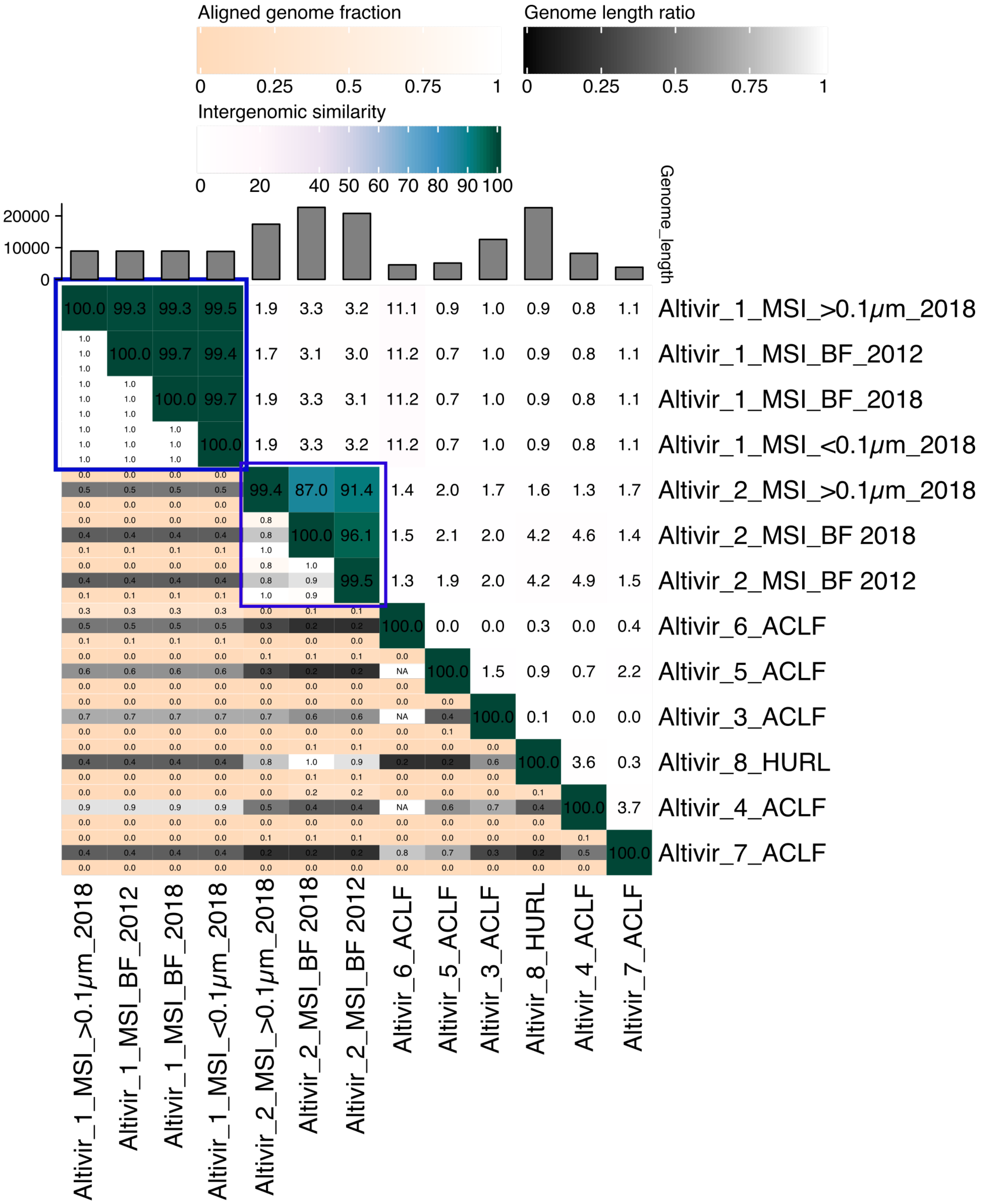

Supplementary Figure 5: Viral clusters of Altivir_1, _2, and _6 and their position in the viral network. Altivir_1_MSI genomes form a cluster with Altivir_6_ACLF while Altivir_2_MSI genomes form a cluster on their own. The network shows how genomes cluster according to similarities with the database "ProkaryoticViralRefSeq94-Merged" [13] after using vConTACT v.0.9.11 [14, 15]. Nodes and edges represent viral genomes and genome similarities, respectively. Visualization was done in Cytoscape v.3.7.2 [16]. All remaining genomes of *Ca.* Altiarchaeum viruses were excluded as unclustered singletons. ACLF=Alpena County Library Fountain, BF=biofilm, MSI=Mühlbacher Schwefelquelle Isling.

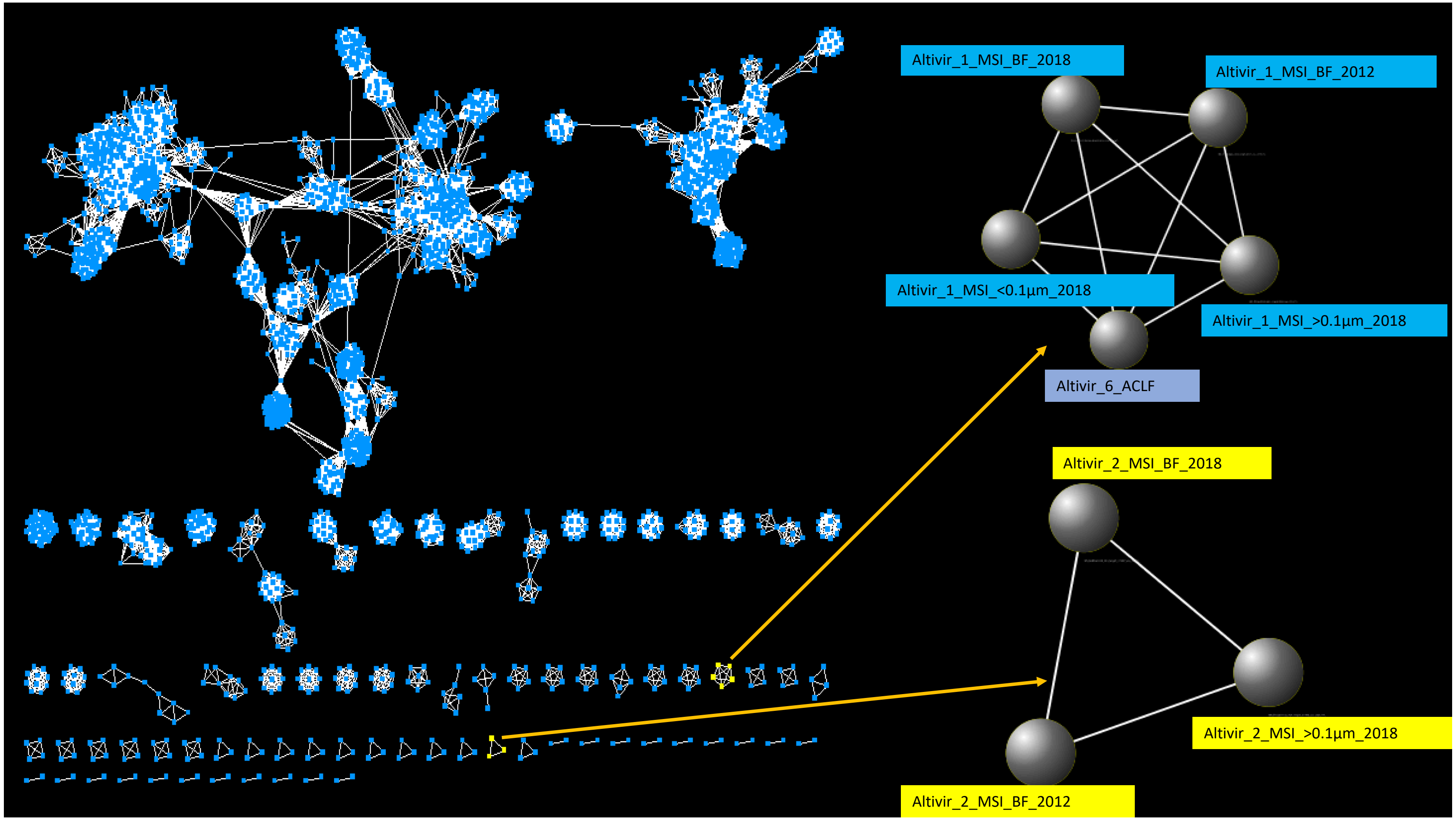

Supplementary Figure 6: Phylogenetic tree for the DNA polymerase of Altivir_2_MSI and related proteins. Internal branch labels represent branch support values, which have been calculated using the approximate likelihood-ratio test. The tree was rooted at midpoint. For further details, see Materials and Methods section.

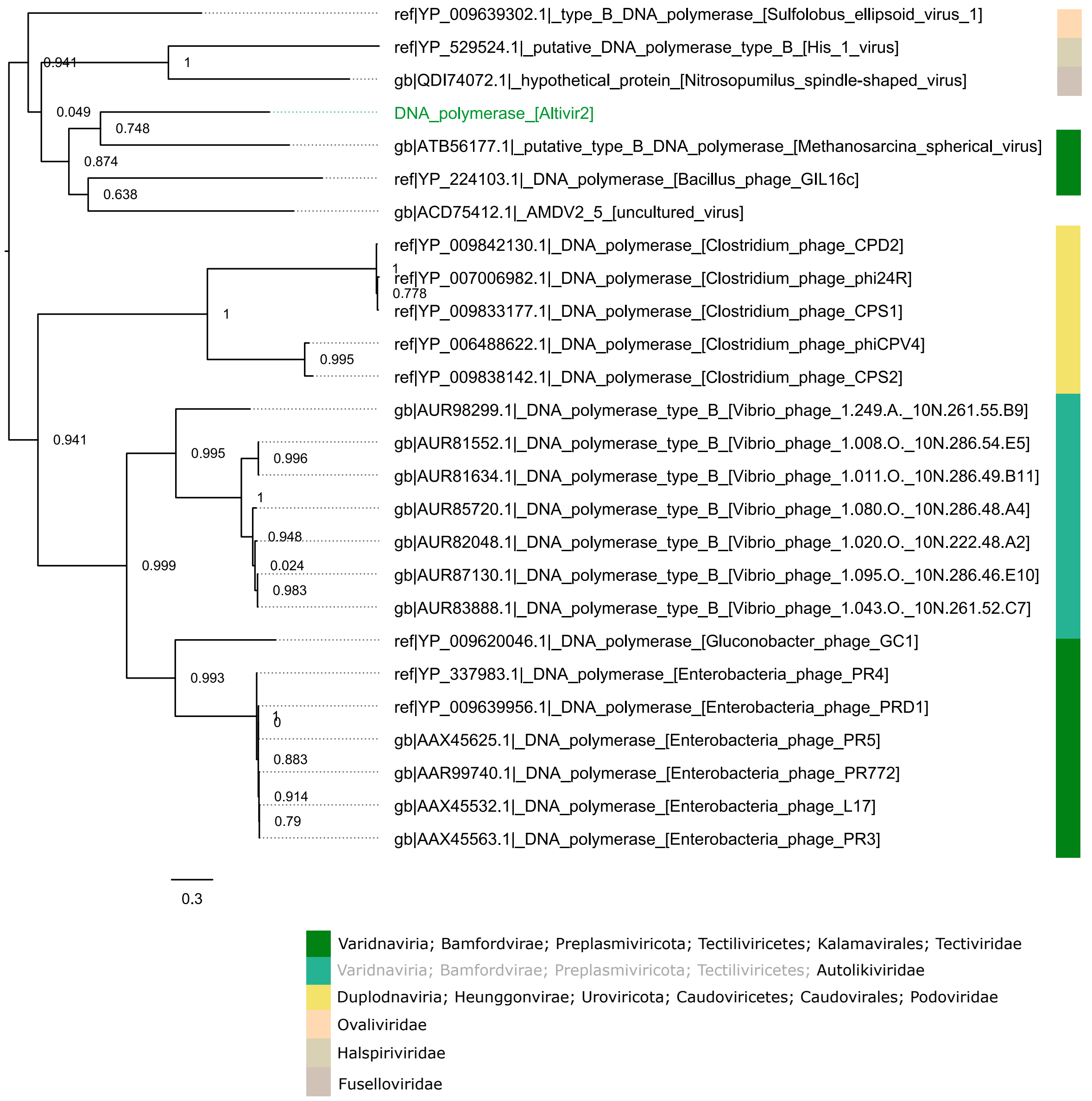

Supplementary Figure 7: Phylogenetic tree of the capsid portal protein of Altivir_4_ACLF and related proteins. Internal branch labels represent branch support values, which have been calculated using the approximate likelihood-ratio test. The tree was rooted at midpoint. For further details, see Materials and Methods section.

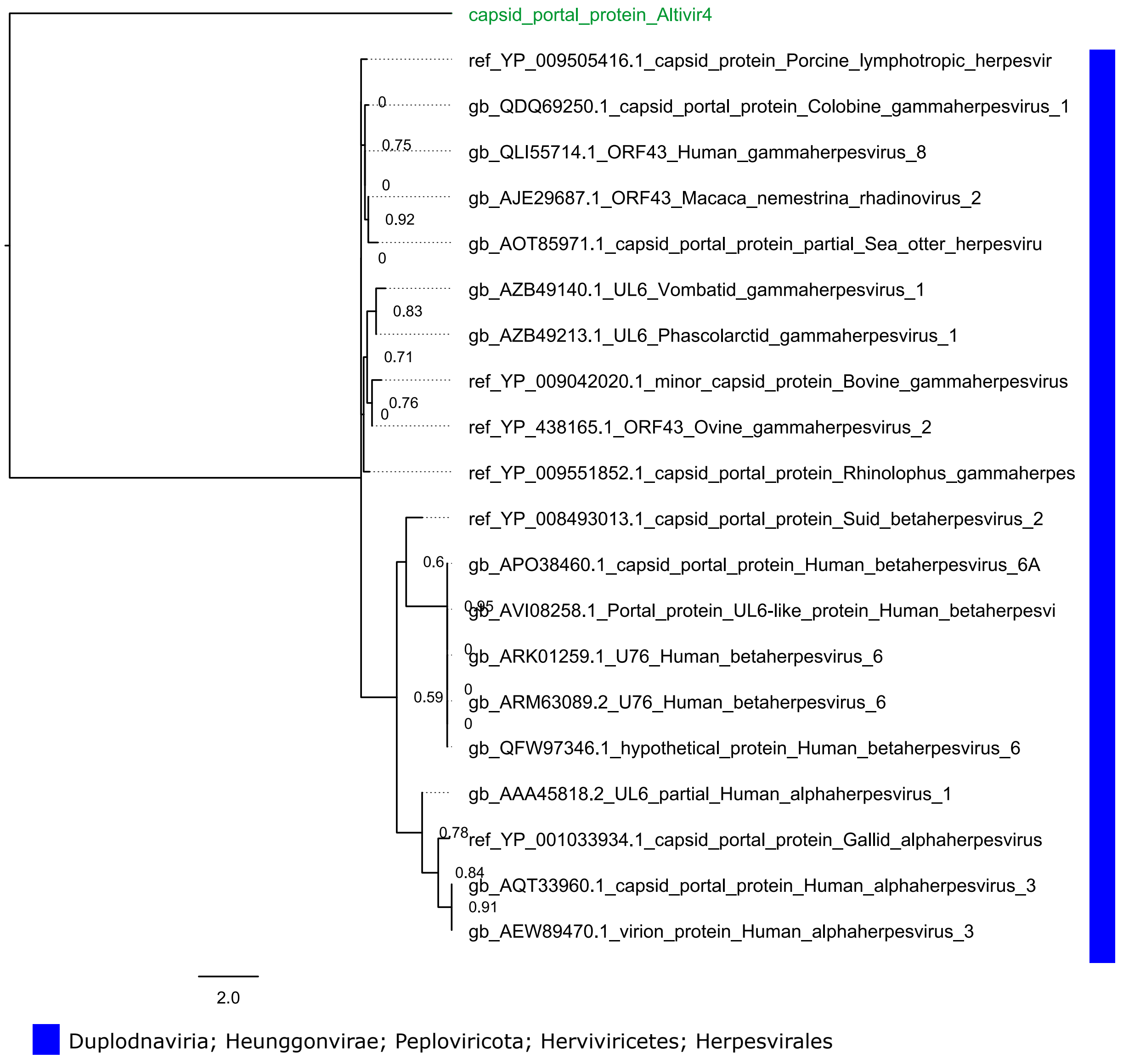

Supplementary Figure 8: Phylogenetic tree for the major tail tube protein of Altivir_7_ACLF and related proteins. Internal branch labels represent branch support values, which have been calculated using the approximate likelihood-ratio test. The tree was rooted at midpoint. For further details, see Materials and Methods section.

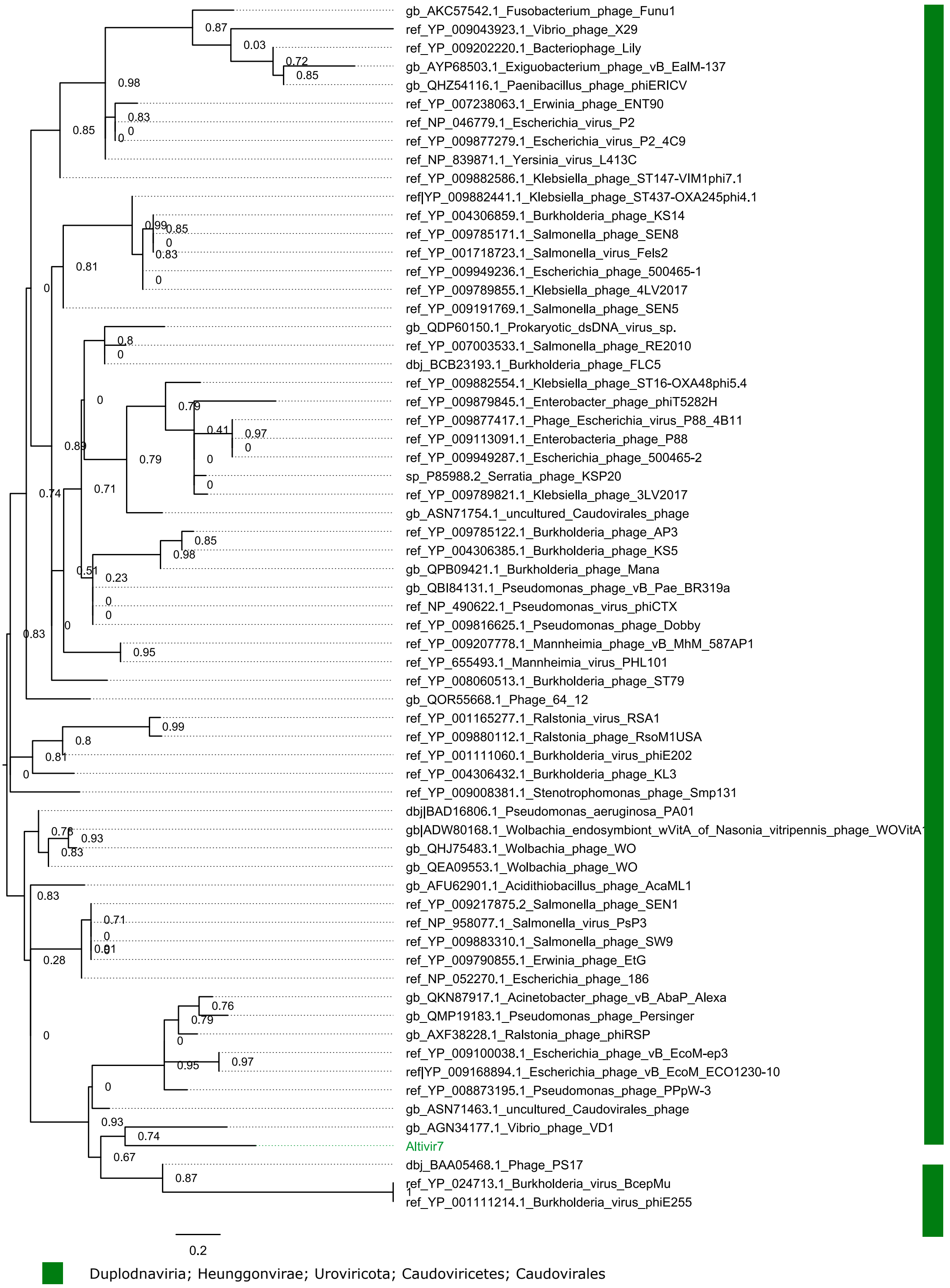

Supplementary Figure 9: Phylogenetic tree for the terminase protein of Altivir_8_HURL and related proteins. Internal branch labels represent branch support values, which have been calculated using the approximate likelihood-ratio test. The tree was rooted at midpoint. For further details, see Materials and Methods section.

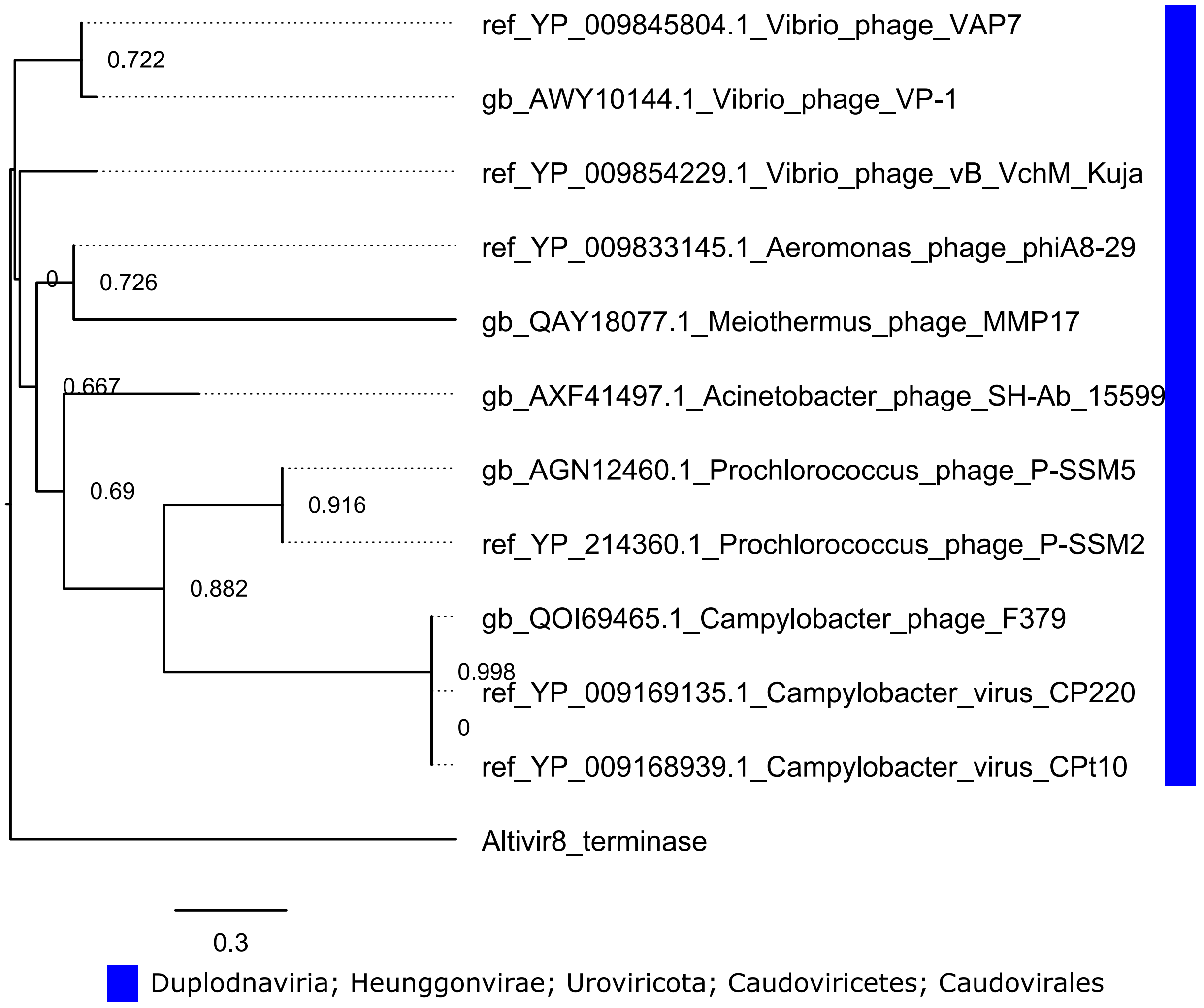

Supplementary Figure 10: Development of coding sequences (CDS) from 2012 and 2018 samples for Altivir_1_MSI (upper panel) and Altivir_2_MSI (lower panel). Variant regions can only be shown for Altivir_1_MSI due to the huge gaps in beginning and end of the Altivir_2_MSI alignments. Alignments were performed using MUSCLE [3], and variant calling was performed with default settings of Geneious Prime version 11.1.5 [17]. MSI=Mühlbacher Schwefelquelle Isling.

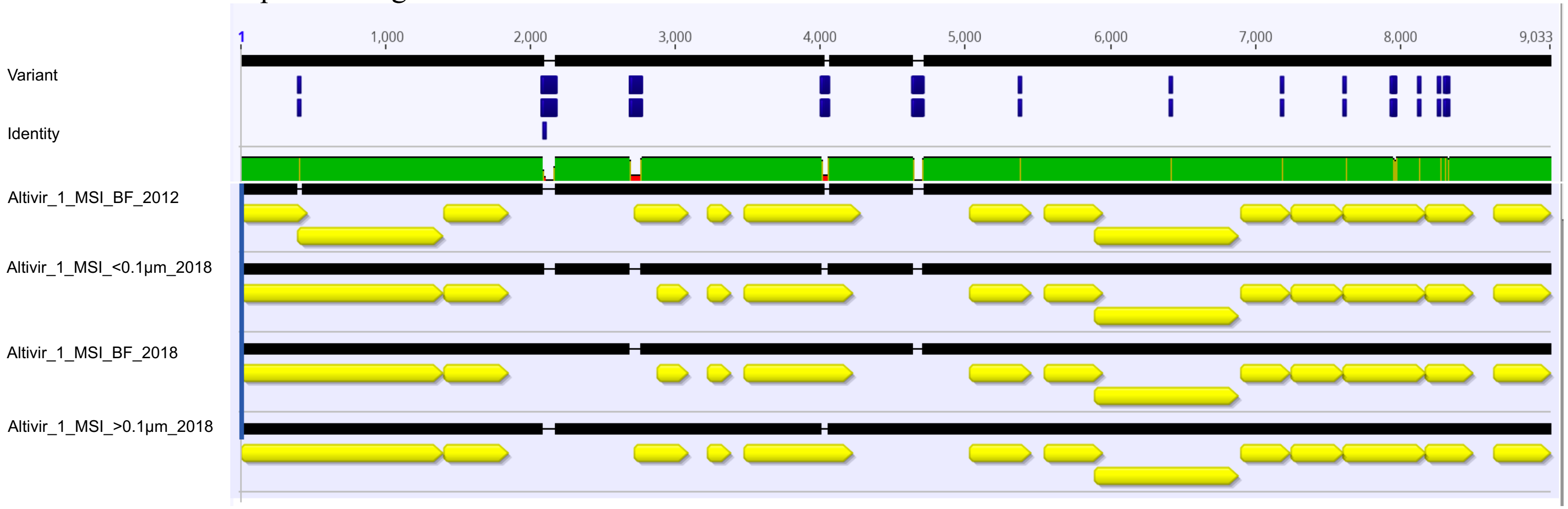

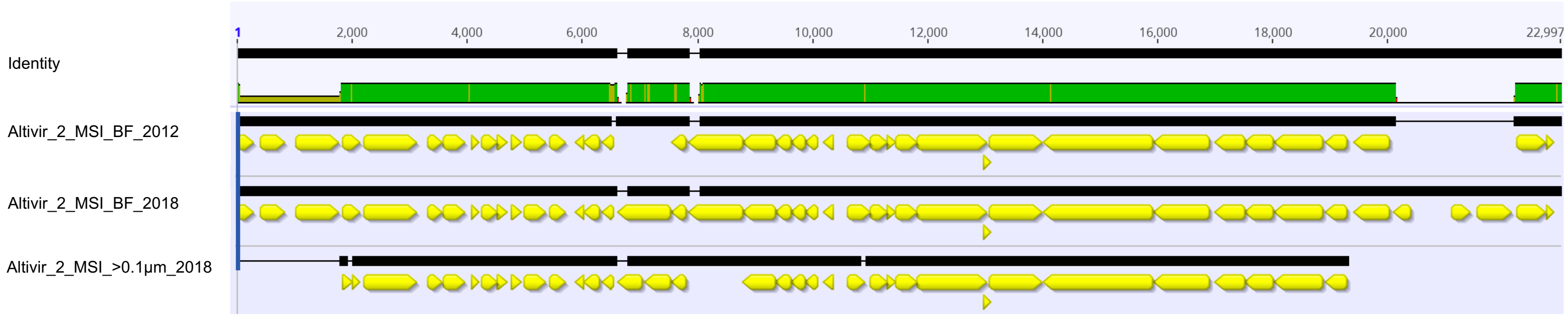

Supplementary Figure 11: VirusFISH performed on Altiarchaeota biofilms (BF) for visualization of viral infections caused by Altivir_1_MSI. For virusFISH, a chemically synthesized probe mix targeting Altivir_1_MSI was used. BF material was analyzed with A) DAPI (blue, cells), B) ATTO 488 (purple, 16S rRNA signal) and C) Alexa 594 (yellow, viral genomes of Altivir_1_MSI) and D) merged. VirusFISH experiments were highly reproducible: Since we established virusFISH for this project in 2019, we have tested the chemically synthesized probe mix targeting Altivir_1_MSI 70 times in total and the Metallosphaera sp. virus probe (as negative control) 66 times in total. The testing was always carried out on Altiarchaea biofilm samples from MSI site. Scale bars: 10 µm.

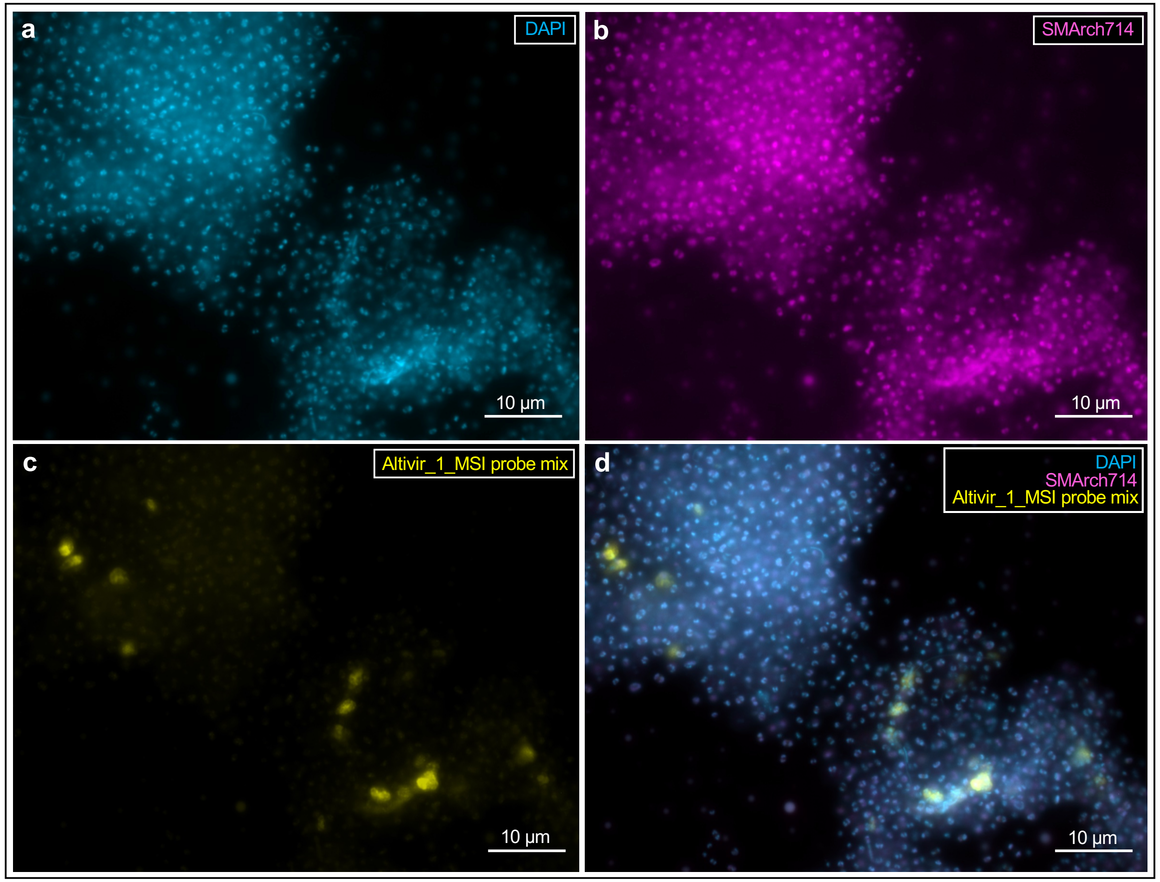

Supplementary Figure 12: A chemically synthesized *Metallosphaera* sp. virus probe was used as a non-matching probe for virusFISH showing no viral infections within an altiarchaeotal biofilm (BF). BF material was analyzed with A) DAPI (blue, cells), B) ATTO 488 (purple, 16S rRNA signal) and C) Alexa 594 (yellow) and merged (D). For a statement regarding reproducibility please see legend of Supplementary Figure 11. Scale bars: 10 µm.

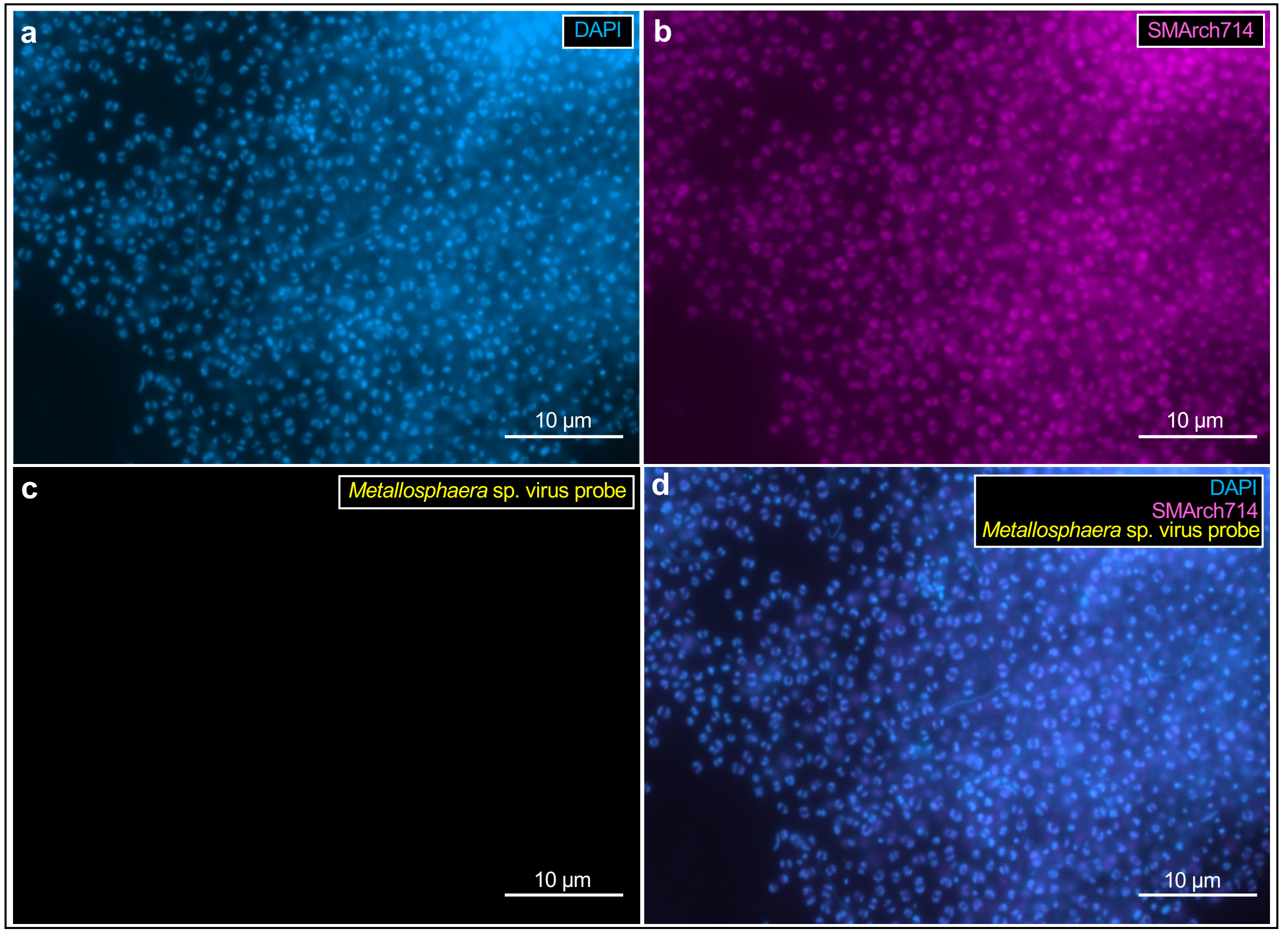

Supplementary Figure 13: Bar graph depicting the number of spacers found as singletons compared to the total number of different spacers per sample from the MSI site. An increase by ~20% from 2012 to 2018 is identified, which indicates a diversification of the CRISPR array in Altiarchaeota and of their respective strains. BF=biofilm, MSI=Mühlbacher Schwefelquelle Isling, CRISPR=Clustered regularly interspaced short palindromic repeat.

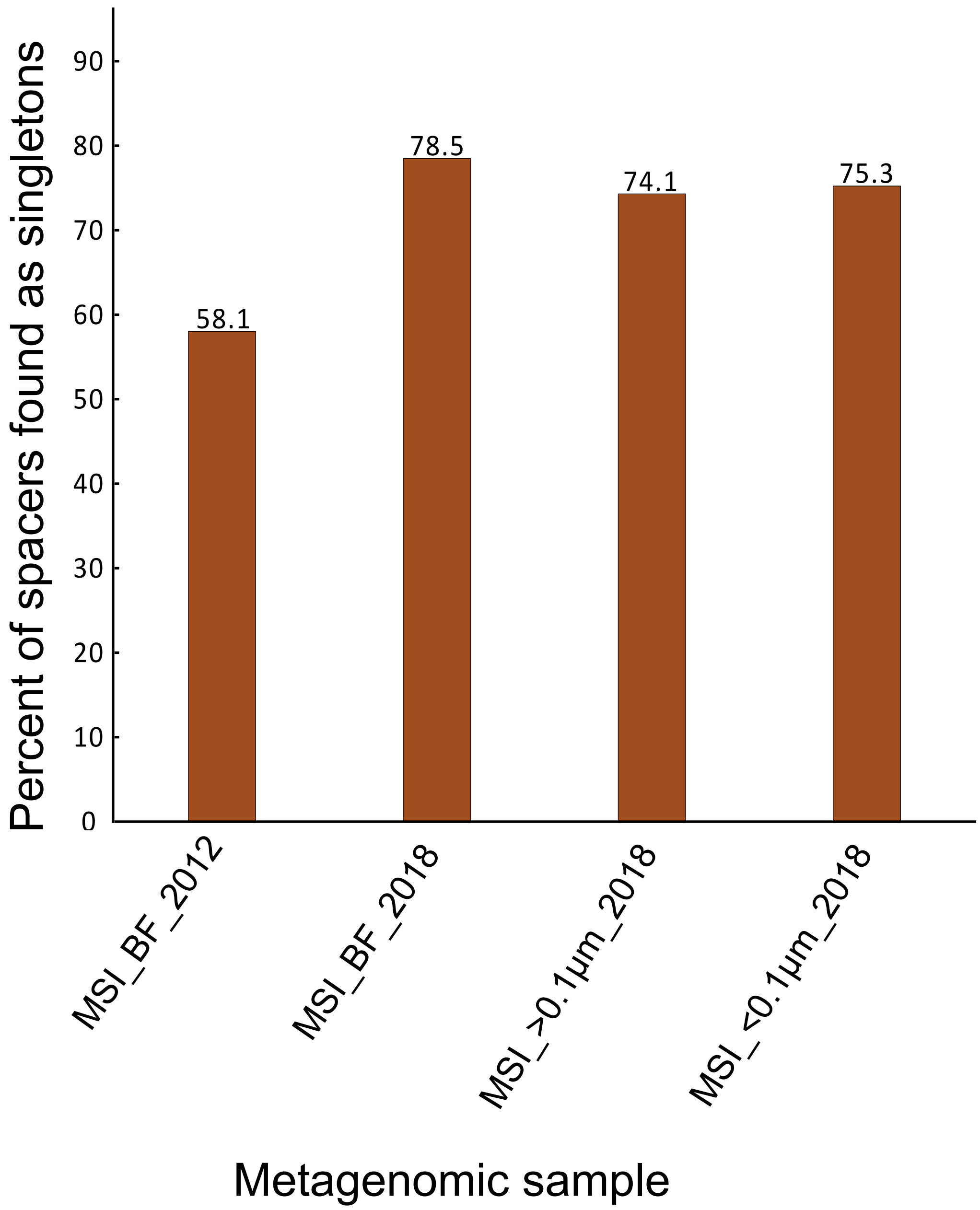

Supplementary Figure 14: Spacer mapping to predicted viral genomes and variants therein. A) Spacers extracted from MSI_BF_2012 metagenomes mapped against genomes of Altivir_1_MSI_BF_2012 and B) Altivir_2_MSI_BF_2012. C) Spacers extracted from MSI_BF_2018 metagenomes mapped against genomes of Altivir_1_MSI_BF_2012 and D) Altivir_2_MSI_BF_2012. Variants (abbreviated to SNP) are based on read mapping from reads of MSI_BF_2012 and MSI_BF_2018 samples (see Supplementary Data 2) and coding regions (in yellow) are shown. Variant calling was performed in default settings of Geneious Prime version 11.1.5 [17]. Mapping of paired reads to viral genomes was conducted using Bowtie 2 sensitive mode [18]. BF=biofilm, MSI=Mühlbacher Schwefelquelle Isling, SNP=single-nucleotide polymorphism.

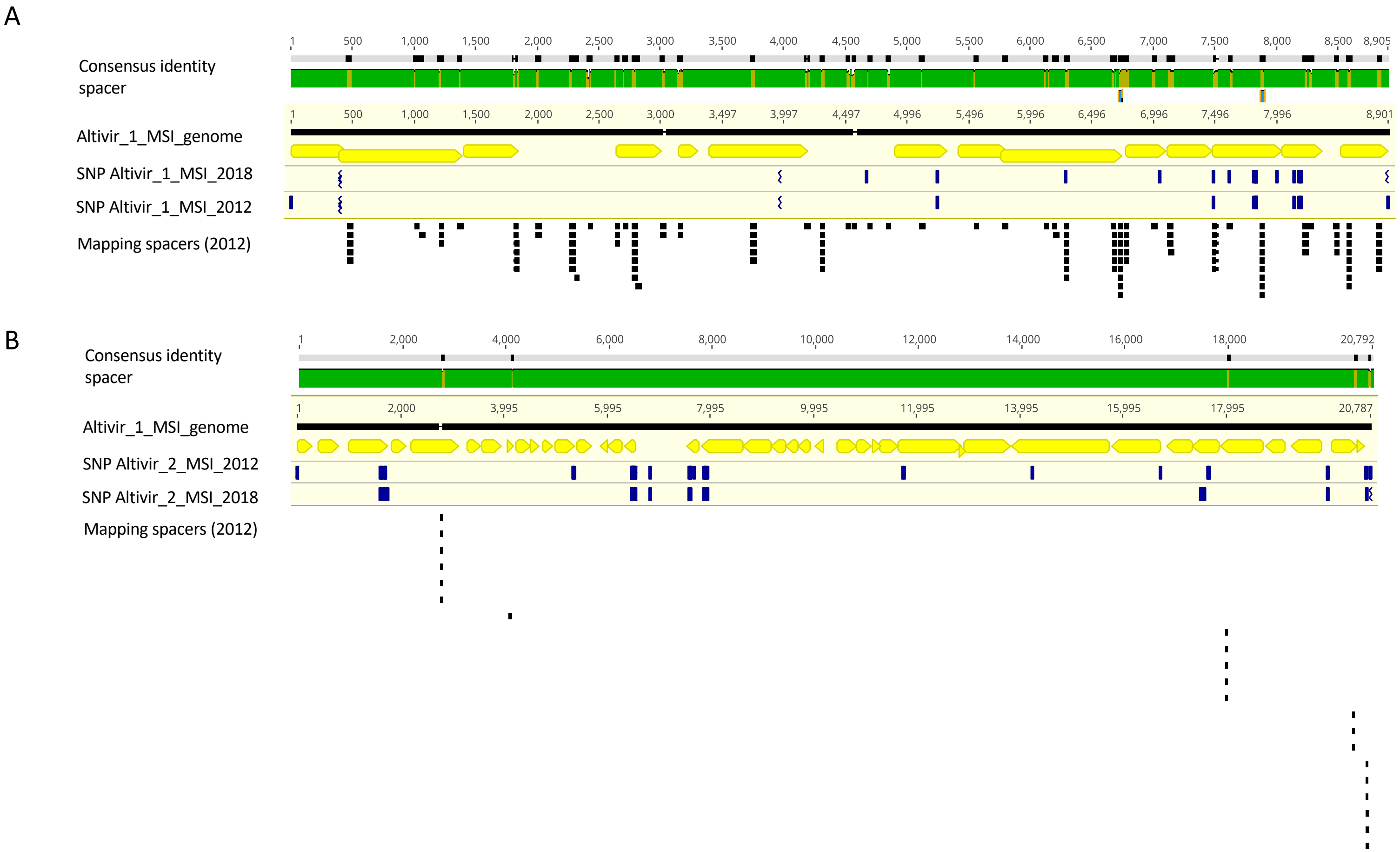

C

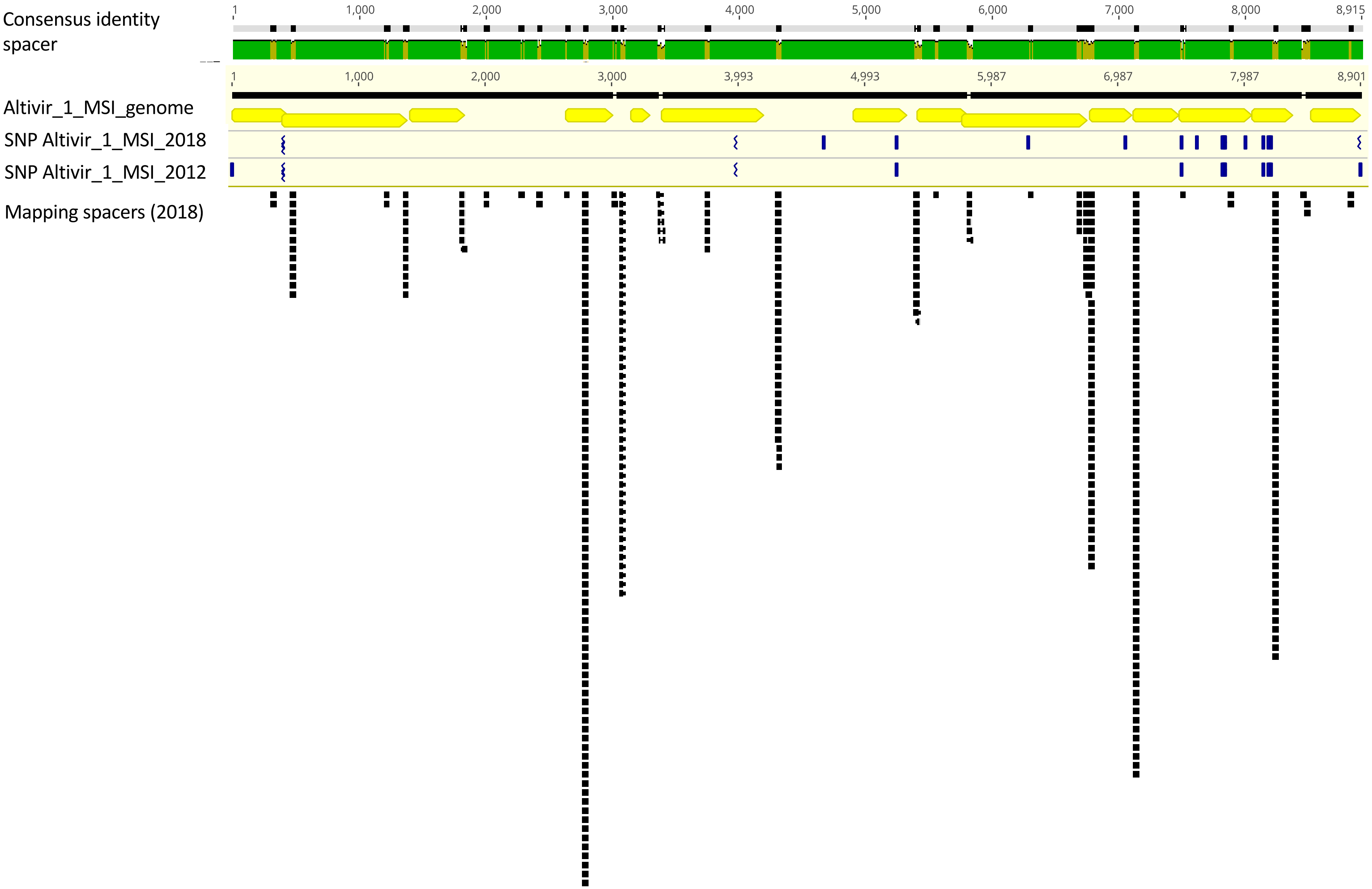
Consensus identity
spacer
1
1,000
2,000
3,000
4,000
5,000
6,000
7,000
8,000
8,915
1
1,000
2,000
3,000
3,993
4,993
5,987
6,987
7,987
8,901
Altivir_1_MSI_genome
SNP Altivir_1_MSI_2018
SNP Altivir_1_MSI_2012
Mapping spacers (2018)

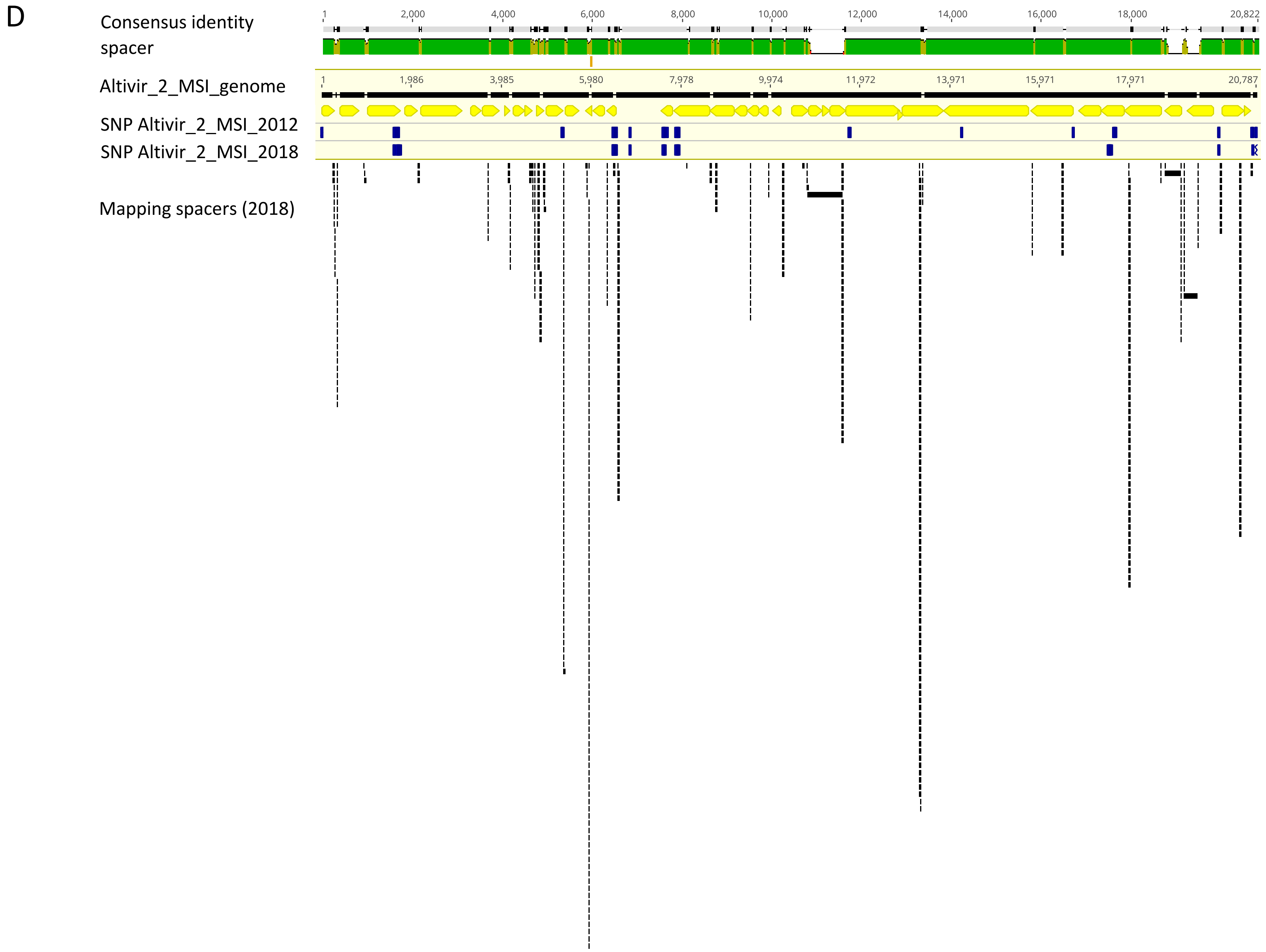
D
Consensus identity
spacer
Altivir_2_MSI_genome
SNP Altivir_2_MSI_2012
SNP Altivir_2_MSI_2018
Mapping spacers (2018)
1
2,000
4,000
6,000
8,000
10,000
12,000
14,000
16,000
18,000
20,822
1
1,986
3,985
5,980
7,978
9,974
11,972
13,971
15,971
17,971
20,787

Supplementary Figure 15: Venn diagrams for shared spacer clusters between samples and years for A) total clusters, B) spacer clusters matching Altivir_1_MSI, and C) spacer clusters matching Altivir_2_MSI. The number of shared clusters might deviate from Figure 4, which show read-normalized spacer abundances (rel. abundances). Diagrams were constructed using the UGent webtool (http://bioinformatics.psb.ugent.be/webtools/Venn/). Colors indicate different samples as follows: blue=BF_2012, red=BF_2018, green=>0.1 µm_2018, yellow=<0.1 µm_2018; BF=biofilm.

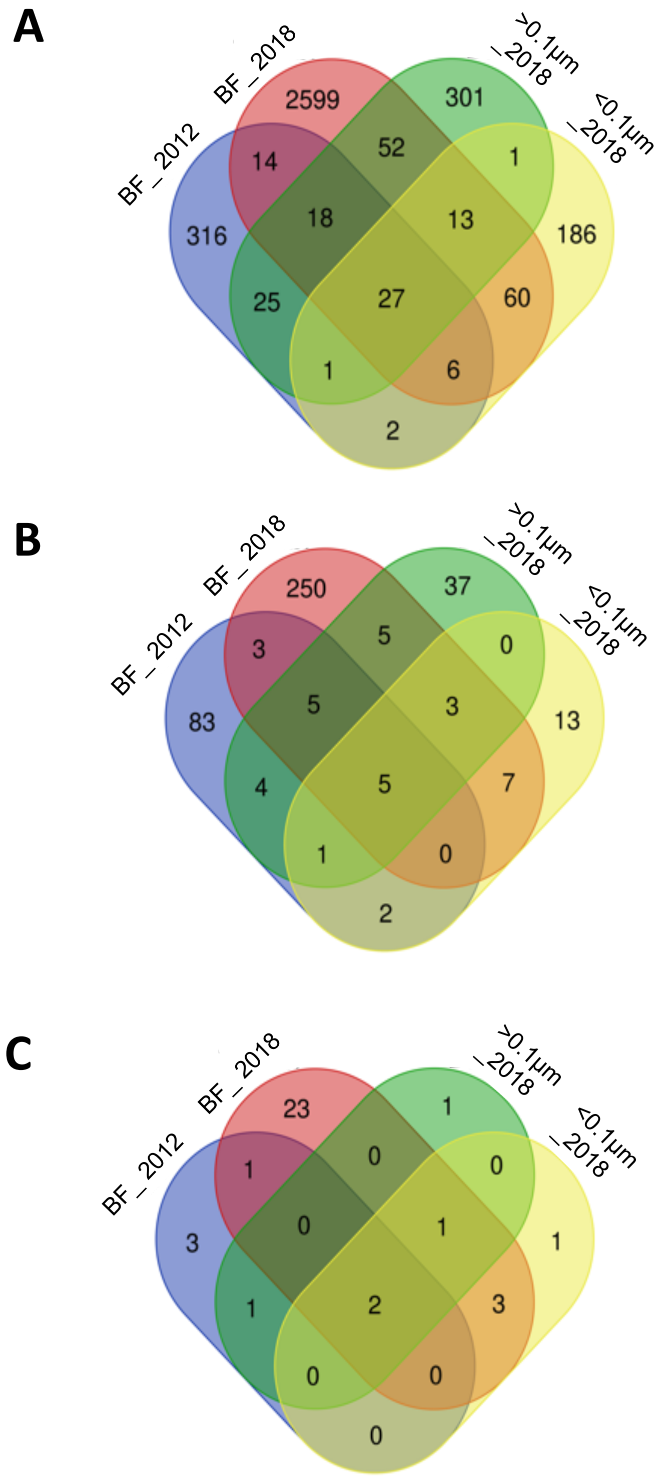

Supplementary Figure 16: VirusFISH of a dense Altiarchaeota biofilm (BF) flock showing no detectable infection by Altivir_1_MSI. Based on our enumeration, the majority of Altiarchaeota BF were infected by Altivir_1_MSI, however two out of seventeen BF flocks hybridized with the Altivir_1_MSI probe mix showed no infections at all. BF material was analyzed with A) DAPI (blue, cells), B) ATTO 488 (purple, 16S rRNA signal) and C) Alexa 594 (yellow, viral genomes of Altivir_1_MSI) and merged (D). For a statement regarding reproducibility please see legend of Supplementary Figure 11. Scale bars: 10 µm.

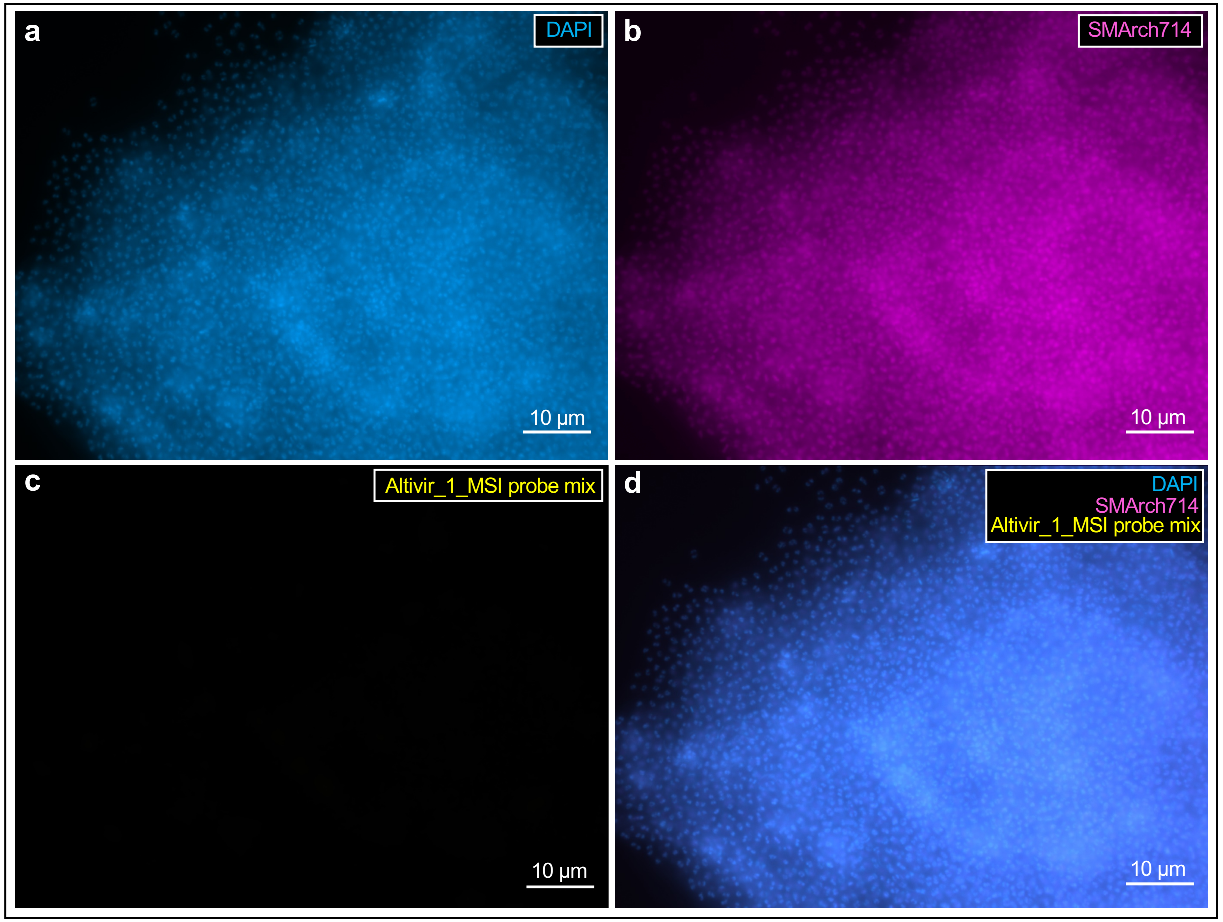

Supplementary Figure 17: EPI-fluorescence micrograph illustrating a heavily infected flock caused by Altivir_1_MSI. For virusFISH, a chemically synthesized probe mix targeting Altivir_1_MSI was used. Biofilm material was analyzed with A) DAPI (blue, cells), B) ATTO 488 (purple, 16S rRNA signal) and C) Alexa 594 (yellow, viral genomes of Altivir_1_MSI) and merged. For a statement regarding reproducibility please see legend of Supplementary Figure 11. Scale bars: 10 µm.

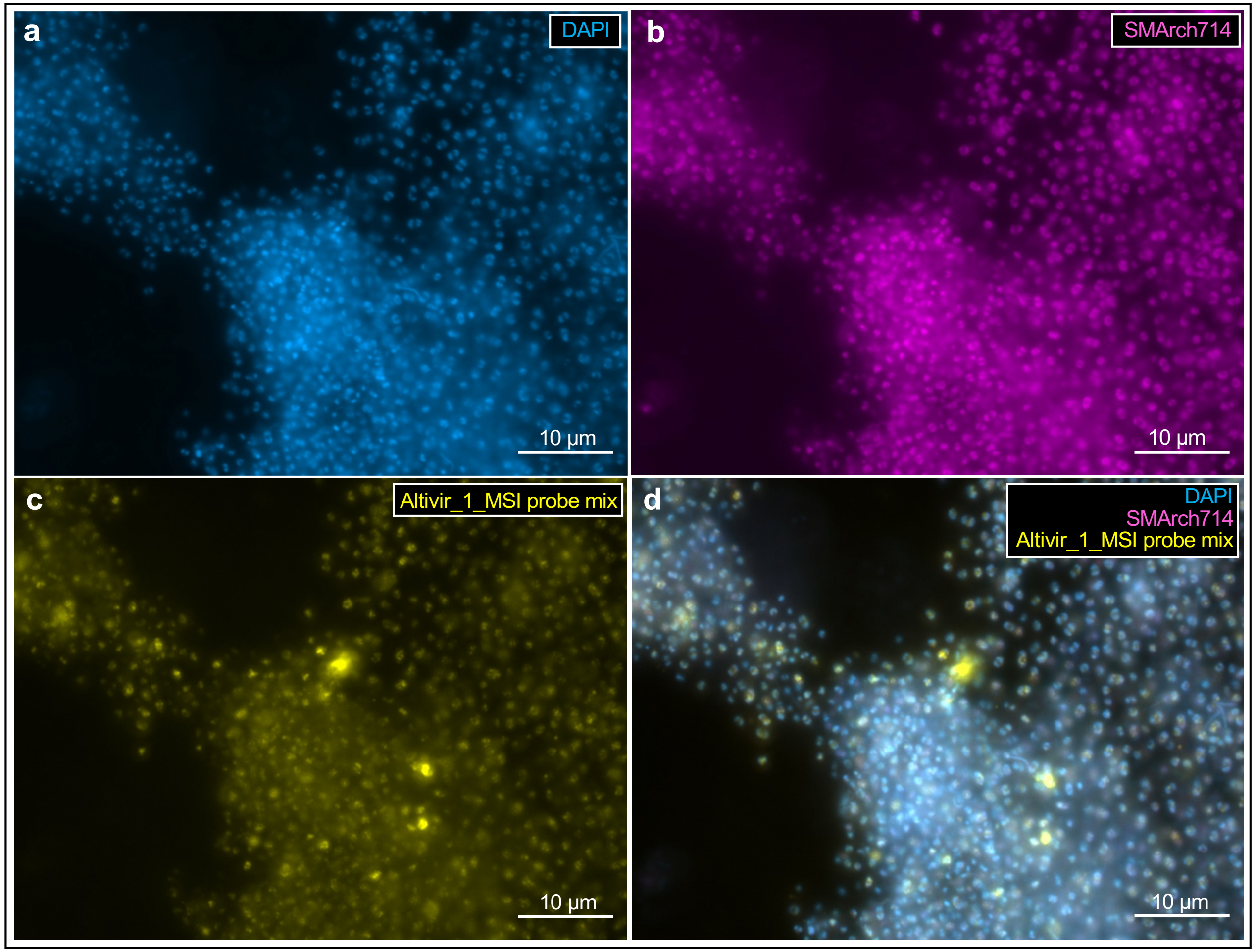

Supplementary Figure 18: EPI-fluorescence micrograph showing a heavily infected flock containing some rod-shaped bacteria potentially benefitting from lysing Altiarchaeota cells. For virusFISH, a chemically synthesized probe mix targeting Altivir_1_MSI was used. Biofilm material was analyzed with A) DAPI (blue, cells), B) ATTO 488 (purple, 16S rRNA signal) and C) Alexa 594 (yellow, viral genomes of Altivir_1_MSI) and merged (D). For a statement regarding reproducibility please see legend of Supplementary Figure 11. Scale bars: 10 µm. Scale bars: 10 µm.

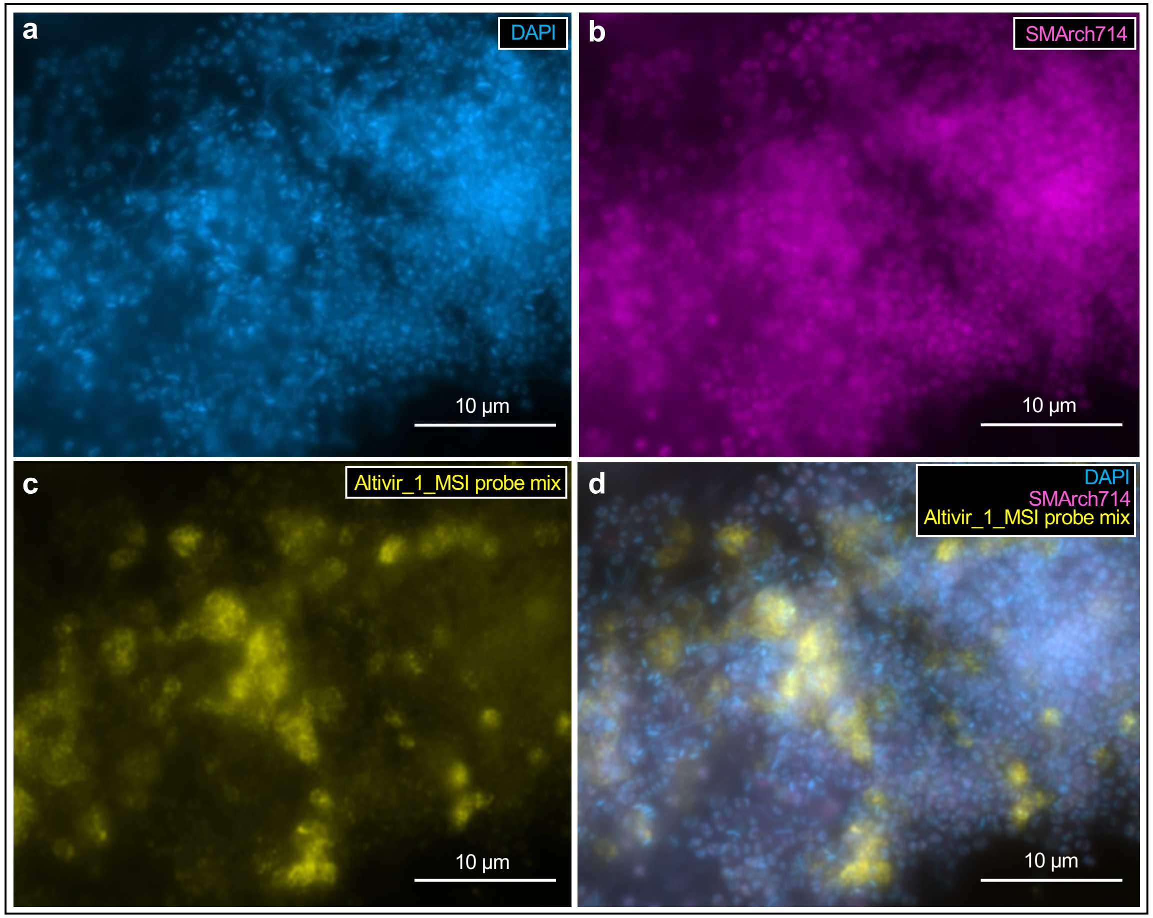

Supplementary References

1. Rudolph C, Moissl C, Henneberger R, Huber R. Ecology and microbial structures of archaeal/bacterial strings-of-pearls communities and archaeal relatives thriving in cold sulfidic springs. *FEMS Microbiol Ecol* **50**, 1-11 (2004).

2. Nurk S, Meleshko D, Korobeynikov A, Pevzner PA. metaSPAdes: a new versatile metagenomic assembler. *Genome Res* **27**, 824-834 (2017).

3. Edgar RC. MUSCLE: multiple sequence alignment with high accuracy and high throughput. *Nucleic Acids Res* **32**, 1792-1797 (2004).

4. Hofacker IL. Vienna RNA secondary structure server. *Nucleic Acids Res* **31**, 3429-3431 (2003).

5. Roux S, Enault F, Hurwitz BL, Sullivan MB. VirSorter: mining viral signal from microbial genomic data. *PeerJ* **3**, e985 (2015).

6. Ren J, Ahlgren NA, Lu YY, Fuhrman JA, Sun F. VirFinder: a novel k-mer based tool for identifying viral sequences from assembled metagenomic data. *Microbiome* **5**, 69 (2017).

7. Marz M*, et al.* Challenges in RNA virus bioinformatics. *Bioinformatics* **30**, 1793-1799 (2014).

8. Crits-Christoph A*, et al.* Functional interactions of archaea, bacteria and viruses in a hypersaline endolithic community. *Environ Microbiol* **18**, 2064-2077 (2016).

9. Eßer S. Dynamics of Altiarchaeota and their putative viruses in a deep terrestrial subsurface ecosystem. In: *Department of Chemistry*). University of Duisburg-Essen (2019).

10. Dabney A, Storey JD, Warnes G. qvalue: Q-value estimation for false discovery rate control. *R package version* **1**, (2010).

11. Team RC. R: A language and environment for statistical computing, R Foundation for Statistical Computing, Vienna, Austria. URL https://www.R-project.org/. (2019).

12. Moraru C, Varsani A, Kropinski AM. VIRIDIC-A novel tool to calculate the intergenomic similarities of prokaryote-infecting viruses. *Viruses* **12**, (2020).

13. Brister JR, Ako-Adjei D, Bao Y, Blinkova O. NCBI viral genomes resource. *Nucleic Acids Res* **43**, D571-577 (2015).

14. Bin Jang H*, et al.* Taxonomic assignment of uncultivated prokaryotic virus genomes is enabled by gene-sharing networks. *Nat Biotechnol* **37**, 632-639 (2019).

15. Bolduc B, Jang HB, Doulcier G, You ZQ, Roux S, Sullivan MB. vConTACT: an iVirus tool to classify double-stranded DNA viruses that infect Archaea and Bacteria. *PeerJ* **5**, e3243 (2017).

16. Shannon P*, et al.* Cytoscape: a software environment for integrated models of biomolecular interaction networks. *Genome Res* **13**, 2498-2504 (2003).

17. Kearse M*, et al.* Geneious Basic: an integrated and extendable desktop software platform for the organization and analysis of sequence data. *Bioinformatics* **28**, 1647-1649 (2012).

18. Langmead B, Salzberg SL. Fast gapped-read alignment with Bowtie 2. *Nat Methods* **9**, 357-359 (2012).
