# Supplementary material for: Lytic archaeal viruses infect abundant primary producers in Earth’s crust
Source: Nat Commun. 2021 Jul 30;12:4642. doi: 10.1038/s41467-021-24803-4 (PMC8324899; doi:10.1038/s41467-021-24803-4)
Supplement: Supplementary file 2 — Description of Additional Supplementary Information [file 41467_2021_24803_MOESM2_ESM.pdf]

Description of Additional Supplementary Files

File Name: Supplementary Data 1
Description: CRISPR Cas systems of highly abundant Altiarchaeota. CRISPR-associated proteins based on UniRef100 [1] annotations in assemblies and based on searches for a combination of the key words “Alti”, “Cas”, and “Alti”, “CRISPR”. The table also contains information on CRISPR arrays and associated Cas proteins detected by CRISPRcasFinder [2] in Altiarchaeota bins. ND=non detected.

File Name: Supplementary Data 2
Description: Variants derived by mapping reads from MSI biofilm (BF) 2012 and 2018 samples to 2012 genomes of Altivir_1_MSI and Altivir_2_MSI, respectively. Details on single nucleotide and other polymorphisms are given together with the information if variants affect a region matched by a spacer. Analysis has been conducted in Geneious Prime version v.11.1.5 [3].

File Name: Supplementary Data 3
Description: Protein annotations of open reading frames on Altiarchaeota virus genomes. Functional annotations were performed via HHpred (https://toolkit.tuebingen.mpg.de/tools/hhpred) [4, 5] against PDB_mmCIFC70_4_Feb, Pfam-A v.32.0, NCBI_Conserved_Domains_v3.16 and TIGRFAMs v15.0 database. Moreover, DIAMOND v.0.9.9 against UniRef100 (Feb. 2018) [1], HMM searches against Virus Orthologous Groups from VOGDB[6], HHblits v3.3.0 against pVOGs [7] and InterProScan against InterPro database 82.0. For the most abundant viruses, e.g., Altivir_1_MSI and Altivir_8_HURL, PHMMER (PH, https://www.ebi.ac.uk/Tools/hmmer/search/phmmer) against reference proteomes [8], and DELTA-BLAST (DB) [9] against NR were additionally used. MSI=Mühlbacher Schwefelquelle, Isling, ACLF=Alpena County Library Fountain, HURL=Horonobe Underground Research Laboratory, GA=Geyser Andernach
