# Supplementary material for: Lytic archaeal viruses infect abundant primary producers in Earth’s crust
Source: Nat Commun. 2021 Jul 30;12:4642. doi: 10.1038/s41467-021-24803-4 (PMC8324899; doi:10.1038/s41467-021-24803-4)
Supplement: Supplementary file 6 — Reporting Summary [file 41467_2021_24803_MOESM6_ESM.pdf]

nature research

Corresponding author(s): Alexander J. Probst

Last updated by author(s): May 27, 2021

Reporting Summary

Nature Research wishes to improve the reproducibility of the work that we publish. This form provides structure for consistency and transparency in reporting. For further information on Nature Research policies, see our Editorial Policies and the Editorial Policy Checklist.

Statistics

For all statistical analyses, confirm that the following items are present in the figure legend, table legend, main text, or Methods section.

n/a	Confirmed	
[ ]	[x]	The exact sample size (n) for each experimental group/condition, given as a discrete number and unit of measurement
[ ]	[x]	A statement on whether measurements were taken from distinct samples or whether the same sample was measured repeatedly
[ ]	[x]	The statistical test(s) used AND whether they are one- or two-sided<br>*Only common tests should be described solely by name; describe more complex techniques in the Methods section.*
[x]	[ ]	A description of all covariates tested
[x]	[ ]	A description of any assumptions or corrections, such as tests of normality and adjustment for multiple comparisons
[x]	[ ]	A full description of the statistical parameters including central tendency (e.g. means) or other basic estimates (e.g. regression coefficient) AND variation (e.g. standard deviation) or associated estimates of uncertainty (e.g. confidence intervals)
[ ]	[x]	For null hypothesis testing, the test statistic (e.g. F, t, r) with confidence intervals, effect sizes, degrees of freedom and P value noted<br>*Give P values as exact values whenever suitable.*
[x]	[ ]	For Bayesian analysis, information on the choice of priors and Markov chain Monte Carlo settings
[x]	[ ]	For hierarchical and complex designs, identification of the appropriate level for tests and full reporting of outcomes
[x]	[ ]	Estimates of effect sizes (e.g. Cohen's d, Pearson's r), indicating how they were calculated

Our web collection on statistics for biologists contains articles on many of the points above.

Software and code

Policy information about availability of computer code

Data collection

We used the following code and programs for our analyses (method->software):
-design of virusFISH probes -> genePROBER (gene-prober.icbm.de/)
-Fluorescence microscopy -> Zen 2 Pro software (version 2.0.0.0) (Carl Zeiss Microscopy GmbH, Jena, Germany)
-SIM -> Zen 2.3 SP1 FP3 black (version 14.0.22.201)
-TEM -> ImageSP software (version 1.2.9.77) (x64) (SysProg, Minsk, Belarus)
-Metagenome search for Altiarchaeota -> IMG/M database (database accessed in July 2018)
-Read trimming and quality-filtration -> bbduk (https://github.com/BioInfoTools/BBMap/blob/master/sh/bbduk.sh) and Sickle v. 1.33.
-Read assembly -> metaSPADes v.3.10
-Gene annotation -> Prodigal v.2.6.3
-Gene annotation -> DIAMOND v.0.9.9 against UniRef100 (Feb. 2018).
-detection of mini-CRISPR arrays on viral scaffolds -> CRISPRCasFinder Web-service (https://crisprcas.i2bc.paris-saclay.fr/).
-secondary structure of CRISPR DR sequences -> RNAfold (http://rna.tbi.univie.ac.at/cgi-bin/RNAWebSuite/RNAfold.cgi) and checked against the CRISPRmap database (v2.1.3-2014)
-extraction of CRISPR spacers -> MetaCRAST.
-clustering of spacers -> Cd-hit v. 4.6
-Viral identification -> Endmatcher (https://github.com/ProbstLab/viromics/tree/master/Endmatcher), VirSorter v1, VirFinder v.1.1, VOGDB (vog93, April 2019) and CircMG (https://github.com/alexcritschristoph/VRCA) .
-Functional annotations of viral genes -> BLASTp and DELTA-BLAST, prokaryotic Viruses Orthologous Groups (pVOGs) with HHblits 3.3.0., HMMER against VOGDB (vog93, April 2019), InterPro database 82.0 with InterProScan integrated in Geneious prime 2020, UniRef100 (Feb. 2018) with DIAMOND, PDB_mmCIFC70_4_Feb, Pfam-A v.32.0, NCBI_Conserved_Domains_v3.16 and TIGRFAMs v15.0 database with HHpred (https://toolkit.tuebingen.mpg.de/tools/hhpred), NCBI's non-redundant protein sequences (nr) with DELTA-BLAST (https://blast.ncbi.nlm.nih.gov/Blast.cgi?PAGE_TYPE=BlastSearch&PROGRAM=blastp&BLAST_PROGRAMS=deltaBlast) and reference proteomes with

phmmer (https://www.ebi.ac.uk/Tools/hmmer/search/phmmer)
-Viral clustering -> VICTOR (https://ggdc.dsmz.de/victor.php), vCONTACT v. 0.9.11 and VIRIDIC (http://viridic.icbm.de /)
-Network visualisation -> Cytoscape version 3.7.2
-Plotting of genomic maps -> genoPlotR v.0.8.9 package (R programming environment v.3.5.2)
-Protein alignment -> COBALT (https://www.ncbi.nlm.nih.gov/tools/cobalt/re_cobalt.cgi) followed by refinement using MUSCLE 3.8.425 in Geneious prime 2020.
-Tree for Altivir_2_MSI -> FastTree 2.1.12 in Geneious prime 2020
-Trees for Altivir_4_ACLF, Altivir_7_ACLF and Altivir_8_HURL -> webserver https://www.phylogeny.fr/ after alignment curation with Gblocks. Tree was reconstructed using PhyML, visualized in FigTree v1.4.3
-Venn diagrams -> VIB/Ugent web tool (http://bioinformatics.psb.ugent.be/webtools/Venn/)
-World map -> Ocean Data View 5.3.0 (https://odv.awi.de/).
-Alignment of DR -> MUSCLE in Geneious 11.1.5
-Visualization of SNPs, spacer matches, CDS alignments of viral genomes (Suppl Fig 10/14) -> Geneious 11.1.5

Data analysis

Software code and web links are available from online sources and appropriately referenced to in the method section. A description of how we perfomed the analysis and which software version we used can be found in respective methods part.

For manuscripts utilizing custom algorithms or software that are central to the research but not yet described in published literature, software must be made available to editors and reviewers. We strongly encourage code deposition in a community repository (e.g. GitHub). See the Nature Research guidelines for submitting code & software for further information.

Data

Policy information about availability of data

All manuscripts must include a data availability statement. This statement should provide the following information, where applicable:

- Accession codes, unique identifiers, or web links for publicly available datasets
- A list of figures that have associated raw data
- A description of any restrictions on data availability

Sequencing data generated and analyzed during this study are available at NCBI's sequence read archive and GenBank with accession codes as listed in Supplementary Table 1. Metagenomic reads are available from NCBI Bioprojects #PRJNA628506, Biosamples #SAMN14733005-07, Run #SRR11614986-88 (MSI_2018), Bioproject #PRJNA321556, Biosample #SAMN04999996-97, Run #SRR3546456-57 (HURL), Bioproject #PRJNA627655 (GA), Biosample #SAMN14680028-30, Run #SRR11600161-63 (GA), Bioproject #PRJNA340050, Biosample #SAMN05661201, Run #SRR4293692 (ACLF) as well as from ENA #PRJNA678866, Biosample #SAMEA2779769, Run accession #ERR628383 (MSI_2012). Metagenomic assemblies can be found under the above-mentioned Bioprojects/samples for MSI_2018 and GA. Exceptions represented MSI_2012, which was deposited under Bioproject #PRJNA678866, Biosample #SAMN16815598, HURL assemblies under Bioproject #PRJNA730881, Biosample #SAMN04999996-7, and the ACLF assembly under Bioproject #PRJNA730879, Biosample #SAMN05661201. Run accessions for these assemblies are JAEMOC000000000-JAEMOL000000000. Ca. Altiarchaeota genomes were deposited at GenBank under Bioproject #PRJNA628506, MAG Biosample #SAMN18220766, Run #JAGTWS000000000 (MSI_2018), Bioproject #PRJNA627655, MAG Biosamples #SAMN18220852-54, Run #JAGTWP000000000-JAGTWR000000000 (GA), Bioproject #PRJNA726854, MAG Biosample #SAMN18221259, Run #JAGWDR000000000 (ACLF), Bioproject #PRJNA726852, MAG Biosample #SAMN18220774-75, Run #JAGWDQ000000000 & #JAGWDP000000000 (HURL), and at ENA under #CCXY01000000 (MSI_2012). Viral genomes of Ca. Altiarchaeota Altivirus#1 and #2 can be found under GenBank accessions #MW522970 and #MW522971, respectively. Viral genomes of Ca. Altiarchaeota Altivirus#3-8 can be accessed via the scaffold accession numbers as given in Supplementary Table 4. Viral genomes can also be found at https://github.com/ProbstLab/viromics/tree/master/viruses/Altivir.
Microscopy data can be obtained from the corresponding author upon reasonable request.

Field-specific reporting

Please select the one below that is the best fit for your research. If you are not sure, read the appropriate sections before making your selection.

☐ Life sciences ☐ Behavioural & social sciences ☒ Ecological, evolutionary & environmental sciences

For a reference copy of the document with all sections, see nature.com/documents/nr-reporting-summary-flat.pdf

Ecological, evolutionary & environmental sciences study design

All studies must disclose on these points even when the disclosure is negative.

Study description

This study was set out with the aim of elucidating viral infections of Ca. Altiarchaea across the globe using in silico (sequencing-based) methods as well as microscopy methods.

Research sample

Public datasets were searched for Ca. Altiarchaea and chosen based on a hit with respective hamus proteins and manually selected based on available data structure. One sampling site was chosen to further elucidate the predicted host-virus relationships based on accessibility and biomass of the site: We collected microbial biofilm and water samples from an aquifer (sulfidic spring Mühlbacher Schwefelquelle Isling, MSI) that contained uncultivated archaea (mainly Altiarchaea) and a small proportion of bacteria. There was no further selection of samples for metagenomics or microscopy: For metagenomics, we used bulk samples that contained hundreds of biofilm flocks or dozens of liters of groundwater for filtration; for microscopy, biofilm flocks from the aquifer were randomly chosen after sampling.

Sampling strategy

For sampling the unfiltered planktonic microbial community for metagenomics, 70 L of groundwater were filtered onto a 0.1 µm pore-size PTFE membrane filter (Merck Millipore, Darmstadt, Germany). The flow-through was collected in a sterilized container, and a final concentration of 1 mg L-1 of iron (III) chloride (Carl Roth, Karlsruhe, Germany) was applied for chemical flocculation (30

	minutes). Flocculates were filtered onto 5 x 0.2 µm membrane filters (<0.1 µm fraction, viral fraction).<br>We chose to sample the archaeal biofilms, planktonic and viral fractions to determine viral infestations for planktonic and sessile Altiarchaeota. Metagenomes from whole community DNA reflected temperate and actively replicating viruses, whereas the viral fraction (virome) predominantly contained free viruses and lysed (Altiarchaeota) cells remnants. Sequencing viromes generally also increases the likelihood of finding rare viruses.<br>For virusFISH we first established the method with respective controls and then randomly took 18 individual biofilm flocks from the ecosystem for determining infection categories of the 8.9kb long, dominant virus. There was no selection of biofilm flocks based on certain parameters.
Data collection	Groundwater for metagenomic data of the Mühlbacher Schwefelquelle, Isling, Germany were collected 15th to 17th October 2018 in presence of Perla Abigail Figueroa Gonzalez, Janina Rahlff and Alexander Probst. Three types of samples were collected for metagenomics: i) biofilm flocks; ii) the planktonic community (>0.1 µm pore-size fraction); and iii) the viruses and lysed cells (<0.1 µm pore-size fraction) using a custom-made filtration system (serial number SN 0604_2018) by Sima-Tec (Schwalmtal, Germany) and 142 mm diameter filter membranes. Shotgun metagenome sequencing was performed using an Illumina HiSeq platform.<br><br>Biofilm material for virusFISH was collected 17th to 19th January 2019 by Victoria Turzynski, Indra Monsees and Alexander Probst. Detailed information on the sampling procedure for data collection can be found in the manuscript. Biofilm material was examined with an epifluorescence microscope Axio imager M2m equipped with an Axio Cam MRm (Carl Zeiss Microscopy GmbH, Germany). Zen 2 Pro Software (version 2.0.0.0) was used for achieving all micrographs (Carl Zeiss Microscopy GmbH, Germany). The following filter sets were used for visualizing Altiarchaeota biofilms and their viral infections; 49 DAPI shift free (488049-9901-000), 64 He mPlum shift free (489064-0000-000) and 09 shift free (488009-9901-000) (Carl Zeiss Microscopy GmbH, Germany).<br>Structured illumination microscopy was performed on the biofilm samples (taken in January 2019, see above) with a Zeiss ELYRA PS.1 epifluorescence microscope equipped with a F Set 77 He filter (Carl Zeiss Microscopy GmbH, Germany). For analyzing the biofilm material, a Zen 2.3 SP1 FP3 black (version 14.0.22.201) was used (Carl Zeiss Microscopy GmbH, Germany).<br><br>Transmission electron microscopy was carried out by Andreas Klingl on biofilm samples taken on 17th to 19th October 2018. The biofilm material was observed with a Zeiss EM 912 Carl Zeiss Microscopy GmbH, Germany) equipped with an integrated OMEGA-filter and a 2k x 2k pixel slow-scan CCD camera (TRS Tröndle Restverstärkersysteme, Moorenweis, Germany). ImageSP software (version 1.2.9.77) (64x) (SysProg, Minsk, Belarus) was used.
Timing and spatial scale	Groundwater for metagenomic data of the Mühlbacher Schwefelquelle, Isling, Germany were collected 15th to 17th October 2018. Biofilm material for TEM analysis was collected on 17th to 19th October 2018 and for virusFISH from 17th to 19th January 2019.
Data exclusions	We do not show the data and analysis of non-matching spacers derived from viral mini CRISPR arrays that we checked for their role in interviral conflicts, because the spacers did not match any viral targets and there is consequently no data to show.
Reproducibility	We initially identified the target virus for virusFISH in public metagenomes from 2012. To reproduce the identification and reconstruction of the virus and of the virus-host relationship from the metagenome, we did not perform technical replicates but biological replicates by focusing on size fractionation of the biological samples: We performed metagenomics of a biofilm sample, from the planktonic fraction in the community and from the viral fraction, for all of which we were able to recover the respective virus.<br><br>Detailed information can be found in the method section of the manuscript. FISH experiments were highly reproducible: Since we established virusFISH for this project in 2019, we have tested the chemically synthesized probe mix targeting Altivir_1_MSI 70 times in total and the Metallosphaera sp. virus probe (as negative control) 66 times in total. The testing was always carried out on Altiarchaea biofilm samples from MSI site.
Randomization	During randomization experimental subjects are assigned to different treatments, but in this study we did not compare different treatments in that sense.
Blinding	Blinding is usually performed in experiments involving living participants, which are kept unaware of the treatment group they get assigned to. This was not applicable in our study as human participants were not part of the experiments. Blinding during virusFISH experiments was not applicable, because the experimenter needs to know which biofilm flock was treated with positive and negative probes.

Did the study involve field work? ☒ Yes ☐ No

Field work, collection and transport

Field conditions	Groundwater was sampled from a ca. 10 degree celsius cold aquifer, no rainfall, pH ca. 7.2.
Location	Samples were collected for microscopy and metagenomic analyzes from the sulfidic spring Mühlbacher Schwefelquelle Isling (MSI) in Regensburg, Germany (N 48° 59.142, E 012° 07.636). Data from other ecosystems were obtained from public databases or sampled and reported within another study as referenced in the manuscript.
Access & import/export	No import or export of samples was conducted as the sampling site is in Germany and the executing lab was also located in Germany. Samples from the aquifer were taken after local authorities (city of Regensburg) had approved the sampling in the naturally protected area. Permission for sampling the Mühlbacher Schwefelquelle was granted on 29th June 2018 by Mr. Forster, Umweltamt Stadt Regensburg (Az. 31.4 Fo) and is valid for 5 years.
Disturbance	The sampling was non-invasive to the ecosystem as upwelling groundwater has been sampled.

Reporting for specific materials, systems and methods

We require information from authors about some types of materials, experimental systems and methods used in many studies. Here, indicate whether each material, system or method listed is relevant to your study. If you are not sure if a list item applies to your research, read the appropriate section before selecting a response.

Materials & experimental systems

n/a	Involved in the study
☒	☐ Antibodies
☒	☐ Eukaryotic cell lines
☒	☐ Palaeontology and archaeology
☒	☐ Animals and other organisms
☒	☐ Human research participants
☒	☐ Clinical data
☒	☐ Dual use research of concern

Methods

n/a	Involved in the study
☒	☐ ChIP-seq
☒	☐ Flow cytometry
☒	☐ MRI-based neuroimaging
